# Global burden of MASLD-IBD comorbidity from 1990 to 2021 and trend prediction to 2050

**DOI:** 10.1097/JS9.0000000000003383

**Published:** 2025-10-13

**Authors:** Yuanshan Xu, Youting Yi, Xiankang Zhang, Boyi Jia, Yanrong Huang, Renyun Cui, Haowen Sun, Meng Wang, Jin-Yi Wan, Haiqiang Yao, Chun-Su Yuan

**Affiliations:** a School of Traditional Chinese Medicine, Beijing University of Chinese Medicine, Beijing, China; b Department of Gastroenterology, Fangshan Traditional Medical Hospital of Beijing, Beijing, China; c National Institute of TCM Constitution and Preventive Medicine, Beijing University of Chinese Medicine, Beijing, China; d Tang Center for Herbal Medicine Research, The University of Chicago, Chicago, IL, USA; e Department of Anesthesia and Critical Care, The University of Chicago, Chicago, IL, USA

**Keywords:** epidemiology, global, IBD, MASLD, prevalence

## Abstract

**Background:**

Metabolic dysfunction-associated steatotic liver disease (MASLD), a prevalent chronic liver disorder, imposes a substantial global clinical and economic burden. Emerging evidence underscores inflammatory bowel disease (IBD) as a significant independent risk factor for MASLD. However, the global burden and trends of MASLD-IBD comorbidity remain largely obscured. This study aims to evaluate the global burden of MASLD-IBD from 1990 to 2021 and to project its trajectory to 2050. The findings will inform the development of proactive public health strategies

**Methods:**

Leveraging the Global Burden of Disease (GBD) 2021 database, we quantified IBD’s contribution to MASLD via population-attributable fractions. We analyzed the global prevalence, mortality, and disability-adjusted life years (DALYs), stratifying by socio-demographic index (SDI), sex, and age. Estimated annual percentage change (EAPC) assessed 1990–2021 trends; Bayesian age-period-cohort modeling projected 2050 trajectories.

**Results:**

In 2021, the global age-standardized prevalence of MASLD-IBD comorbidity reached 171.7 cases (95% uncertainty interval [UI], 157.3–187.1), with a corresponding DALY rate of 0.5 cases (95% UI, 0.4–0.6) per 10 million population. High-income regions, including North America, Australia, and Western Europe, bore the highest burden. From 1990 to 2021, 20 out of 21 GBD regions exhibited escalating age-standardized prevalence and DALY rates, most markedly in East Asia (prevalence EAPC = 3.18; 95% confidence interval [CI], 2.59–3.77) and East Europe (DALY EAPC = 4.05; 95% CI, 3.32–4.79). Middle SDI regions saw the largest absolute increases, with mortality mirroring DALY trends. Adults ≥ 60 years bore disproportionately higher burdens. By 2050, projections suggest divergent gender trends of rising female burden versus declining male rates.

**Conclusions:**

MASLD-IBD comorbidity burdens have risen globally over three decades, necessitating targeted interventions, particularly for ageing populations and high-risk regions. Gender-specific strategies will be critical for mitigating future impacts.

**HIGHLIGHTS**

- First global quantification: Leveraging the Global Burden of Disease 2021 database, we provide the first comprehensive analysis of MASLD-IBD comorbidity across 204 countries, revealing a 2021 age-standardized prevalence of 171.7 cases/10 million population – a burden concentrated in high-income regions (North America, Australia, and Western Europe).
- Emerging epidemiological shifts: Our identification of sharply rising trends in East Asia (prevalence EAPC = 3.18) and Eastern Europe (disability-adjusted life year EAPC = 4.05) signals shifting disease hotspots, while projections to 2050 uncover a critical gender divergence: rising female burden contrasts with declining male rates.
- Actionable public health insights: The disproportionate burden among aging populations (adults ≥60 years) and middle SDI regions underscores urgent needs for stratified interventions.

## Introduction

Mounting evidence highlights the critical public health challenge posed by metabolic dysfunction-associated steatotic liver disease (MASLD), a leading chronic hepatic disorder worldwide with a global prevalence of 38%^[1–5]^. Emerging research underscores inflammatory bowel disease (IBD) as a critical independent and underappreciated risk factor for MASLD development, with their interaction forming a distinct clinical entity marked by bidirectional inflammatory pathways and shared metabolic dysregulation^[6,7]^. MASLD, characterized by excessive fat deposition in liver cells, can progress to liver fibrosis, cirrhosis, and even hepatocellular carcinoma, posing a significant threat to individual health and placing a heavy burden on global healthcare systems^[8,9]^. IBD is an immune-mediated chronic systemic inflammatory disease with complex extraintestinal manifestations^[10]^, including considerable liver involvement^[11]^. Emerging evidence highlights IBD as a crucial pathogenic driver of MASLD, and epidemiological trends now reveal a concerning rise in MASLD-IBD comorbidity^[12]^, attributed to multifactorial mechanisms including genetic predisposition, gut dysbiosis, metabolic derangements, and iatrogenic effects^[13–17]^.

The comorbidity of MASLD and IBD exacerbates the pathological damage to both the liver and gut while intensifying systemic metabolic dysfunction and inflammatory cascades via the gut–liver axis^[18]^. This bidirectional interaction establishes a vicious cycle, making patients more prone to various complications and leading to worse overall outcomes^[19–22]^. Notably, compared with non-IBD-related MASLD, IBD-related MASLD exhibits enhanced inflammatory responses and increased oxidative stress^[23,24]^, leading to faster progression and higher rates of liver fibrosis^[25,26]^. However, despite these risks, current research lacks comprehensive, population-level assessments of the global burden imposed by MASLD-IBD comorbidity. Addressing this gap is critical to elucidating epidemiological patterns, modifiable risk factors, and mechanisms driving this comorbidity. Such insights are urgently needed to inform evidence-based interventions, optimize resource allocation, and mitigate the dual burden of metabolic and inflammatory morbidity through targeted public health strategies^[27]^.

Leveraging the Global Burden of Disease (GBD) 2021 database, this study provides a comprehensive assessment of the global burden of MASLD-IBD comorbidity across 204 countries and territories from 1990 to 2021, and predictions to 2050. Prevalence served as the primary epidemiological metric, complemented by disability-adjusted life years (DALYs) to quantify health loss at global levels. Socio-demographic index (SDI) stratification was employed to evaluate disparities in disease burden across development gradients, with further subgroup analyses by sex and age to identify vulnerable populations. In accordance with the GBD Protocol and the TITAN Guidelines 2025^[28]^, this study delivers robust, policy-relevant evidence to guide the formulation of public health strategies for MASLD management, particularly in addressing the escalating challenge of comorbid MASLD-IBD through targeted prevention, resource prioritization, and integrated care frameworks.

## Methods

### Data source

The data for this study were sourced from the 2021 GBD database constructed by the Institute for Health Metrics and Evaluation. This database encompasses epidemiological data for 371 diseases and injuries across 204 countries and territories from 1990 to 2021^[29]^. Utilizing the Global Health Data Exchange query tool (https://ghdx.healthdata.org/gbd-2021), we extracted key metrics such as prevalence, DALYs, and mortality rates for IBD and MASLD from 1990 to 2021. Definitions of the diseases and the retrieval strategy are detailed in the Supplemental Digital Content, available at: http://links.lww.com/JS9/F289. The GBD database employs a multitiered classification system: geographically, the 204 countries are categorized into 21 regions^[29]^; socioeconomically, countries are stratified using the SDI, which integrates three indicators – per capita income, educational attainment, and total fertility rate – to classify countries into five tiers: high, high-middle, middle, low-middle, and low.

The primary analytical metrics of this study include the absolute numbers of prevalence, DALYs, and deaths, their corresponding rates (per 10 million population), and age-standardized rates (ASRs). The ASRs are standardized using the GBD standard world population, calculated as the weighted average of the age-specific incidence (or mortality) rates, with weights corresponding to the age structure of the standard population. This serves as a pivotal indicator for comparing disease burdens across different regions and periods. For a comprehensive understanding of the methodologies employed in the GBD 2021 study, reference is made to prior literature.

### Population-attributable fraction

Since the GBD database does not classify MASLD-IBD comorbidity as an independent disease entity, this study employed Levin’s formula (Eq. 1) to calculate the population-attributable fraction (PAF)^[30]^ for quantifying the contribution of comorbid IBD to the MASLD burden, thereby effectively avoiding double-counting. Additionally, a thorough Monte Carlo sensitivity analysis was conducted in the appendix to quantify the impact of parameter uncertainty via simulation.

$$PAF=\frac{p\times \left(RR-1\right)}{p\times \left(RR-1\right)+1}$$

Eq. (1)

The key variable in Equation 1 is the relative risk (RR), which represents the risk ratio of developing hepatitis in patients with MASLD-IBD comorbidity compared to those with MASLD alone. Through a systematic search of the Medline and Web of Science databases (Supplemental Digital Content Figure S1, available at: http://links.lww.com/JS9/F289), the RR value was determined to be 1.13 (95% confidence interval [CI], 1.04–1.24)^[31]^ based on a large-scale prospective cohort study involving 10 542 participants. The detailed search strategy is provided in the Supplemental Digital Content, available at: http://links.lww.com/JS9/F289.

Another critical parameter for PAF calculation is *p*, which is the prevalence of IBD comorbidity among patients with MASLD. This parameter was estimated using the ratio of IBD prevalence in the general population to that in patients with MASLD (1.96, 95% CI, 1.13–3.41)^[32]^, derived from a large meta-analysis incorporating 44 studies. Data on IBD prevalence in the general population were directly obtained from the GBD 2021 database. We assumed that the RR and *p* values remained constant across countries, years, and age-groups to ensure methodological consistency.

The study results revealed significant disparities in PAF values across the 21 GBD regions in 2021, ranging from 0.12‱ to 6.86‱. Detailed PAF values stratified by region, country, SDI level, sex, and age are presented in Supplemental Digital Content Table S3 and Supplemental Digital Content Figure S2, available at: http://links.lww.com/JS9/F289. These findings provide crucial quantitative evidence for formulating targeted prevention and control strategies for MASLD-IBD comorbidity.

### Global burden and trends estimation

In this study, we estimated the global disease burden attributable to MASLD-IBD comorbidity by multiplying the PAF with the disease burden metrics of MASLD, including prevalence, DALYs, and mortality. We systematically analyzed the disease burden and its trends for MASLD-IBD comorbidity at the global, regional, and national levels from 1990 to 2021, with stratification by SDI, sex, and age. The study primarily reports the age-standardized prevalence rates and DALY rates attributable to MASLD-IBD comorbidity, whereas the age-standardized mortality rates are detailed in the Supplemental Digital Content, available at: http://links.lww.com/JS9/F289. Additionally, we report the absolute numbers of prevalent cases, DALYs, and deaths associated with MASLD-IBD comorbidity.

To evaluate the trends in age-standardized prevalence, DALYs, and mortality rates for MASLD-IBD comorbidity, we employed the method proposed by Hankey *et al*^[33–35]^, using a generalized linear model with Gaussian distribution to fit the natural logarithm of ASRs against calendar year, where *α* is the intercept, *β* is the slope coefficient, and *ϵ* is the error term:



The estimated annual percentage change (EAPC) was then calculated as follows:

$$EAPC=100\times \left(e^{\beta }-1\right)$$

Eq. (2)

The 95% CI of EAPC was derived from the 95% CI, of *β*, where *β*_lower_ and *β*_upper_ are the lower and upper limits of *β*’s 95% CI.

$$95\%CI_{EAPC}=\left[100\times \left(e^{\beta_{lower}}-1\right),100\times \left(e^{\beta_{upper}}-1\right)\right]$$

Specifically, the *β* value was obtained by fitting a regression model, and the EAPC (95% CI) was derived from this *β* value (and its 95% CI). An increasing trend in ASR was inferred if the lower limit of the 95% CI, for the EAPC was greater than 0, a decreasing trend if the upper limit was less than 0, and a stable trend if the 95% CI, included 0. Furthermore, we calculated the relative percentage changes in the prevalent cases, DALYs, and deaths attributable to MASLD-IBD comorbidity from 1990 to 2021. All statistical analyses were performed using R software (version 4.4.2), with the significance level set at *P* < 0.05 (two-tailed test).

### Prediction analysis

To predict the age-standardized prevalence rates and DALYs from 2021 to 2050, we employed the Bayesian Age-Period-Cohort (BAPC) model, incorporating nested Laplace approximations. This approach leverages the relationship between prevalence or DALY rates and demographic structures and population sizes. The BAPC model advantages include its ability to approximate the posterior marginal distribution without direct convergence diagnostics and its high accuracy^[36]^. Compared to Generalized Additive Models, Smoothed Spline Models, Nordpred, and Poisson regression, the BAPC model demonstrates superior performance in predicting short-term and medium-term disease burdens^[37,38]^. The appendix provides more information on the fundamental principles of BAPC regarding good-fit assessment, cross-validation methods, and parameter selection, detailing the setup, assumptions, training process, and validation process of the BAPC model.

This study complies with the Guidelines for Accurate and Transparent Health Estimates Reporting (GATHER) statement^[39]^ (Supplemental Digital Content Table S2, available at: http://links.lww.com/JS9/F289).

## Results

### Global burden of MASLD-IBD Comorbidity in 2021

**Figure 1.**
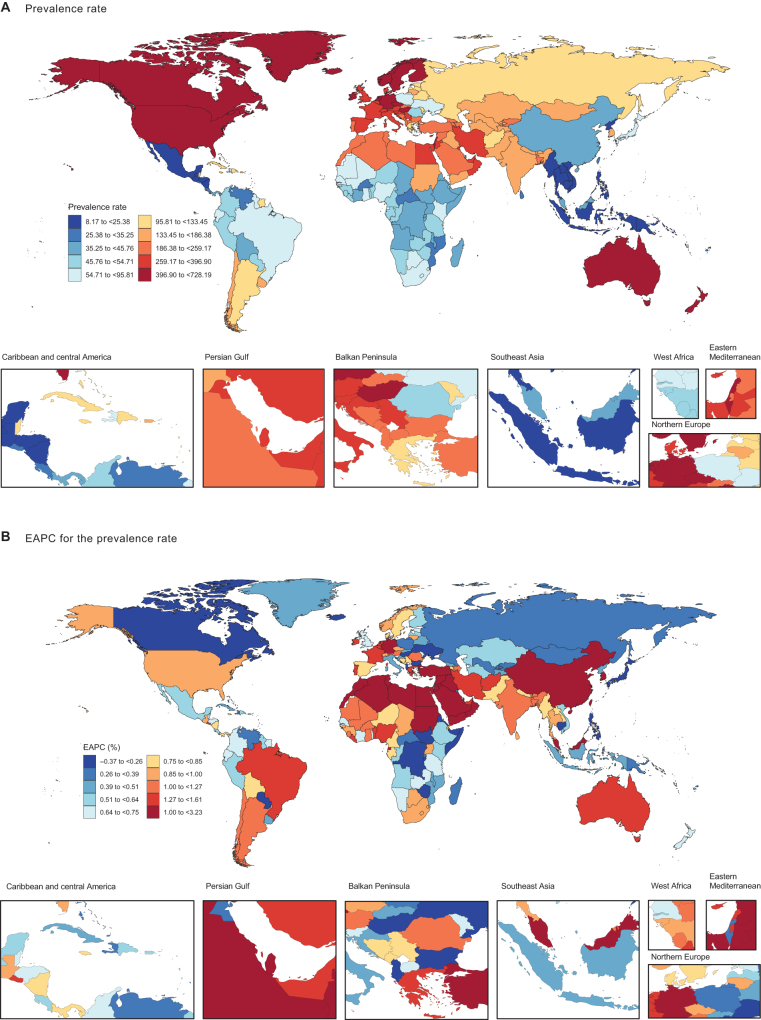
The age-standardized prevalence (A) rate (per 10 million individuals) of MASLD–IBD comorbidity in 204 countries and territories in 2021; EAPC in the age-standardized prevalence (B) rate of MASLD–IBD comorbidity in 204 countries and territories from 1990 to 2021. MASLD, metabolic dysfunction-associated steatotic liver disease; IBD, inflammatory bowel disease; EAPC, estimated annual percentage change.

**Figure 2.**
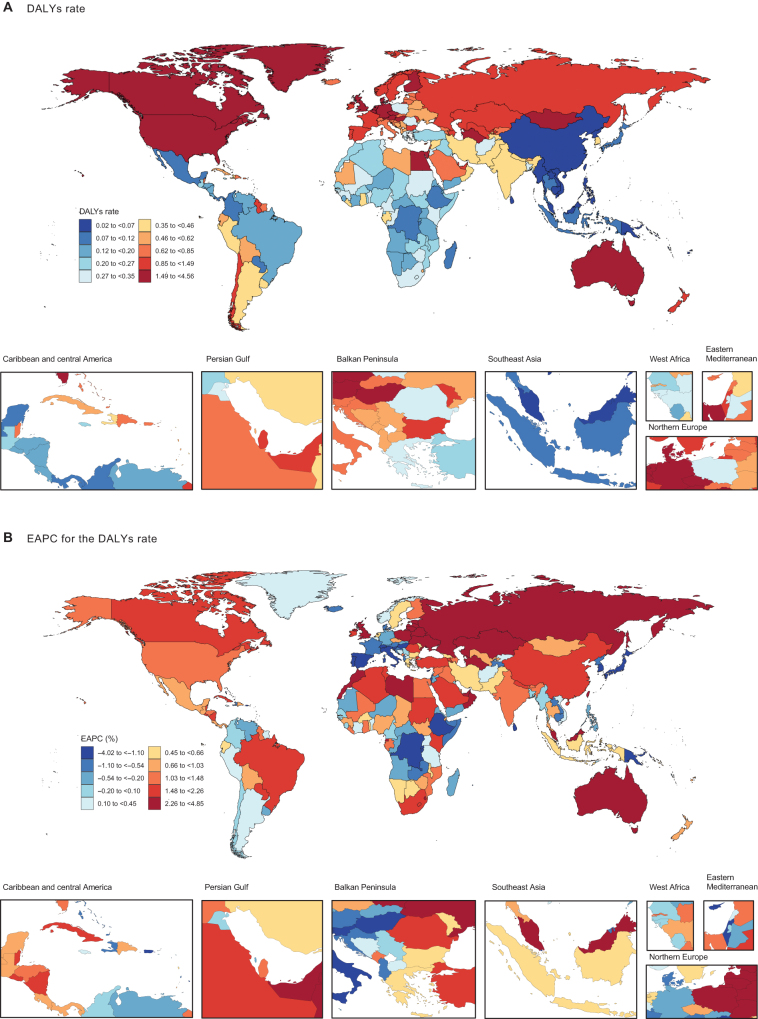
The age-standardized DALY (A) rate (per 10 million individuals) of MASLD–IBD comorbidity in 204 countries and territories in 2021; EAPC in the age-standardized DALY (B) rate of MASLD–IBD comorbidity in 204 countries and territories from 1990 to 2021. DALY, disability-adjusted life year; MASLD, metabolic dysfunction-associated steatotic liver disease; IBD, inflammatory bowel disease; EAPC, estimated annual percentage change.

In 2021, the age-standardized prevalence and DALYs rate of MASLD-IBD comorbidity were 171.7 cases (95% uncertainty interval [UI], 157.3–187.1) and 0.5 cases (95% UI, 0.4–0.6) per 10 million population. Among the 21 GBD regions, the highest age-standardized prevalence rates were observed in High-income North America (508.2; 95% UI, 464.3–552.1), Australasia (490.1; 95% UI, 448.6–535.8), and Western Europe (432.2; 95% UI, 396.2–470.5). In contrast, the lowest prevalence rates were found in Oceania (21.5; 95% UI, 19.8–23.5), Southeast Asia (22.7; 95% UI, 20.7–24.8), and Central Latin America (24.1; 95% UI, 22.0–26.2). Regarding age-standardized DALY rates, High-income North America (2.1; 95% UI, 1.7–2.7), Western Europe (1.8; 95% UI, 1.4–2.3), and Australasia (1.7; 95% UI, 1.4–2.1) exhibited the highest burden. For age-standardized mortality rates, High-income North America (7.9; 95% UI, 6.0–10.0), Western Europe (7.0; 95% UI, 5.3–8.8), and Australasia (6.6; 95% UI, 5.3–8.0) had the highest burden (Table 1). The age-standardized prevalence, DALYs, and mortality rates for MASLD-IBD comorbidity across 204 countries in 2021 are illustrated in Figures 1A and 2A, and also in Supplemental Digital Content Figure S5A and Supplemental Digital Content Table S4, available at: http://links.lww.com/JS9/F289. San Marino had the highest age-standardized prevalence rate (7.0; 95% UI, 5.3–8.8), while Canada had the highest age-standardized DALY rate (4.6; 95% UI, 3.5–5.7) and mortality rate (17.7; 95% UI, 13.8–22.3).

**Table 1:** Age-standardized prevalence and DALY rates (per 10 million individuals) for MASLD–IBD comorbidity in 1990 and 2021 and the EAPCs from 1990 to 2021, stratified by SDI and region

Location	Age-standardized prevalence rate			Age-standardized DALY rate			Age-standardized mortality rate		
	1990	2021	EAPC	1990	2021	EAPC	1990	2021	EAPC
	No. (95% UI)	No. (95% UI)	% (95% CI)	No. (95% UI)	No. (95% UI)	% (95% CI)	‱ (95% UI)	‱ (95% UI)	% (95% CI)
Global	147.8 (135.3, 161.3)	171.7 (157.3, 187.1)	0.60 (0.50, 0.71)	0.5 (0.4, 0.6)	0.5 (0.4, 0.6)	0.04 (−0.13, 0.21)	1.9 (1.4, 2.4)	1.9 (1.5, 2.3)	0.07 (−0.09, 0.23)
SDI									
Low SDI	65.4 (59.7, 71.5)	80.8 (73.8, 88.2)	0.76 (0.70, 0.83)	0.2 (0.2, 0.3)	0.2 (0.2, 0.3)	−0.01 (−0.07, 0.04)	1.0 (0.7, 1.4)	1.0 (0.8, 1.3)	0.09 (0.02, 0.15)
Low-middle SDI	96.8 (88.3, 105.7)	130.2 (119.2, 141.6)	1.15 (1.07, 1.22)	0.3 (0.2, 0.4)	0.4 (0.3, 0.5)	1.12 (1.02, 1.22)	1.2 (0.9, 1.7)	1.5 (1.1, 2.0)	1.03 (0.94, 1.12)
Middle SDI	49.4 (45.2, 54.0)	82.8 (75.7, 90.2)	1.96 (1.82, 2.11)	0.2 (0.1, 0.2)	0.2 (0.2, 0.3)	1.60 (1.46, 1.74)	0.6 (0.4, 0.8)	0.9 (0.7, 1.1)	1.71 (1.55, 1.88)
High-middle SDI	110.1 (100.9, 120.0)	124.7 (114.1, 136.0)	0.63 (0.43, 0.83)	0.3 (0.3, 0.4)	0.3 (0.2, 0.4)	−0.12 (−0.45, 0.21)	1.3 (1.0, 1.7)	1.1 (0.9, 1.4)	−0.29 (−0.57, −0.02)
High SDI	281.1 (257.7, 306.0)	390.4 (358.0, 423.7)	1.17 (0.99, 1.35)	1.3 (1.0, 1.6)	1.3 (1.0, 1.7)	0.10 (−0.15, 0.35)	4.7 (3.6, 6.1)	5.0 (3.9, 6.2)	0.21 (−0.03, 0.45)
Regions									
High-income Asia Pacific	58.8 (53.7, 64.0)	84.4 (77.4, 91.9)	1.49 (1.02, 1.96)	0.3 (0.2, 0.4)	0.2 (0.1, 0.2)	−1.66 (−2.23, −1.08)	1.2 (0.9, 1.5)	0.8 (0.6, 1.0)	−1.21 (−1.79, −0.62)
Central Asia	166.1 (152.5, 181.4)	187.2 (171.1, 204.5)	0.49 (0.39, 0.59)	0.7 (0.5, 0.9)	1.1 (0.8, 1.5)	1.63 (1.40, 1.86)	2.6 (1.9, 3.4)	4.0 (2.9, 5.3)	1.70 (1.47, 1.93)
East Asia	18.3 (16.7, 20.1)	36.1 (33.0, 39.3)	3.18 (2.59, 3.77)	0.0 (0.0, 0.0)	0.0 (0.0, 0.1)	1.54 (0.96, 2.13)	0.1 (0.1, 0.2)	0.2 (0.2, 0.2)	1.94 (1.31, 2.56)
South Asia	122.4 (111.7, 134.0)	166.6 (152.3, 181.7)	1.16 (1.05, 1.27)	0.3 (0.2, 0.4)	0.4 (0.3, 0.5)	1.01 (0.91, 1.11)	1.1 (0.8, 1.6)	1.5 (1.1, 2.0)	1.24 (1.13, 1.34)
Southeast Asia	19.8 (18.1, 21.7)	22.7 (20.7, 24.8)	0.56 (0.51, 0.60)	0.1 (0.0, 0.1)	0.1 (0.0, 0.1)	0.44 (0.37, 0.52)	0.2 (0.2, 0.3)	0.3 (0.2, 0.3)	0.63 (0.55, 0.71)
Australasia	360.7 (329.5, 392.4)	490.1 (448.6, 535.8)	1.40 (1.09, 1.70)	1.1 (0.8, 1.4)	1.7 (1.4, 2.1)	2.20 (1.87, 2.52)	3.8 (2.9, 4.9)	6.6 (5.3, 8.0)	2.34 (2.04, 2.65)
Caribbean	100.0 (91.4, 109.1)	109.7 (100.5, 119.0)	0.39 (0.34, 0.44)	0.5 (0.4, 0.7)	0.6 (0.4, 0.8)	0.26 (−0.05, 0.58)	2.0 (1.4, 2.6)	2.1 (1.5, 2.9)	0.13 (−0.16, 0.43)
Central Europe	190.5 (174.5, 207.1)	217.6 (198.6, 236.7)	0.60 (0.43, 0.77)	0.6 (0.5, 0.9)	0.8 (0.6, 1.1)	0.53 (0.28, 0.79)	2.3 (1.7, 3.0)	2.9 (2.1, 3.9)	0.56 (0.34, 0.78)
Eastern Europe	104.4 (95.4, 113.7)	110.8 (101.4, 120.4)	0.29 (0.10, 0.48)	0.2 (0.2, 0.3)	0.8 (0.6, 1.1)	4.05 (3.32, 4.79)	0.9 (0.6, 1.1)	2.4 (1.7, 3.3)	3.55 (2.98, 4.12)
Western Europe	296.7 (272.8, 323.2)	432.2 (396.2, 470.5)	1.16 (0.92, 1.40)	2.1 (1.6, 2.8)	1.8 (1.4, 2.3)	−0.71 (−1.06, −0.36)	7.9 (5.8, 10.2)	7.0 (5.3, 8.8)	−0.57 (−0.89, −0.24)
Andean Latin America	43.4 (39.8, 47.4)	52.1 (47.7, 56.9)	0.61 (0.51, 0.71)	0.4 (0.3, 0.6)	0.5 (0.3, 0.7)	0.44 (0.25, 0.63)	1.6 (1.1, 2.2)	2.0 (1.4, 2.8)	0.80 (0.64, 0.96)
Central Latin America	21.2 (19.4, 23.1)	24.1 (22.0, 26.2)	0.53 (0.43, 0.63)	0.2 (0.1, 0.2)	0.2 (0.1, 0.3)	0.63 (0.46, 0.80)	0.6 (0.4, 0.8)	0.7 (0.5, 0.9)	0.65 (0.48, 0.81)
Southern Latin America	99.7 (91.3, 109.5)	137.4 (125.4, 150.4)	1.08 (1.03, 1.14)	0.6 (0.4, 0.9)	0.6 (0.4, 0.8)	0.32 (0.18, 0.46)	2.2 (1.5, 3.0)	2.3 (1.6, 3.1)	0.50 (0.37, 0.63)
Tropical Latin America	48.9 (44.9, 53.5)	86.5 (79.1, 94.5)	1.53 (1.04, 2.02)	0.1 (0.1, 0.2)	0.2 (0.1, 0.3)	1.67 (1.17, 2.18)	0.4 (0.3, 0.5)	0.7 (0.5, 0.9)	1.91 (1.41, 2.41)
North Africa and Middle East	162.7 (149.3, 177.2)	251.6 (232.5, 271.8)	1.83 (1.55, 2.10)	0.4 (0.3, 0.6)	0.5 (0.4, 0.7)	1.36 (1.14, 1.58)	2.0 (1.3, 3.0)	2.5 (1.8, 3.3)	1.10 (0.91, 1.29)
High-income North America	411.6 (375.7, 448.6)	508.2 (464.3, 552.1)	0.87 (0.68, 1.05)	1.6 (1.2, 2.1)	2.1 (1.7, 2.7)	1.34 (1.19, 1.48)	5.6 (4.2, 7.3)	7.9 (6.1, 10.0)	1.42 (1.28, 1.56)
Oceania	20.3 (18.5, 22.2)	21.5 (19.8, 23.5)	0.17 (0.11, 0.23)	0.0 (0.0, 0.1)	0.0 (0.0, 0.0)	−0.78 (−0.92, −0.64)	0.1 (0.1, 0.2)	0.1 (0.1, 0.2)	−0.71 (−0.85, −0.57)
Central Sub-Saharan Africa	36.7 (33.5, 40.4)	37.9 (34.6, 41.3)	−0.05 (−0.26, 0.16)	0.2 (0.1, 0.2)	0.1 (0.1, 0.2)	−0.76 (−1.03, −0.50)	0.6 (0.4, 1.0)	0.5 (0.3, 0.8)	−0.74 (−1.00, −0.48)
Eastern Sub-Saharan Africa	33.9 (30.9, 37.1)	43.2 (39.5, 47.2)	0.60 (0.52, 0.69)	0.2 (0.1, 0.2)	0.2 (0.1, 0.3)	0.19 (0.07, 0.32)	0.7 (0.5, 0.9)	0.8 (0.6, 1.0)	0.29 (0.18, 0.41)
Southern Sub-Saharan Africa	51.2 (46.7, 55.8)	70.1 (64.1, 76.5)	0.87 (0.81, 0.92)	0.2 (0.1, 0.3)	0.3 (0.3, 0.4)	1.51 (0.96, 2.07)	0.7 (0.5, 1.0)	1.2 (1.0, 1.5)	1.60 (1.08, 2.12)
Western Sub-Saharan Africa	43.4 (39.6, 47.4)	60.3 (55.1, 66.0)	1.11 (1.05, 1.17)	0.2 (0.1, 0.3)	0.3 (0.2, 0.3)	0.65 (0.60, 0.70)	0.9 (0.6, 1.4)	1.1 (0.9, 1.5)	0.71 (0.66, 0.76)

DALY, disability adjusted life year; EAPC, estimated annual percentage change; IBD, inflammatory bowel disease; MASLD, metabolic dysfunction-associated steatotic liver disease; SDI, socio-demographic index.

In 2021, the total prevalent cases of MASLD-IBD comorbidity globally were 156 776.4 (95% UI, 143 182.6–170 695.7), with 45 347.1 DALYs (95% UI, 35 903.7–56 971.0) attributable to the condition (Supplemental Digital Content Table S5, available at: http://links.lww.com/JS9/F289). Among the 21 regions, Western Europe had the highest number of prevalent cases (35 867.3; 95% UI, 32 988.1–38 737.8) and the most significant DALYs burden (18 930.2; 95% UI, 14 606.8–23 918.4) (Supplemental Digital Content Tables S5–S7, available at: http://links.lww.com/JS9/F289) further detail the distribution of prevalent cases, DALYs, and deaths attributable to MASLD-IBD comorbidity across 204 countries. The United States of America had the highest number of prevalent cases (28 489.1; 95% UI, 26 004.0–31 074.2), DALYs (13 232.9; 95% UI, 10 283.7–16 997.9), and deaths (531.2; 95% UI, 410.2–671.2) (Supplemental Digital Content Figures S3A, S4A, S5A, and S6A, available at: http://links.lww.com/JS9/F289.

**Figure 3.**
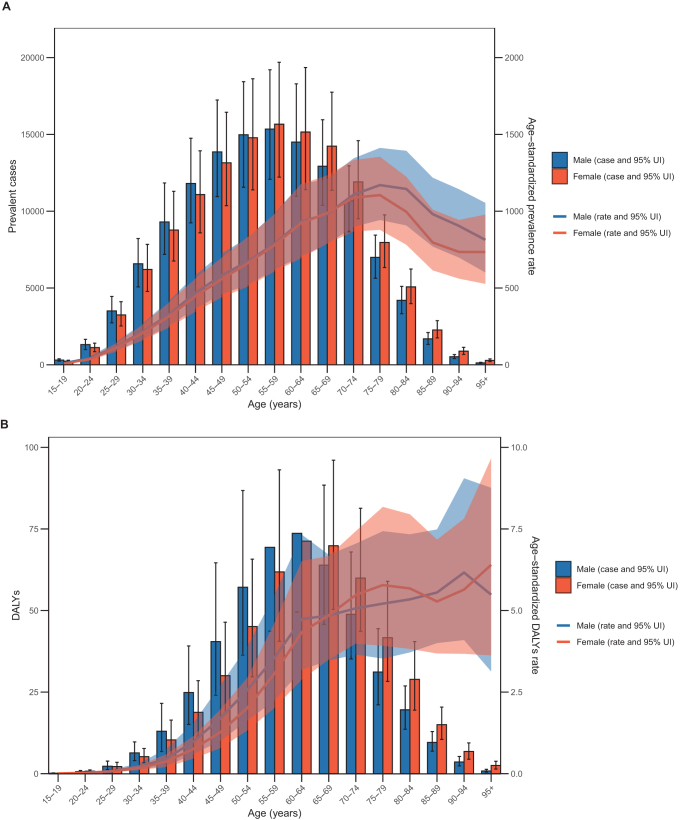
The global numbers and age-standardized rate (per 10 000,000 individuals) for the prevalence (A) and DALYs (B) of MASLD–IBD comorbidity in 2021, stratified by age and sex. DALY, disability adjusted life year; MASLD, metabolic dysfunction-associated steatotic liver disease; IBD, inflammatory bowel disease.

Additionally, Supplemental Digital Content Tables S8 and S9, available at: http://links.lww.com/JS9/F289 present the age-standardized prevalence, DALYs, and mortality rates stratified by region for men and women, highlighting gender disparities in the disease burden. Notably, among age-standardized prevalent cases, men under 55 had higher numbers than women, while women over 55 surpassed men. For prevalence rates, men and women under 70 years were nearly equal, but men over 70 years had significantly higher rates. DALY burden was higher in men under 65 years, while women over 65 exceeded men. Similarly, DALY rates were slightly higher in men under 70 years compared to women (Fig. 3A and B).

When stratified by SDI, high SDI regions had the highest age-standardized prevalence (390.4; 95% UI, 358.0–423.7), DALY rate (1.3; 95% UI, 1.0–1.7), and mortality rate (5.0; 95% UI, 3.9–6.2) (Table 1 and Supplemental Digital Content Table S6, available at: http://links.lww.com/JS9/F289). Middle SDI regions had the highest numbers of prevalent cases, DALYs, and deaths (Supplemental Digital Content Table S5, available at: http://links.lww.com/JS9/F289).

Notably, the disease burden of comorbidity in the elderly was significantly higher than in the general population, approximately six times greater (Supplemental Digital Content Table S9, available at: http://links.lww.com/JS9/F289). The study revealed that the age-standardized prevalence of comorbidity peaked in the 75–79 age-group, while prevalent cases peaked in the 55–59 age-group (Fig. 3A). Furthermore, DALYs and mortality rates showed a continuous upward trend with increasing age (Fig. 3B and Supplemental Digital Content Figure S7, available at: http://links.lww.com/JS9/F289). Consistent with these findings, in high SDI regions, adults aged 60 and above had significantly higher age-standardized prevalence, DALYs, and mortality rates attributable to comorbidity compared to other age-groups (Supplemental Digital Content Table S9, available at: http://links.lww.com/JS9/F289).

### Trends of global burden of MASLD-IBD comorbidity from 1990 to 2021

**Figure 4.**
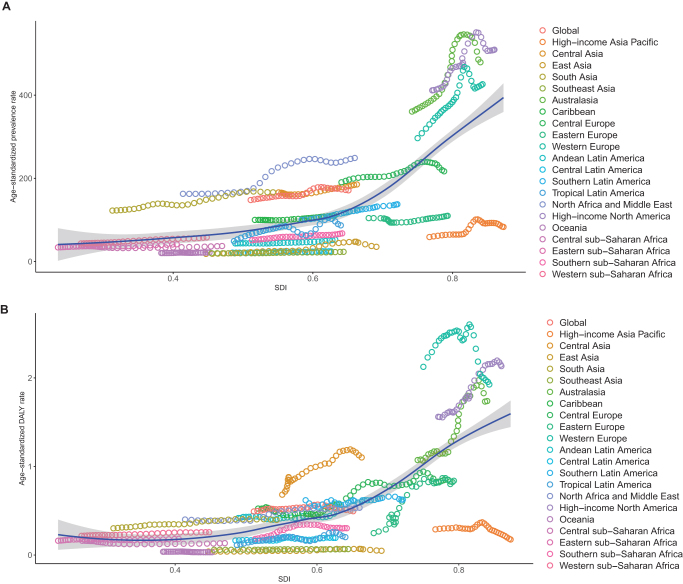
The age-standardized prevalence (A) and DALY (B) rate (per 10 million individuals) of MASLD–IBD comorbidity in the 21 GBD regions by SDI during 1990–2021. Each dot represents the disease burden for a year in that region. The blue line, a locally weighted scatterplot smoothing smoother, presents the expected global values based on the SDI values. DALY = disability adjusted life year. MASLD, metabolic dysfunction-associated steatotic liver disease; IBD, inflammatory bowel disease; GBD, global disease of burden; SDI, socio-demographic index.

Based on data analysis from 21 regions from 1990 to 2021 by the GBD study, the disease burden of MASLD-IBD comorbidity showed an overall upward trend and was positively correlated with the SDI. The global burden was slightly higher than expected, with regions such as High-income North America, Australasia, and Western Europe bearing a higher burden than anticipated, while High-income Asia Pacific, Eastern Europe, and East Asia had a lower burden than expected (Fig. 4A and B, and Supplemental Digital Content Figure S9, available at: http://links.lww.com/JS9/F289). In terms of the evolution of age-standardized prevalence rates, East Asia led globally with an annual growth rate of 3.18% (95%, CI, 2.59–3.77), significantly higher than North Africa and the Middle East (EAPC = 1.83; 95% CI, 1.55–2.10) and Tropical Latin America (EAPC = 1.53; 95% CI, 1.04–2.02). The only region showing a declining trend was Central Sub-Saharan Africa (EAPC = −0.05; 95% CI, −0.26 to 0.16), although the decrease was insignificant. Analysis of DALY rates revealed more profound disparities in disease impact, with 17 regions showing an upward trend. Eastern Europe had the fastest increase in DALYs burden, with an annual growth rate of 4.05% (95%, CI, 3.32–4.79), followed by Australasia (EAPC = 2.20; 95% CI, 1.87–2.52) and Tropical Latin America (EAPC = 1.67; 95% CI, 1.17–2.18). Conversely, the regions with the most significant declines in DALY rates included high-income Asia Pacific (EAPC = −1.21; 95% CI, −1.79 to −0.62), Central Sub-Saharan Africa (EAPC = −0.74; 95% CI, −1.00 to −0.48), and Oceania (EAPC = −0.71; 95% CI, −0.85 to −0.57). The geographic distribution of age-standardized mortality rates further highlighted regional disparities in disease management, with the highest annual increases observed in Eastern Europe (EAPC = 3.55; 95% CI, 2.98–4.12), Australasia (EAPC = 2.34; 95% CI, 2.04–2.65), and East Asia (EAPC = 1.94; 95% CI, 1.31–2.56), while the lowest increases were seen in High-income Asia Pacific (EAPC = −1.21; 95% CI, −1.79 to −0.62), Central Sub-Saharan Africa (EAPC = −0.74; 95% CI, −1.00 to −0.48), and Australasia (EAPC = −0.71; 95% CI, −0.85 to −0.57) (Table 1 and Supplemental Digital Content Figure S8, available at: http://links.lww.com/JS9/F289).

Among 204 countries, 201 (98.53%) showed an increase in age-standardized prevalence rates, with the highest annual growth rates observed in Brunei Darussalam (EAPC = 3.23; 95% CI, 2.64–3.83), Liberia (EAPC = 2.95; 95% CI, 2.17–3.74), and the United Republic of Tanzania (EAPC = 2.54; 95% CI, 2.08–3.01). Only Seychelles, Zimbabwe, and Equatorial Guinea exhibited declining trends. Regarding age-standardized DALY rates, 133 countries (65.20%) showed an upward trend. The countries with the highest annual growth rates included the Russian Federation (EAPC = 4.85; 95% CI, 4.09–5.62), Lithuania (EAPC = 4.39; 95% CI, 2.90–5.90), and Belarus (EAPC = 3.93; 95% CI, 2.98–4.90), while 71 countries experienced a decline in age-standardized DALY rates. The most significant declines in DALY rates were observed in Italy (EAPC = −4.02; 95% CI, −4.19 to −3.84), Hungary (EAPC = −3.29; 95% CI, − 3.91 to −2.67), and Japan (EAPC = −2.53; 95% CI, − 3.18 to −1.87) (Table 1, Figures 1B and 2B). The annual increases in age-standardized mortality rates followed a similar trend to DALY rates (Supplemental Digital Content Figure S5B, available at: http://links.lww.com/JS9/F289).

From 1990 to 2019, the global number of prevalent cases, DALYs, and deaths attributable to MASLD-IBD comorbidity increased by 115.18% (95% CI, 123.22–188.85), 149.77% (95% CI, 71.38–255.43), and 0.17% (95% CI, 0.06–0.28), respectively (Supplemental Digital Content Table S5, available at: http://links.lww.com/JS9/F289). Supplemental Digital Content Table S5 and Figures S3B and S4B, available at: http://links.lww.com/JS9/F289 illustrate the changes in prevalent cases and DALYs across 21 regions and 204 countries from 1990 to 2021. Tropical Latin America had the highest relative changes in prevalent cases (EAPC = 402.86; 95% CI, 338.50–470.85) and DALYs (EAPC = 468.80; 95% CI, 268.18–752.45). The trends in age-standardized mortality rates and the number of death cases from 1990 to 2021 were similar to those of DALYs, as shown in Supplemental Digital Content Table S6, available at: http://links.lww.com/JS9/F289, Supplemental Digital Content Figures S5B, S6B, and S9, available at: http://links.lww.com/JS9/F289.

**Figure 5.**
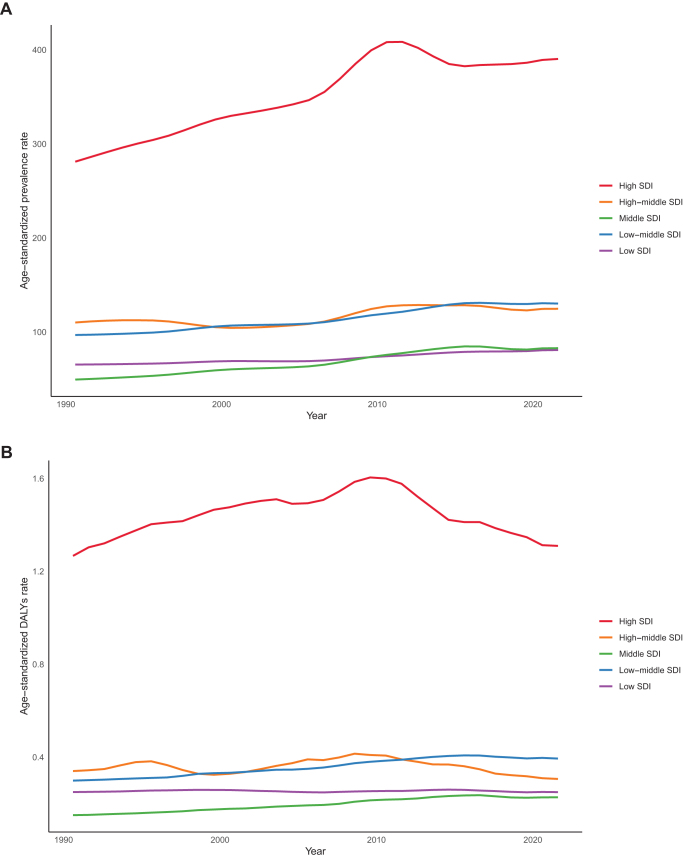
The trends in the age-standardized prevalence (A) and DALY (B) rate (per 10 million individuals) of MASLD–IBD comorbidity from 1990 to 2021, stratified by the SDI level. DALY, disability adjusted life year; MASLD, metabolic dysfunction-associated steatotic liver disease; IBD, inflammatory bowel disease; SDI, socio-demographic index.

Significant heterogeneity was observed in disease burden metrics across different SDI regions. High SDI regions exhibited higher levels of age-standardized prevalence, DALY rates, and mortality rates. Notably, DALY rates in high SDI regions showed an upward trend from 1990 to 2010, followed by a decline after 2010, but remained high overall (Figs 5A and B). Importantly, middle SDI regions demonstrated the most prominent growth in disease burden. From 1990 to 2021, middle SDI regions had the highest annual growth rates in age-standardized prevalence (EAPC = 1.96; 95% CI, 1.82–2.11), DALY rates (EAPC = 1.60; 95% CI, 1.46–1.74), and mortality rates (EAPC = 1.71; 95% CI, 1.55–1.88) among all SDI groups (Table 1 and Supplemental Digital Content Table S6, available at: http://links.lww.com/JS9/F289). The absolute number of cases also increased significantly: prevalent cases rose by 333.80% (95% CI, 279.13–391.47), DALYs by 369.24% (95% CI, 225.62–558.95), and deaths by 428.41% (95% CI, 225.62–558.95) (Supplemental Digital Content Table S5 and Supplemental Digital Content Figure S10, available at: http://links.lww.com/JS9/F289).

### Predicted global patterns and trends in MASLD-IBD comorbidity burden from 2021 to 2050

**Figure 6.**
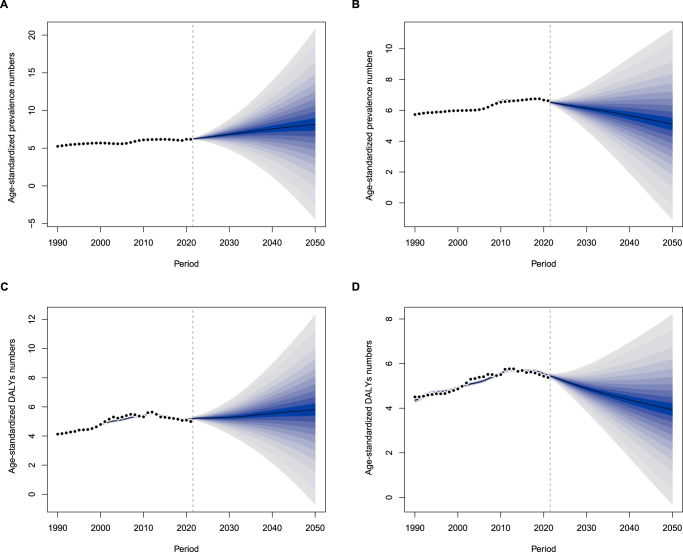
Global predictions of MASLD-IBD comorbidity related age-standardized prevalence, DALYs, and mortality numbers (per 10 million individuals) from 2021 to 2050. (A). The age-standardized prevalence numbers among the female population. (B) The age-standardized prevalence numbers among the male population. (C) The age-standardized DALY numbers among the female population. (D) The age-standardized DALY numbers among the male population. MASLD, metabolic dysfunction-associated steatotic liver disease; IBD, inflammatory bowel disease; DALY, disability adjusted life year.

We employed a Bayesian age-period-cohort model to project the prevalent cases and DALYs of MASLD-IBD comorbidity by gender from 2021 to 2050. The projected results indicate that by 2050, the prevalent cases and DALYs among women will continue to rise, whereas men will transition from an upward trend observed between 1990 and 2021 to a sustained decline. Furthermore, over the next three decades, the prevalent cases and DALYs in women are expected to remain higher than in men (Fig. 6). Additionally, the prevalent rates for both men and women show a declining trend, suggesting a gradual reduction in the magnitude of change (Supplemental Digital Content Figure S11, available at: http://links.lww.com/JS9/F289).

## Discussion

Previous studies have shown that IBD increases the risk of MASLD in patients^[26]^. Pathogenesis is primarily related to intestinal barrier dysfunction in patients with IBD^[40,41]^, leading to intestinal microbes and their metabolites (such as endotoxins) entering the bloodstream and triggering systemic inflammatory responses^[42]^. These inflammatory factors and microbial products not only exacerbate intestinal inflammation but also directly affect the liver through the portal vein system, intensifying liver inflammation and fat deposition, thereby promoting the occurrence and development of MASLD^[43,44]^.

Epidemiological data revealed that the age-standardized prevalence and DALY rates of MASLD-IBD comorbidity have been increasing in most GBD regions (20 out of 21) over the past three decades. Regions with faster socio-economic development, such as East Asia, have seen a higher increase in MASLD-IBD comorbidity^[45]^. In 2021, high-income regions like North America, Australasia, and Western Europe had the highest disease burden, which may be closely related to higher obesity rates and metabolic syndrome prevalence in these areas^[46]^. This trend suggests that MASLD-IBD comorbidity may become a significant public health challenge in the future. However, the only region where the age-standardized prevalence and DALY rates have shown a declining trend is Central Sub-Saharan Africa. This decline may be attributed to significant improvements in local healthcare infrastructure and measures to reduce metabolic syndrome^[47]^.

The disease burden of MASLD-IBD comorbidities exhibits significant SDI gradients. High SDI regions bear the largest and continuously increasing disease burden, with a consistently high prevalence rate^[4,48–50]^. Notably, although the MASLD disease burden in high SDI regions is significantly lower than that in middle and low SDI regions^[51,52]^, controlling IBD in patients with MASLD in high SDI regions has become a significant public health issue due to the synergistic effects between MASLD and IBD. Additionally, the disease burden growth rate is highest in middle SDI regions, which may be closely related to lifestyle changes (high-calorie diets, antibiotic use)^[53]^, population aging^[54]^, improved medical resources^[55]^, epidemiological transitions, and other factors. These findings emphasize the need for early lifestyle interventions to reduce the risk of IBD in patients with MASLD in both high and middle SDI regions and strengthen treatment strategies for MASLD.

In addition to regional differences, the disease burden of MASLD-IBD comorbidity shows gender differences, particularly prominent in middle-aged and elderly populations. A systematic review and meta-analysis showed that the prevalence of MASLD in male patients with IBD is 28.0% (95% CI, 14.5−44.0; I2 = 99.5), while in female patients it is 22.5% (95% CI, 11.6−35.9; I2 = 99.4), indicating a slightly higher prevalence in male patients^[45]^. Another study found that the risk of MASLD and IBD is more significant in women aged ≥60 years^[31]^, which may be related to the dysregulation of estrogen receptor β subtypes, leading to increased intestinal permeability, reduced estrogen-mediated immune protection, and dysbiosis of the gut microbiota^[56–59]^. According to projections from 2021 to 2025, the disease burden among women is expected to continue rising, whereas that among men is predicted to decline steadily. Therefore, interventions should be explicitly implemented for different genders and age-groups to prevent the development of MASLD-IBD comorbidity. Further research indicates that the burden of MASLD-IBD comorbidity in individuals aged 60 and above is significantly higher than that in the general population^[20,31,60]^, potentially related to metabolic syndrome, obesity, chronic inflammatory states, and reduced diversity of gut microbiota^[61]^. Given the rapid aging trend among patients with patients, our findings highlight the importance of regular screening and effective treatment of IBD in elderly patients with MASLD.

The correlation between MASLD and IBD with regions, age, and gender may be influenced by various factors. Lifestyle factors (such as high-fat, high-sugar, low-fiber diets, high alcohol intake^[53]^, and chronic stress^[62]^) and environmental factors (such as air pollution^[63]^) may further exacerbate the risk of both diseases by affecting intestinal barrier function and the immune system^[64]^. Additionally, the widespread presence of chronic inflammatory states also negatively impacts liver and intestinal health^[65]^, collectively driving the rapid increase in the prevalence of MASLD and IBD.

Treatment for MASLD-IBD comorbidity requires a comprehensive intervention strategy to reduce inflammatory responses and repair intestinal barrier function. In terms of diet, a diet rich in vegetables, fruits, whole grains, and olive oil is recommended^[66]^, avoiding high-fat, high-sugar, low-fiber diets; in terms of lifestyle, weight control, reduced alcohol intake, stress management, and regular sleep are necessary^[67]^; in terms of medication, patients with IBD can use anti-inflammatory drugs (such as 5-aminosalicylic acid, 5-ASA, or biologics^[68–70]^), and patients with MASLD can use metabolic improvement drugs (such as GLP-1 receptor agonists or PPAR-γ agonists^[68–71]^). Moreover, supplementing probiotics and prebiotics can synergistically treat MASLD and IBD by regulating the gut microbiota, enhancing intestinal barrier function, and reducing inflammatory responses^[72]^. These comprehensive interventions help improve the composition of gut microbiota, enhance intestinal barrier function, and reduce inflammatory responses, but also break the pathological cycle between MASLD and IBD^73^, providing synergistic protection for both diseases and improving long-term patient outcomes.

This study has several limitations. First, the assumption that RR and comorbidity prevalence (*p*) remain constant across time, age, sex, and region introduces significant uncertainty into disease burden estimates. This assumption stemmed from limited data availability; stratified RR and *p* values were unavailable. This reflects a broader field challenge rather than a specific study design flaw. To systematically evaluate and quantify the uncertainty introduced by these parameters, we conducted a Monte Carlo sensitivity analysis (detailed simulation results are presented in Supplemental Digital Content Table S10 and Figures S12–S15, available at: http://links.lww.com/JS9/F289).

Second, the reliability of findings depends critically on data quality and coverage within the GBD 2021 database. In low-income regions, limited healthcare access causes widespread underdiagnosis and misdiagnosis of MASLD, creating substantial data deficiencies. These gaps introduce population representation bias, systematically underestimating health characteristics in vulnerable groups. They also generate uncorrectable systemic errors, ultimately compromising the accuracy and generalizability of conclusions. Within global burden assessments specifically, such data limitations likely underestimate the true MASLD burden, potentially leading to inadequate health policy formulation and resource allocation. Future research should incorporate alternative data sources, including community health surveys and mobile health data, alongside advanced modeling techniques like Bayesian or hierarchical models to correct deficiencies and reduce bias.

Third, this analysis was limited to describing the disease burden associated with MASLD-related comorbidities. It did not assess the efficacy of preventive strategies like early screening or lifestyle interventions, nor their potential cost-effectiveness. Consequently, the findings offer limited direct applicability for public health policy. Future research should employ modeling approaches, including decision trees, Markov, or microsimulation models, to quantify the impact and economic benefits of interventions preventing MASLD-IBD comorbidities. This will generate more comprehensive evidence for evidence-based decision-making.

Fourth, due to the lack of individual-level data in the GBD 2021 database, we were unable to account for potential confounding factors (such as genetic background, obesity, medication use, and lifestyle), which may affect the reliability of causal inferences. Future studies should incorporate large-scale prospective cohort studies to collect baseline data and combine individual-level data analysis to systematically evaluate the influence of these confounders. Additionally, Mendelian randomization and other methods could be further employed to more accurately assess the causal relationship between MASLD comorbidities, their risk factors, and the burden of liver cancer, thereby providing further validation and refinement of the current findings.

## Conclusion

This comprehensive analysis characterizes the global burden and spatiotemporal evolution of MASLD-IBD comorbidity spanning 1990–2021, with model-based projections delineating long-term trajectories extending to 2050. Findings reveal marked regional disparities in the disease burden of MASLD-IBD comorbidity, with the most pronounced increases observed in middle- and high-SDI regions over three decades. Against the backdrop of rising IBD prevalence and an aging MASLD population, these trends highlight the escalating threat posed by MASLD-IBD comorbidity. The study underscores the urgent need for integrated clinical and public health strategies that concurrently address MASLD-IBD comorbidity to mitigate this dual disease burden in high-risk populations.

## Supplementary Material

## Data Availability

Data are available in a public, open-access repository. Data from the GBD study in 2021, which provides aggregated and anonymized population-level estimates, can be accessed using the GBD Results Tool (https://vizhub.healthdata.org/gbd-results/), maintained by the Institute for Health Metrics and Evaluation. The GBD data are licensed for non-commercial use and do not contain identifiable individual information.
