# Supplementary material for: Global burden of MASLD-IBD comorbidity from 1990 to 2021 and trend prediction to 2050
Source: Int J Surg. 2025 Oct 13;112(1):1528–42. doi: 10.1097/JS9.0000000000003383 (PMC12825745; doi:10.1097/JS9.0000000000003383)
Supplement: Supplementary file 1 [file js9-112-1528-001.docx]

Supplementary Appendix

**Global burden of MASLD-IBD comorbidity from 1990 to 2021**

**and trend prediction to 2050**

# Supplementary Methods

## The definitions for metabolic dysfunction-associated fatty liver disease (MASLD) infection and inflammatory bowel disease (IBD) in this study.

### MASLD

Metabolic Dysfunction-Associated Steatotic Liver Disease (MASLD), introduced in June 2023 through a Delphi consensus statement by the American Association for the Study of Liver Diseases (AASLD) and multinational hepatology societies, replaces the previous term "non-alcoholic fatty liver disease (NAFLD)" to emphasize its metabolic pathogenesis[1, 2]. MASLD is defined as a metabolic disorder characterized by hepatic fat content exceeding 5% of liver weight, after excluding excessive alcohol consumption (men <30 g/day, women <20 g/day) and secondary hepatic disorders[3, 4]. The disease spectrum comprises two main subtypes: metabolic dysfunction-associated steatotic liver and metabolic dysfunction-associated steatohepatitis. Metabolic dysfunction-associated steatotic liver manifests as simple hepatic steatosis, while metabolic dysfunction-associated steatohepatitis involves lipid accumulation accompanied by inflammatory infiltration, hepatocellular injury, and fibrosis, which may progress to cirrhosis or hepatocellular carcinoma[5]. With a global prevalence exceeding 25%, MASLD pathogenesis is closely linked to insulin resistance, metabolic syndrome, and gene-environment interactions, making it a critical risk factor for chronic liver and cardiovascular diseases[3, 6].

### IBD

Inflammatory bowel disease (IBD) is a group of chronic, non-specific inflammatory diseases of the intestine with unknown etiology. It is characterized by a course of alternating relapses and remissions, transmural or mucosal inflammatory damage, and a risk of disabling complications[7, 8]. According to the extent of involvement and pathological features, IBD is mainly divided into Crohn's disease, ulcerative colitis, and unclassified colitis. The core pathogenic mechanisms involve genetic susceptibility (such as mutations in NOD2 and IL23R genes), dysbiosis of the gut microbiota, mucosal immune dysfunction, and the interaction of environmental factors (such as industrialized diet and smoking), posing a significant public health challenge across regions[9, 10].

### Disease coding

MASLD is identified using ICD-10 codes K75.8 (Other specified inflammatory liver diseases) and K76.0 (Fatty liver), consistent with the latest MASLD nomenclature consensus [11, 12]. IBD encompasses both Ulcerative Colitis (ICD-10 K51) and Crohn's Disease (ICD-10 K50) [13, 14]. It is crucial and clearly stated that the GBD database does not treat MASLD-IBD comorbidity as an independent disease entity. To rigorously avoid double-counting and accurately quantify the burden, the study employs the Population Attributable Fraction (PAF) method. This involves extracting the total MASLD burden (including prevalence, DALYs, and mortality) from the GBD database and then calculating the burden attributable to IBD using the formula: Attributable Burden = Total MASLD Burden × PAF[15, 16]. This approach, leveraging a relative risk of 1.13 for developing hepatitis in MASLD-IBD patients compared to MASLD alone, provides a statistically sound and transparent method for assessing the contribution of IBD to MASLD, thereby ensuring the research's accuracy, reliability, and feasibility.

### Comorbidity statistics method

The GBD database does not classify MASLD-IBD comorbidity as an independent disease entity. To effectively avoid double-counting, this study employed the PAF method to quantify the contribution of IBD to the burden of MASLD. Specifically, we first extracted the overall burden data of MASLD from the GBD database, including prevalence, disability-adjusted life years (DALYs), and mortality. Subsequently, the IBD-attributable burden was calculated using the formula: Attributable Burden = Total MASLD Burden × PAF [15, 16]. Here, PAF was computed based on Levin's formula (Equation 1), leveraging the relative risk (RR = 1.13) of MASLD in IBD patients and the comorbidity prevalence (*p* = 1.96). This approach ensures exclusive quantification of the excess MASLD burden attributable to IBD, thereby preventing overlap with either the baseline MASLD burden or the standalone IBD burden.


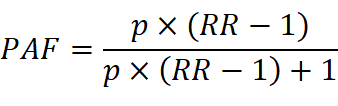


Eq. 1

## The search strategy for the GBD 2021 database

**Table S1.** Search strategy for the GBD 2021 database

| Variable | Search strategy |
| --- | --- |
| GBD Estimate | Cause of death or injury |
| Measure | Prevalence; Disability-Adjusted Life Years (DALYs); Deaths |
| Metric | Number; Rate |
| Cause | Non-alcoholic fatty liver disease; Inflammatory bowel disease |
| Location | Global; All GBD regions; All countries and territories; Low SDI; Low middle SDI; Middle SDI; High-middle SDI; High SDI |
| Ages | All ages; Age-standardized; 15-19 years; 20-24 years; 25-29 years; 30- 34 years; 35-39 years; 40-44 years; 45-49 years; 50-54 years; 55-59 years; 60-64 years; 65-69 years; 70-74 years; 75-79 years; 80-84 years; 85-89 years; 90-94 years; 95+ years |
| Sex | Both; Male; Female |
| Year | 1990; 1991; 1992; 1993; 1994; 1995; 1996; 1997; 1998; 1999; 2000; 2001; 2002; 2003; 2004; 2005; 2006; 2007; 2008; 2009; 2010; 2011; 2012; 2013; 2014; 2015; 2016; 2017; 2018; 2019; 2020; 2021 |

GBD, global burden of disease; SDI, sociodemographic index.

## The search strategy to estimate the relative risk (RR) in Equation 1

We conducted a systematic search in the Web of Science database using the terms "(Non-alcoholic Fatty Liver Disease OR Fatty Liver, Nonalcoholic OR Nonalcoholic Fatty Liver Disease OR Nonalcoholic Steatohepatitis OR NAFLD OR Metabolic Dysfunction-Associated Steatotic Liver Disease OR MASLD) AND (Bowel Diseases, Inflammatory OR Inflammatory Bowel Diseases OR IBD) AND (meta-analysis OR cohort study)" to identify studies published between January 1, 2020, and February 5, 2025, that examined RR of risk factors associated with IBD contributing to the development of MASLD. Following the initial search and screening process, a total of 24 relevant articles were identified. Among these, only one study provided an estimated RR as described in Equation 1[17].


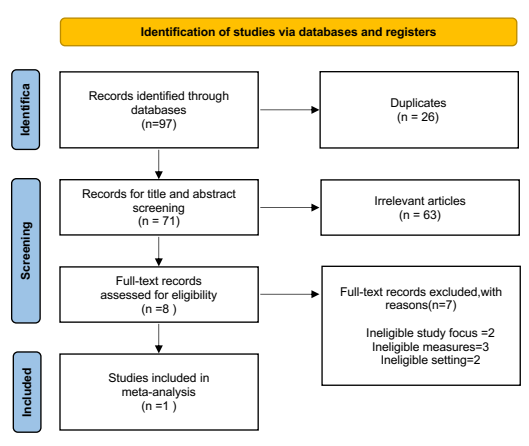


**Figure S1.** PRISMA flow diagram illustrating the literature screening process for the systematic review. A total of 97 articles were identified from the Web of Science. After duplicate removal, 71 articles remained. Irrelevant articles (*n*=63) were excluded during title/abstract screening. Full-text review excluded 2 articles due to ineligible study focus, 3 articles due to outcome measure inconsistency, and 2 articles due to population/setting discrepancy. One article was ultimately included for RR estimation. Arrows indicate the direction of the screening process, with boxed numbers representing the quantity of articles retained at each stage.

## Impact of Parameter Uncertainty on Burden Estimates

To assess the impact of uncertainty in the RR and *p*-value (prevalence of IBD in patients with MASLD) on burden estimates, we conducted a Monte Carlo sensitivity analysis[16, 18, 19]. The distributions of the simulated RR and the resulting PAF are shown in Figure S12. The sensitivity analysis indicated that parameter uncertainty affects the burden estimates, with the global age-standardized prevalence rate adjusting from the baseline model's 171.7 (95% UI: 157.3, 187.1) to 97.2 (95% UI: 26.6, 170.8) per 10,000,000 individuals, while the DALY rate changed from 0.5 (95% UI: 0.4, 0.6) to 0.3 (95% UI: 0.3–0.5) per 10,000,000 individuals (Table S10, Figure S13, Figure S14, and Figure S15).

However, it is noteworthy that in some SDI regions, the simulation results demonstrated good consistency with the baseline model (Table S10). For example, in Low-middle SDI regions, the age-standardized DALY rate remained stable at 0.3 per 10,000,000 individuals before and after the simulation; in High SDI regions, the age-standardized prevalence rate also remained largely stable (baseline: 80.8 vs. simulation: 75.0).The burden in Australasia (age-standardized prevalence rate 490.1) and Western Europe (432.2) is much higher than that in East Asia (36.1) and Southeast Asia (22.7). This significant regional difference remained consistent in both the initial estimation and the Monte Carlo simulation, thereby confirming the reliability of these core trends. Concurrently, we also observed that in regions with a higher baseline burden, such as High-income North America, the estimates were more sensitive to parameter fluctuations, with its prevalence rate decreasing from 508.2 to 65.2 per 10,000,000 individuals.

Overall, although parameter uncertainty may cause fluctuations in the absolute values of the attributed burden in some regions, the disease burden trends, inter-regional differences, and the identification of high-risk populations reported in this study are robust. Therefore, our baseline estimates provide a reliable and valuable frame of reference for understanding the global health impact of MASLD-IBD comorbidity and for guiding public health strategies.

## BAPC model

### Model Establishment and Assumptions

We employed the Bayesian Age-Period-Cohort (BAPC) model[20, 21] to forecast age-standardized prevalence and DALYs rates of MASLD-IBD comorbidity from 2021 to 2050. This model captures the intrinsic relationships between disease burden and three key demographic dimensions: age, period, and birth cohort. Its core formulation is expressed as:

$$Y_{ap} Poisson(E_{ap}\cdot e^{\mu+\alpha_{a}+\beta_{p}+\gamma_{c}})$$

$$log{(R}_{ap})=\mu+\alpha_{a}+\beta_{p}+\gamma_{c}$$

Where *Y_ap_* denotes observed counts for age group α and period *p*, *E_ap_* is the person-years at risk offset, and *R_ap_* represents the disease rate. The model assumes independent and additive effects of age (*α_a_*), period (*β_p_*), and cohort (*γ_c_*) on observed disease patterns. To ensure methodological robustness, we further hypothesized that: (i) the RR and prevalence (*p*) of MASLD among IBD patients remain constant across years, age groups, and countries; (ii) trends are smoothed via random walk priors (*α_a_∼RW1(σ^2^α)*, etc.); and (iii) sum-to-zero constraints (*∑α_a_*=0) resolve identifiability issues.

### Training and Fitting Process

Using age-stratified MASLD-IBD comorbidity data from the GBD 2021 database (1990–2021), we structured the input as a three-dimensional array (age group × period × sex). Bayesian inference was implemented via the INLA package in R, which employs integrated nested Laplace approximation to estimate posterior marginal distributions of parameters. The computational framework specifies:

$$\tau_{\alpha} \Gamma(0.001,0.001)$$

$$\tau_{\beta} \Gamma(0.001,0.001)$$

$$\tau_{\gamma} \Gamma(0.001,0.001)$$

Where *τ=1/σ^2^* denotes precision parameters, with gamma hyperpriors (*Γ*(0.001,0.001)) controlling the smoothness of random walks. Compared to conventional MCMC simulation, this approach offers superior computational efficiency, avoids convergence diagnostics, and quantifies uncertainty for all parameters through posterior distributions (e.g., 95% credible intervals for age effects *α_a_*).

### Goodness of Fit and Cross-Validation

We evaluated the goodness of fit of the BAPC model by comparing the predicted prevalence and DALYs rates with the actual observed values from the GBD 2021 database during the period 1990 to 2021. The evaluation metrics included the Root Mean Square Error (RMSE, defined as $RMSE=\sqrt{\frac{1}{n}\sum_{i=1}^{n} \left( \hat{y}_{i}-y_{i} \right)^{2}}$) and the coefficient of determination (*R*², defined as $R^{2}=1-\frac{\sum_{i=1}^{n} {(y_{i}-\hat{y}_{i})}^{2}}{\sum_{i=1}^{n} \left( y_{i}-\overline{y}_{i} \right)^{2}}$) to quantify the deviation between predicted and actual values and the model's explanatory power for the observed data variation, respectively[22]. The results (Table S9) showed that the fit for all age groups was highly statistically significant (all *p*-values < 1e⁻¹⁵). The coefficients of determination (*R*²) were consistently very high, ranging from 0.896 to 0.996, indicating that the model explained the vast majority (89.6% to 99.6%) of the variation in the observed data. Specifically, groups at both ends of the age distribution (15-19 years and ≥85 years) demonstrated the best fit, with *R*² values all exceeding 0.987 (reaching up to 0.996) and relatively small RMSE values (15-19 years: 4.01; ≥95 years: 16.4). Middle-aged groups (e.g., 35-64 years) exhibited relatively higher RMSE values (approximately 84.4 to 130), but their *R*² values remained excellent (0.896 to 0.915), indicating that although the absolute deviation between predicted and actual values was somewhat larger, the model's ability to explain data variation in these age groups was still very strong. Overall, the extremely low *p*-values, *R*² values close to 1, and acceptable RMSE levels collectively confirm that the BAPC model exhibits excellent goodness of fit across the entire age range and observation period.

To validate the model, we employed the Leave-One-Out Cross-Validation approach, a rigorous cross-validation strategy. The core principle of this method is: within a dataset containing multiple time points, each individual time point (year) is sequentially designated as an independent test set, while the model is trained using data from all remaining time points (years)[23, 24]. Specifically for this study, the BAPC model was trained on data spanning from 1990 to 2020 (31 years), and its prediction for the year 2021 was generated as a single, independent test set output. Subsequently, the model's 2021 predictions were rigorously compared against the actual 2021 GBD data across all age groups (15-19 to 95+ years). The validation results demonstrated exceptional predictive accuracy, achieving a RMSE of 91.993 and a Coefficient of Determination (*R*²) of 0.997 (Figure S16). This robust validation process thereby confirms the model's reliability and strong predictive power for forecasting disease burden metrics.

### Parameter Selection

The selection of model parameters was guided by the principle of parsimony while ensuring the model adequately captured the complex temporal and demographic trends. The model includes random effects for age, period, and cohort to account for non-linear trends. The choice of the BAPC model over other alternatives like Generalized Additive Models[25] or Smoothed Spline Models[26] was based on its superior performance in predicting short-term and medium-term disease burdens. This model's ability to simultaneously analyze age-related changes, temporal trends (period effects), and generational differences (cohort effects) makes it particularly well-suited for projecting long-term disease trajectories. The use of the Bayesian framework also allowed for the incorporation of prior knowledge and provided credible intervals for all projections, which are essential for robust forecasting.

## GATHER Checklist

**Table S2.** Guidelines for Accurate and Transparent Health Estimates Reporting (GATHER) checklist

| Item# | Checklist item | Reporting location |
| --- | --- | --- |
| Objectives and funding | |  |
| 1 | Define the indicator(s), populations (including age, sex, and geographic entities), and time period(s) for which estimates were made. | Appendix section “Supplementary methods” |
| 2 | List the funding sources for the work. | Main text Funding section |
| Data Inputs | |  |
| For all data inputs from multiple sources that are synthesized as part of the study: | |  |
| 3 | Describe how the data were identified and how the data were accessed. | Main text methods section: “Data Source” |
| 4 | Specify the inclusion and exclusion criteria. Identify all ad‐hoc exclusions. | Supplementary methods “The search strategy to estimate the RR in Equation 1” |
| 5 | Provide information on all included data sources and their main characteristics. For each data source used, report reference information or contact name/institution, population represented, data collection method, year(s) of data collection, sex and age range, diagnostic criteria or measurement method, and sample size, as relevant. | As detailed in the “Data Source” the complete list of sources is available via the Global Health Exchange Sources tool: https://ghdx.healthdata.org/gbd-2021/sources |
| 6 | Identify and describe any categories of input data that have potentially important biases (e.g., based on characteristics listed in item 5). | Data inputs in excel format available on the  GHDx (https://ghdx.healthdata.org/) |
| For data inputs that contribute to the analysis but were not synthesized as part of the study: | |  |
| 7 | Describe and give sources for any other data inputs. | N/A |
| For all data inputs: | |  |
| 8 | Provide all data inputs in a file format from which data can be efficiently extracted (e.g., a spreadsheet rather than a PDF), including all relevant meta‐data listed in item 5. For any data inputs that cannot be shared because of ethical or legal reasons, such as third‐party ownership, provide a contact name or the name of the institution that retains the right to the data. | Data inputs in excel format available on the  GHDx (https://ghdx.healthdata.org/) |
| Data analysis | |  |
| 9 | Provide a conceptual overview of the data analysis method. A diagram may be helpful. | Main text Methods |
| 10 | Provide a detailed description of all steps of the analysis, including mathematical formulae. This description should cover, as relevant, data cleaning, data pre‐processing, data adjustments and weighting of data sources, and mathematical or statistical model(s). | Main text Methods |
| 11 | Describe how candidate models were evaluated and how the final model(s) were selected. | Main text Methods; Supplementary methods: “The search strategy to estimate the RR in Equation 1” |
| 12 | Provide the results of an evaluation of model performance, if done, as well as the results of any relevant sensitivity analysis. | Main text Methods section “Prediction Analysis” |
| 13 | Describe methods for calculating uncertainty of the estimates. State which sources of uncertainty were, and were not, accounted for in the uncertainty analysis. | Main text Methods section “Population Attributable Fraction” and main text discussion section paragraph 7 |
| 14 | State how analytic or statistical source code used to generate estimates can be accessed. | Main text Methods |
| Results and Discussion | |  |
| 15 | Provide published estimates in a file format from which data can be efficiently extracted. | The results can be efficiently extracted at  https://vizhub.healthdata.org/gbd-results/ |
| 16 | Report a quantitative measure of the uncertainty of the estimates (e.g. uncertainty intervals). | UIs and CIs given for all findings, including in the text, figures, and tables in the main text and SM;  online viz tools (see information above) |
| 17 | Interpret results in light of existing evidence. If updating a previous set of estimates, describe the reasons for changes in estimates. | Main text Discussion |
| 18 | Discuss limitations of the estimates. Include a discussion of any modelling assumptions or data limitations that affect interpretation of the estimates. | Main text Discussion |

## TITAN Guideline Checklist

| **TITAN Guideline Checklist 2025** | | | |
| --- | --- | --- | --- |
| **Topic** | **Item** | **Description** | **Page number** |
| Artificial Intelligence (AI) (some journals may prefer this in the methods and/or acknowledgments section and it should also be declared in the cover letter) | 1 | Declaration of whether any AI was used in the research and manuscript development  State no, if that’s the case.  If yes, proceed to item 5a. | P3 |
|  | 1a | Purpose and Scope of AI Use  - Precisely state why AI was employed (e.g. development of research questions, language drafting, statistical analysis/summarisation, image annotation, etc).  - Was generative AI utilised and if so, how?  - Clarify the stage(s) of the reporting workflow affected (planning, writing, revisions, figure creation). - Confirmation that the author(s) take responsibility for the integrity of the content affected/generated | NA |
|  | 1b | AI Tool(s) and Configuration  - Name each system (vendor, model, major version/date).  - State the date it was used  - Specify relevant parameters (e.g. prompt length, plug-ins, fine-tuning, temperature).  - Declare whether the tool operated locally on-premises, or via a cloud API and any integrations with other systems. | NA |
|  | 1c | Data Inputs and Safeguards  - Describe categories of data provided to the AI (patient text, de-identified images, literature abstracts).  - Confirm that all inputs were de-identified and compliant with GDPR/HIPAA.  - Note any institutional approvals or data-sharing agreements obtained. | NA |
|  | 1d | Human Oversight and Verification  - Identify the supervising author(s) who reviewed every AI output.  - Detail the process for fact-checking, clinical accuracy checks  - State whether any AI-generated text/figures were edited or discarded. - Acknowledge the limitations of AI and its use | NA |
|  | 1e | Bias, Ethics and Regulatory Compliance  - Outline steps taken to detect and mitigate algorithmic bias (e.g. cross-checking against under-represented populations).  - Affirm adherence to relevant ethical frameworks.  - Disclose any conflicts of interest or financial ties to AI vendors. | NA |
|  | 1f | Reproducibility and Transparency  - Provide the exact prompts or code snippets (as supplementary material if lengthy).  - Supply version-controlled logs or model cards where possible.  - if applicable, state repository, hyperlink or digital object identifier (DOI) where AI-generated artefacts can be accessed, enabling attempts at independent replication of the query/input. | NA |

# Supplementary Results in Appendix Figures


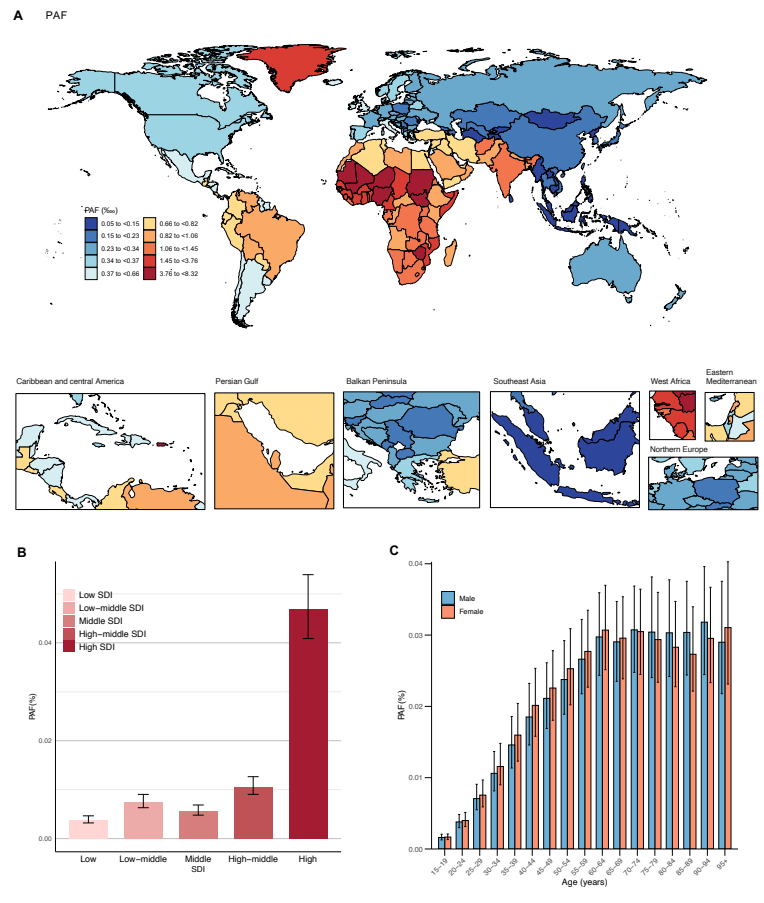


**Figure S2.** Age-standardized PAF of MASLD–IBD comorbidity in 2021 stratified by country and territory (A), SDI (B), and age and sex (C).

Abbreviations: PAF=population attributable fraction. MASLD=metabolic dysfunction-associated steatotic liver disease. IBD=inflammatory bowel disease. SDI=socio-demographic index.


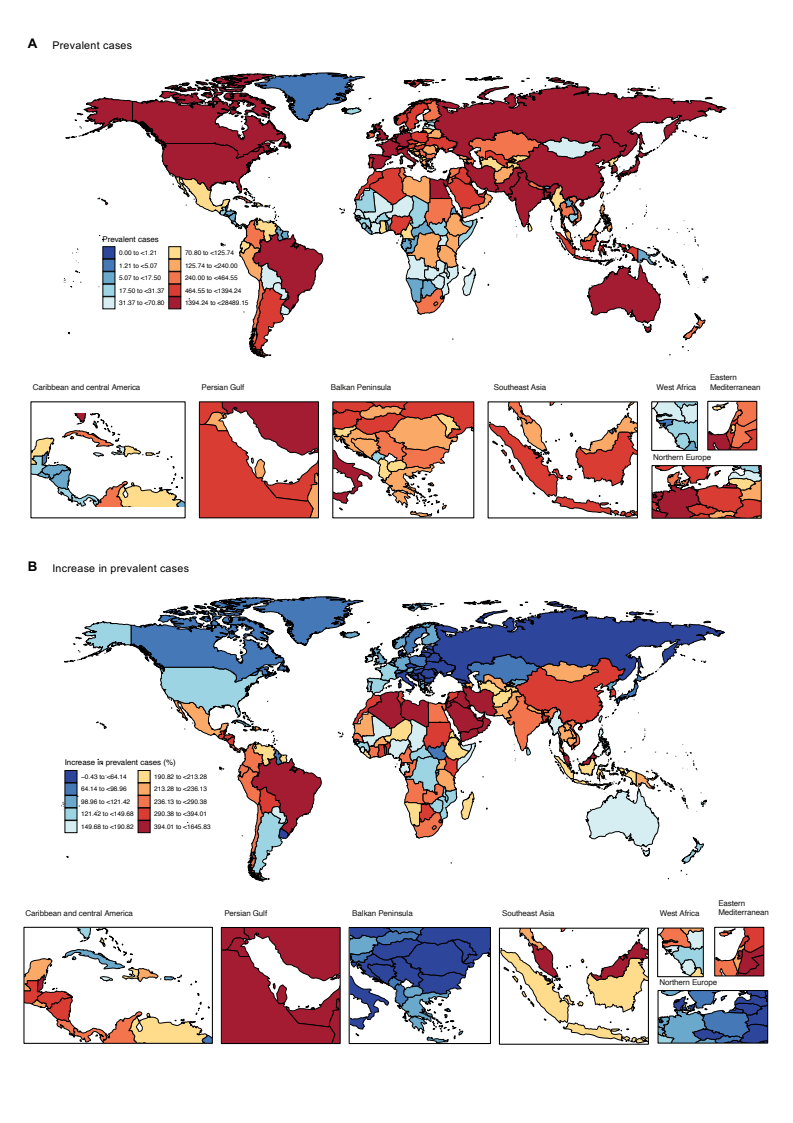
**Figure S3**. Prevalent cases (A) of MASLD–IBD comorbidity in the 204 countries and territories in 2021. Relative changes in the number of prevalent cases (B) of MASLD–IBD comorbidity in the 204 countries and territories between 1990 and 2021.

Abbreviations: MASLD=metabolic dysfunction-associated steatotic liver disease. IBD=inflammatory bowel disease.


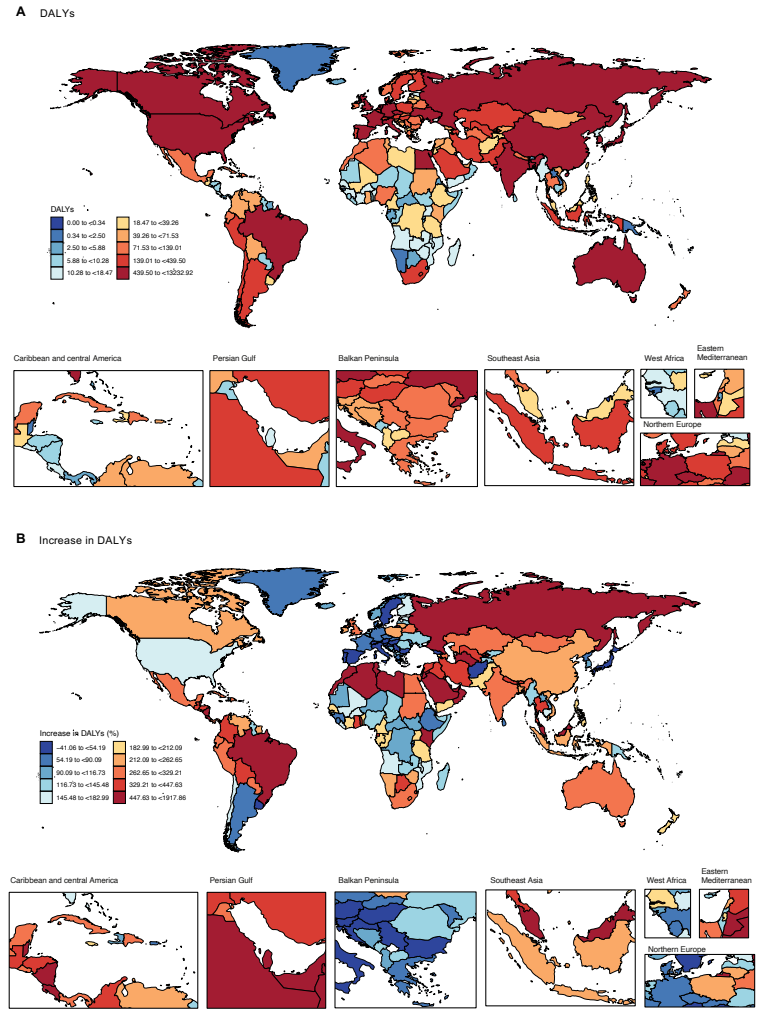
**Figure S4.** DALYs (A) of MASLD–IBD comorbidity in the 204 countries and territories in 2021. Relative changes in DALYs (B) of MASLD–IBD comorbidity in the 204 countries and territories between 1990 and 2021.

Abbreviations: DALYs=disability adjusted life years. MASLD=metabolic dysfunction-associated steatotic liver disease. IBD=inflammatory bowel disease.


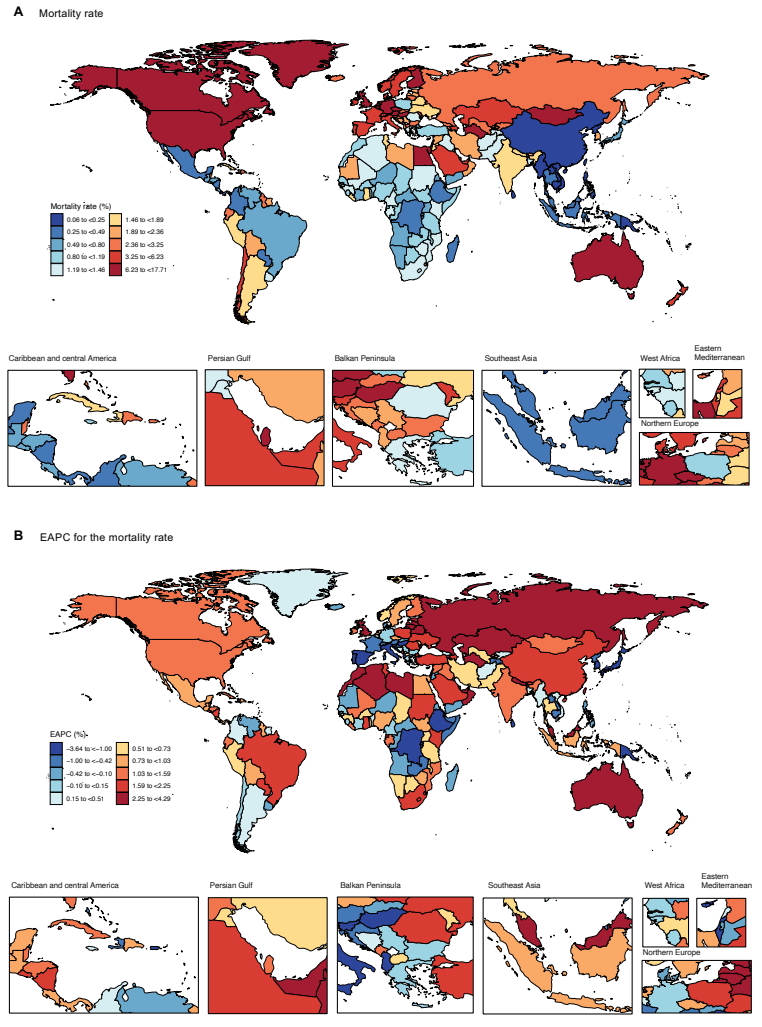


**Figure S5.** The age-standardized mortality (A) rate (per 10,000,000 individuals) due to MASLD–IBD comorbidity in 204 countries and territories in 2021; EAPC in the age-standardized mortality (B) rate of MASLD–IBD comorbidity in 204 countries and territories from 1990 to 2021.

Abbreviations: MASLD=metabolic dysfunction-associated steatotic liver disease. IBD=inflammatory bowel disease. EAPC=estimated annual percentage change.


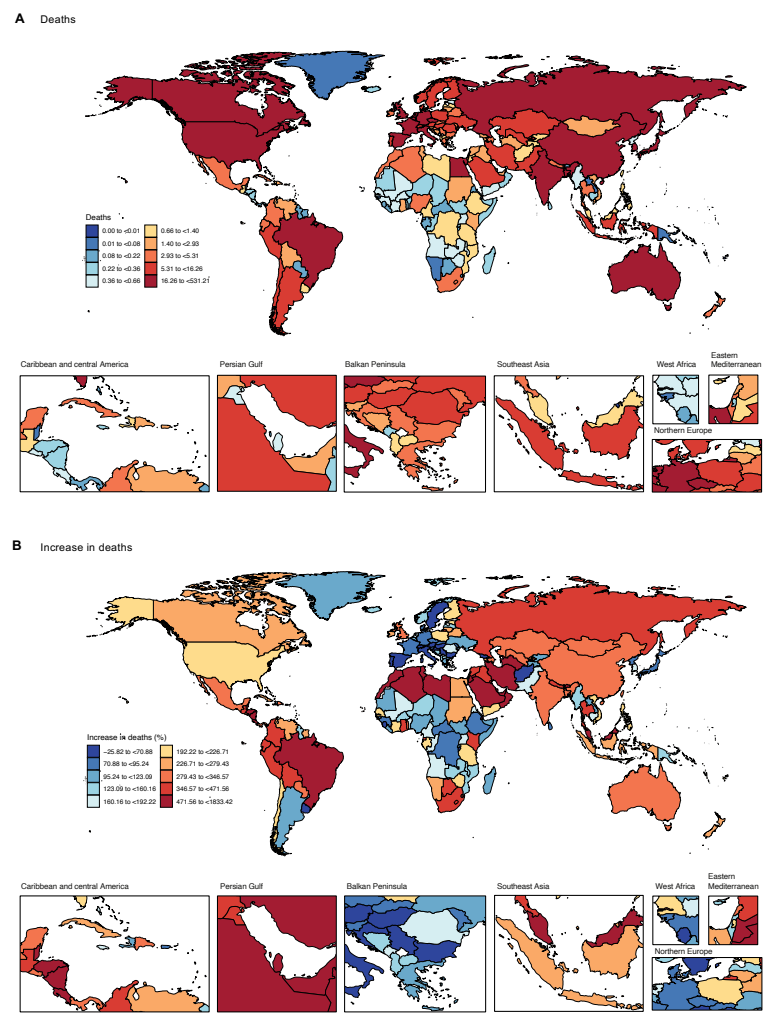


**Figure S6.** Deaths (A) of MASLD–IBD comorbidity in the 204 countries and territories in 2021. Relative changes in deaths (B) of MASLD-IBD comorbidity in the 204 countries and territories between 1990 and 2021.

Abbreviations: MASLD=metabolic dysfunction-associated steatotic liver disease. IBD=inflammatory bowel disease.


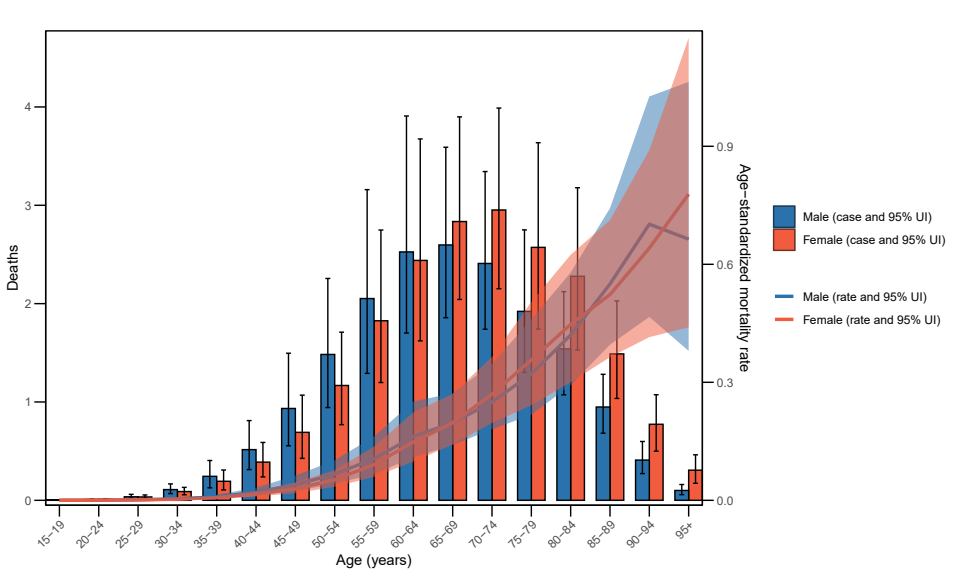
**Figure S7.** The global numbers and age-standardized rate (per 10,000,000 individuals) for the deaths of MASLD–IBD comorbidity in 2021, stratified by age and sex.

Abbreviations: MASLD=metabolic dysfunction-associated steatotic liver disease. IBD=inflammatory bowel disease.


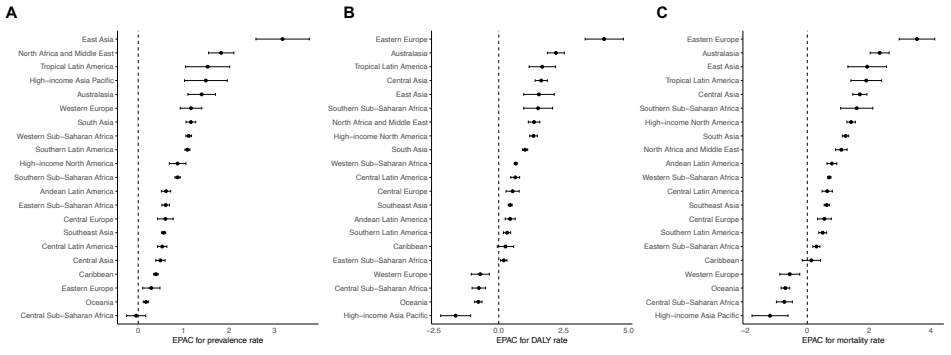


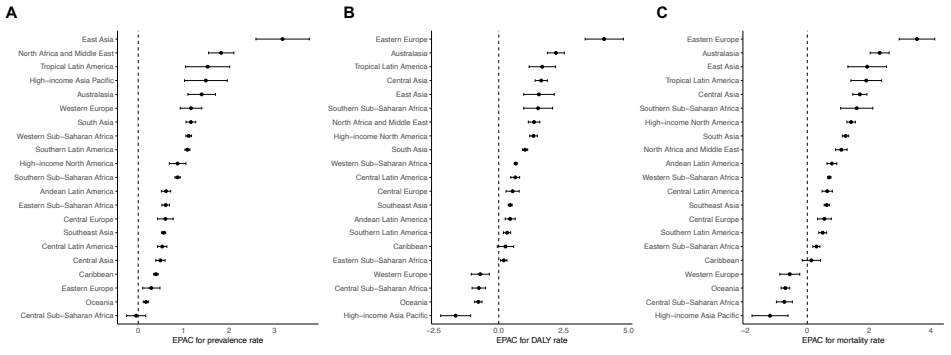
**Figure S8.** EAPC of the age-standardized prevalence (A), DALY (B), and mortality (C) rate (per 10,000,000 individuals) of MASLD–IBD comorbidity from 1990 to 2021 for the 21 GBD regions.

Abbreviations: EAPC=estimated annual percentage change. DALY=disability adjusted life year. MASLD=metabolic dysfunction-associated steatotic liver disease. IBD=inflammatory bowel disease. GBD=global disease of burden.

**
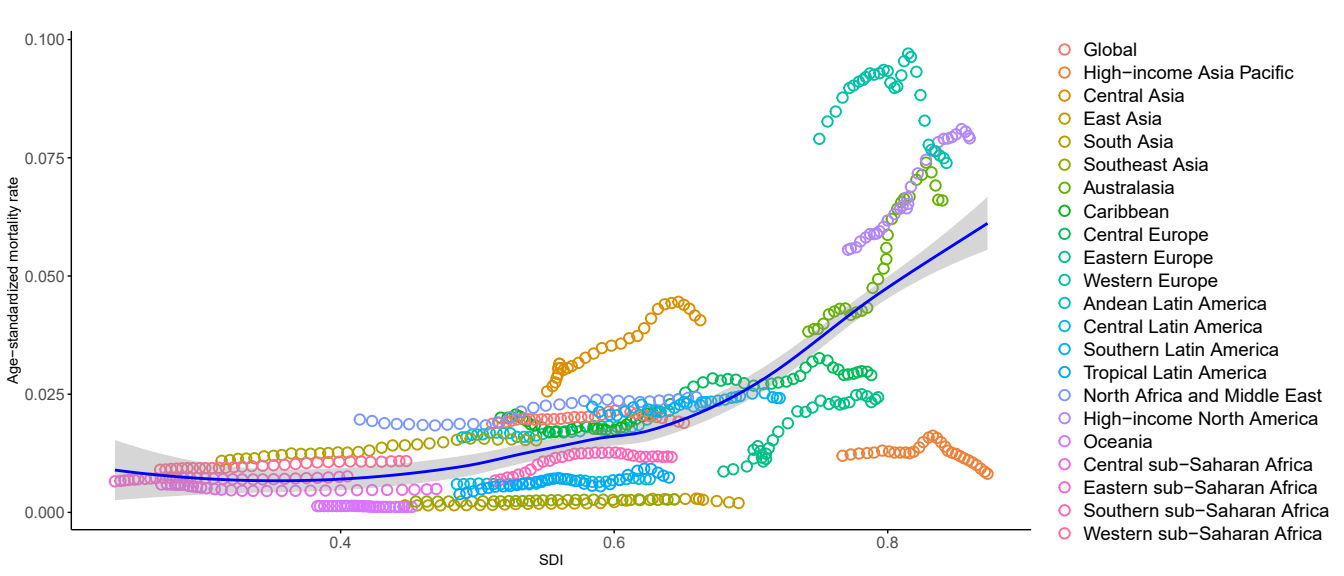
**

**Figure. S9.** The age-standardized mortality rate (per 10,000,000 individuals) of MASLD–IBD comorbidity in the 21 GBD regions by SDI during 1990–2021. Each dot represents the disease burden for a year in that region. The blue line, a locally weighted scatterplot smoothing smoother, presents the expected global values based on the SDI values.

Abbreviations: GBD=global disease of burden. MASLD=metabolic dysfunction-associated steatotic liver disease. IBD=inflammatory bowel disease. SDI=socio-demographic index.


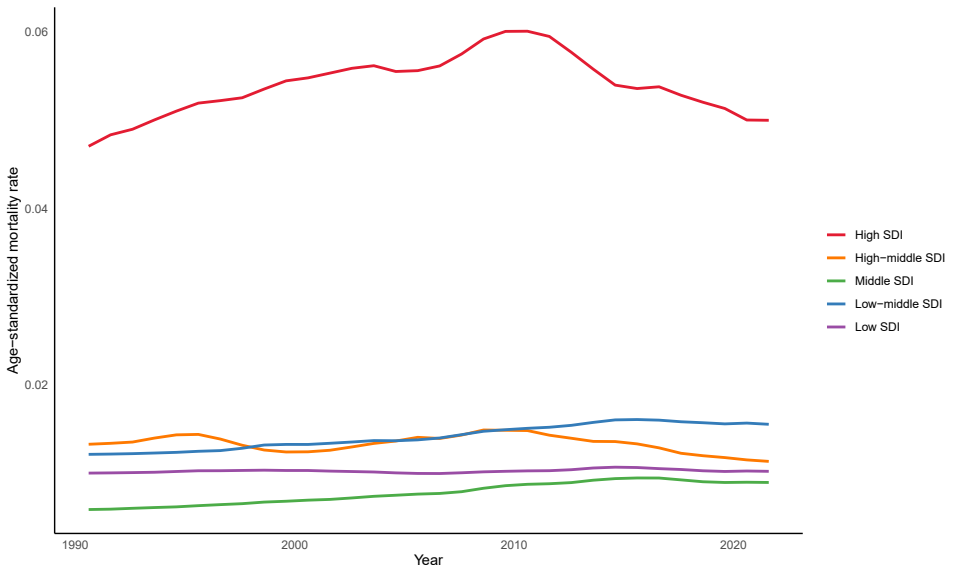
**Figure S10.** The trends in the age-standardized mortality rate (per 10,000,000 individuals) of MASLD-IBD comorbidity from 1990 to 2021, stratified by the SDI level.

Abbreviations: MASLD=metabolic dysfunction-associated steatotic liver disease. IBD=inflammatory bowel disease. SDI=socio-demographic index.


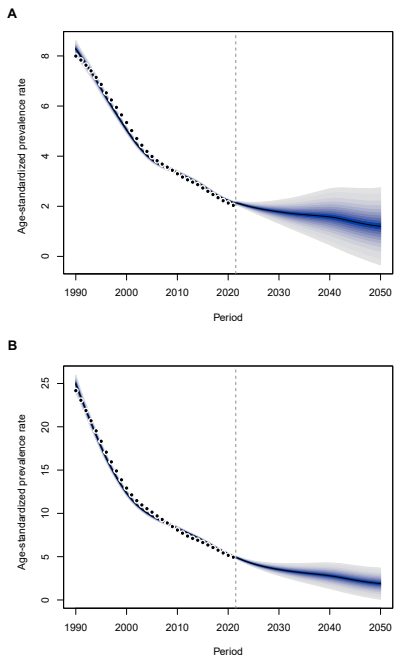


**Figure S11.** Global predictions of MASLD-IBD comorbidity related age-standardized prevalence, DALY and mortality rate (per 10,000,000 individuals) from 2021 to 2050. (A) The age-standardized prevalence rate among the female population. (B) The age-standardized DALY rate among the male population.

Abbreviations: MASLD=metabolic dysfunction-associated steatotic liver disease. IBD=inflammatory bowel disease. DALYs=disability adjusted life years.


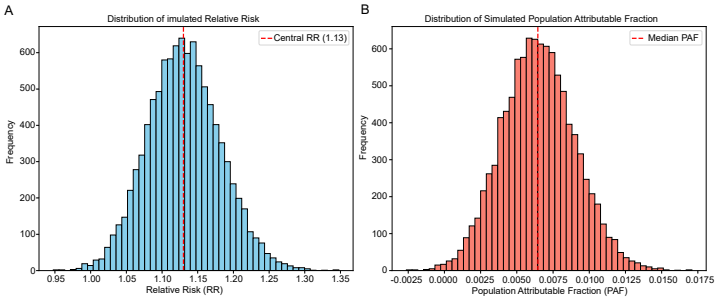


**Figure S12**. Sensitivity analysis of the impact of RR and prevalence (*p*) on the PAF. (A) The histogram displays the probability distribution of the simulated Relative Risk (RR) used in the sensitivity analysis. The distribution is based on the confidence intervals reported in the literature, with the vertical dashed line indicating the central estimate of RR (1.13) used in the primary analysis. (B) The histogram shows the resulting probability distribution of the PAF after 10,000 Monte Carlo simulations, where both RR and the prevalence of IBD in MASLD patients (*p*) were simultaneously varied according to their respective distributions. The vertical dashed line indicates the median PAF from these simulations, quantifying the uncertainty of the estimated disease burden.

Abbreviations: RR=relative risk. PAF=population attributable fraction.

**
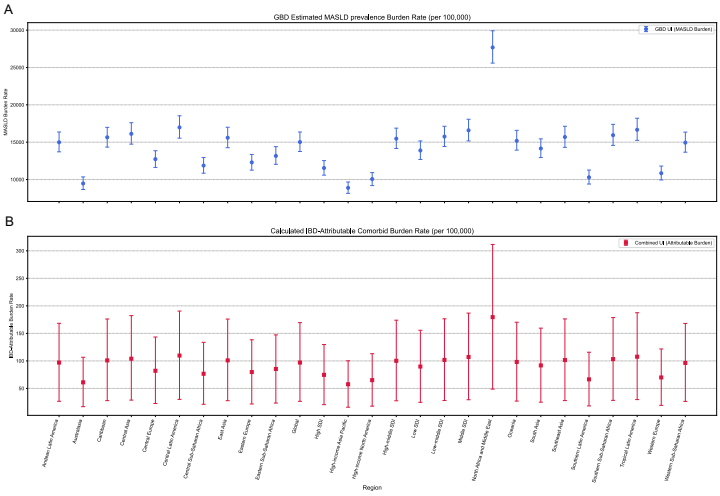
**

**Figure S13.** Regional comparison of MASLD burden and IBD-attributable comorbid burden of prevalence with uncertainty intervals (2021). (A) GBD-estimated MASLD burden rate (per 100,000 population) with 95% UI. (B) Calculated IBD-attributable comorbid MASLD burden rate (per 100,000 population) after propagating uncertainties from both MASLD prevalence and PAF. Blue circles (Panel A): Point estimates of MASLD prevalence with error bars representing GBD-reported 95% UI. Red squares (Panel B): Median estimates of IBD-attributable burden with error bars showing combined 95% UI after Monte Carlo simulation (10,000 iterations)

Abbreviations: IBD=inflammatory bowel disease. MASLD=metabolic dysfunction-associated steatotic liver disease. PAF=population attributable fraction; UI, uncertainty interval.

**
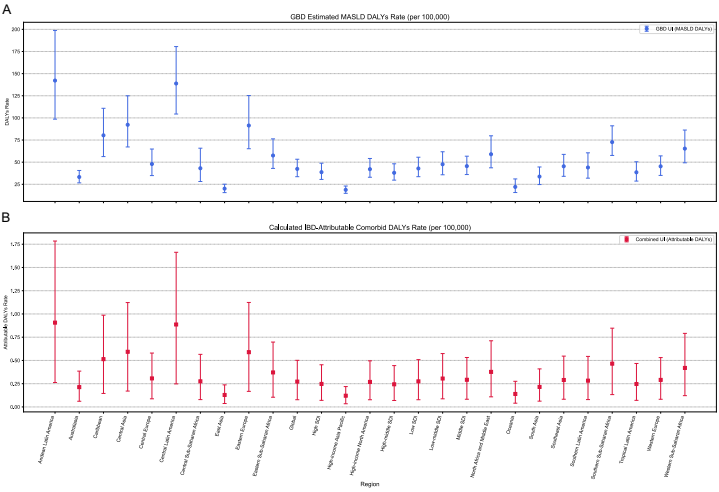
**

**Figure S14.** Regional comparison of MASLD burden and IBD-attributable comorbid burden of DALYs with uncertainty intervals (2021). (A) GBD-estimated MASLD burden rate (per 100,000 population) with 95% UI. (B) Calculated IBD-attributable comorbid MASLD burden rate (per 100,000 population) after propagating uncertainties from both MASLD DALYs and PAF. Blue circles (Panel A): Point estimates of MASLD DALYs with error bars representing GBD-reported 95% UI. Red squares (Panel B): Median estimates of IBD-attributable burden with error bars showing combined 95% UI after Monte Carlo simulation (10,000 iterations)

Abbreviations: IBD=inflammatory bowel disease. MASLD=metabolic dysfunction-associated steatotic liver disease. PAF=population attributable fraction; UI, uncertainty interval.


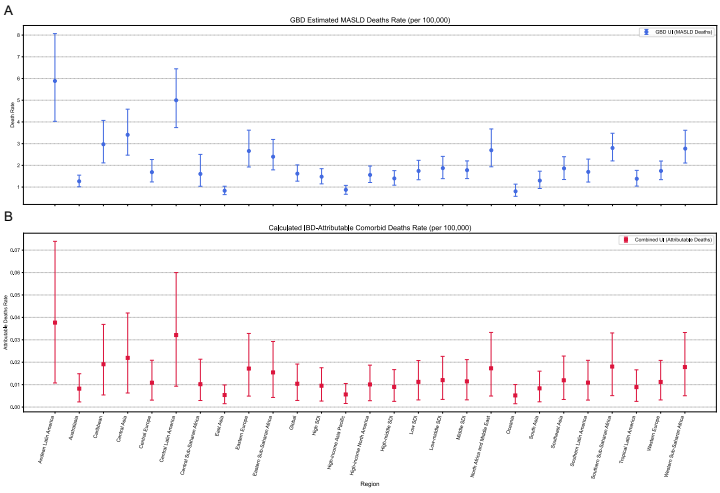


**Figure S15.** Regional comparison of MASLD burden and IBD-attributable comorbid burden of deaths with uncertainty intervals (2021). (A) GBD-estimated MASLD burden rate (per 100,000 population) with 95% UI. (B) Calculated IBD-attributable comorbid MASLD burden rate (per 100,000 population) after propagating uncertainties from both MASLD deaths and PAF. Blue circles (Panel A): Point estimates of MASLD deaths with error bars representing GBD-reported 95% UI. Red squares (Panel B): Median estimates of IBD-attributable burden with error bars showing combined 95% UI after Monte Carlo simulation (10,000 iterations)

Abbreviations: IBD=inflammatory bowel disease. MASLD=metabolic dysfunction-associated steatotic liver disease. PAF=population attributable fraction; UI, uncertainty interval.

**
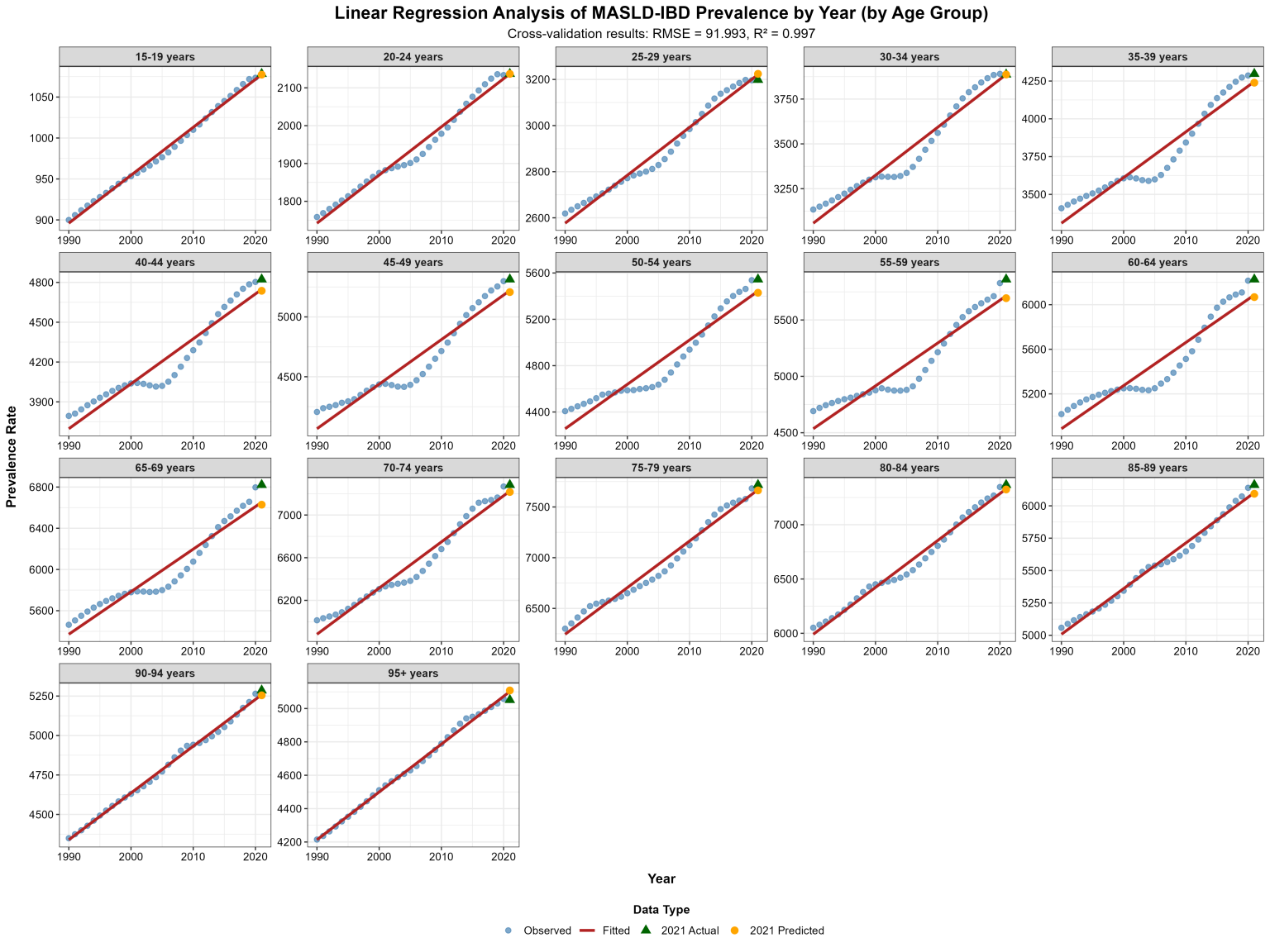
**

**Figure S16.** Goodness-of-fit and cross-validation of the linear regression model for MASLD-IBD prevalence by age group. Blue circles represent the observed annual prevalence rates. The red line indicates the fitted values from the linear regression model, demonstrating the model's fit to the historical data. The orange circle shows the predicted prevalence for 2021. The green triangle shows the actual observed prevalence for 2021.

# Supplementary Results in Appendix Tables

**Table S3.** PAF of MASLD–IBD comorbidity in 1990 and 2021

| **Location** | **PAF 1990 ‱ (95% UI)** | **PAF 2021 ‱ (95% UI)** |
| --- | --- | --- |
| **Global** | 1.04 (0.90, 1.20) | 1.24 (1.07, 1.46) |
| **SDI** |  |  |
| High SDI | 3.78 (3.35, 4.31) | 4.68 (4.09, 5.39) |
| High-middle SDI | 0.90 (0.78, 1.06) | 1.06 (0.90, 1.26) |
| Middle SDI | 0.29 (0.24, 0.35) | 0.57 (0.48, 0.69) |
| Low-middle SDI | 0.50 (0.42, 0.60) | 0.74 (0.63, 0.90) |
| Low SDI | 0.33 (0.27, 0.39) | 0.38 (0.32, 0.47) |
| **Region** |  |  |
| High-income Asia Pacific | 0.88 (0.75, 1.05) | 1.42 (1.22, 1.71) |
| Central Asia | 0.93 (0.79, 1.12) | 1.18 (0.99, 1.43) |
| East Asia | 0.14 (0.11, 0.16) | 0.30 (0.25, 0.36) |
| South Asia | 0.70 (0.59, 0.85) | 1.11 (0.93, 1.35) |
| Southeast Asia | 0.11 (0.10, 0.14) | 0.16 (0.13, 0.19) |
| Australasia | 5.38 (4.61, 6.47) | 6.64 (5.74, 7.81) |
| Caribbean | 0.61 (0.51, 0.72) | 0.76 (0.64, 0.91) |
| Central Europe | 1.87 (1.61, 2.19) | 2.26 (1.95, 2.66) |
| Eastern Europe | 1.10 (0.93, 1.33) | 1.25 (1.06, 1.50) |
| Western Europe | 4.32 (3.79, 4.88) | 5.41 (4.69, 6.30) |
| Andean Latin America | 0.24 (0.21, 0.29) | 0.34 (0.29, 0.41) |
| Central Latin America | 0.10 (0.09, 0.13) | 0.15 (0.12, 0.18) |
| Southern Latin America | 1.19 (1.01, 1.44) | 1.54 (1.31, 1.89) |
| Tropical Latin America | 0.26 (0.22, 0.32) | 0.59 (0.50, 0.71) |
| North Africa and Middle East | 0.53 (0.44, 0.63) | 0.88 (0.73, 1.06) |
| High-income North America | 5.87 (5.20, 6.72) | 6.86 (6.01, 7.89) |
| Oceania | 0.11 (0.09, 0.13) | 0.12 (0.10, 0.14) |
| Central Sub-Saharan Africa | 0.20 (0.17, 0.25) | 0.21 (0.18, 0.26) |
| Eastern Sub-Saharan Africa | 0.17 (0.14, 0.20) | 0.21 (0.18, 0.25) |
| Southern Sub-Saharan Africa | 0.25 (0.21, 0.30) | 0.39 (0.33, 0.47) |
| Western Sub-Saharan Africa | 0.20 (0.17, 0.24) | 0.24 (0.21, 0.30) |
| **Country** |  |  |
| Afghanistan | 0.36 (0.30, 0.43) | 0.37 (0.31, 0.45) |
| Albania | 1.42 (1.19, 1.73) | 2.01 (1.68, 2.46) |
| Algeria | 0.52 (0.43, 0.63) | 0.95 (0.79, 1.18) |
| American Samoa | 0.14 (0.12, 0.17) | 0.20 (0.16, 0.24) |
| Andorra | 4.47 (3.78, 5.37) | 5.90 (5.00, 7.10) |
| Angola | 0.18 (0.15, 0.23) | 0.21 (0.18, 0.26) |
| Antigua and Barbuda | 0.72 (0.61, 0.86) | 1.01 (0.84, 1.23) |
| Argentina | 1.18 (0.99, 1.43) | 1.46 (1.23, 1.78) |
| Armenia | 1.13 (0.94, 1.36) | 1.63 (1.37, 2.00) |
| Australia | 5.36 (4.55, 6.48) | 6.72 (5.78, 7.93) |
| Austria | 3.69 (3.13, 4.37) | 4.75 (4.09, 5.74) |
| Azerbaijan | 0.97 (0.81, 1.16) | 1.49 (1.23, 1.83) |
| Bahamas | 0.70 (0.59, 0.84) | 0.88 (0.74, 1.07) |
| Bahrain | 0.65 (0.54, 0.78) | 0.97 (0.81, 1.18) |
| Bangladesh | 0.67 (0.56, 0.81) | 1.23 (1.04, 1.51) |
| Barbados | 0.88 (0.77, 1.03) | 1.18 (0.99, 1.44) |
| Belarus | 1.27 (1.07, 1.53) | 1.52 (1.27, 1.83) |
| Belgium | 2.84 (2.53, 3.24) | 3.87 (3.32, 4.61) |
| Belize | 0.41 (0.35, 0.49) | 0.61 (0.51, 0.74) |
| Benin | 0.16 (0.14, 0.20) | 0.22 (0.18, 0.27) |
| Bermuda | 1.00 (0.83, 1.22) | 1.27 (1.08, 1.54) |
| Bhutan | 0.62 (0.51, 0.75) | 1.14 (0.96, 1.38) |
| Bolivia (Plurinational State of) | 0.20 (0.17, 0.24) | 0.29 (0.24, 0.36) |
| Bosnia and Herzegovina | 1.40 (1.16, 1.71) | 1.89 (1.59, 2.31) |
| Botswana | 0.20 (0.17, 0.24) | 0.35 (0.29, 0.42) |
| Brazil | 0.26 (0.22, 0.32) | 0.60 (0.51, 0.72) |
| Brunei Darussalam | 0.22 (0.18, 0.27) | 0.32 (0.26, 0.39) |
| Bulgaria | 2.02 (1.69, 2.46) | 2.18 (1.84, 2.59) |
| Burkina Faso | 0.16 (0.13, 0.19) | 0.20 (0.17, 0.25) |
| Burundi | 0.18 (0.15, 0.22) | 0.19 (0.16, 0.23) |
| Cabo Verde | 0.24 (0.20, 0.28) | 0.45 (0.38, 0.54) |
| Cambodia | 0.08 (0.07, 0.10) | 0.11 (0.09, 0.14) |
| Cameroon | 0.20 (0.16, 0.24) | 0.23 (0.19, 0.28) |
| Canada | 10.86 (10.17, 11.53) | 11.12 (9.56, 13.10) |
| Central African Republic | 0.17 (0.14, 0.21) | 0.19 (0.15, 0.23) |
| Chad | 0.16 (0.14, 0.20) | 0.17 (0.14, 0.21) |
| Chile | 1.15 (0.96, 1.39) | 1.75 (1.47, 2.13) |
| China | 0.13 (0.11, 0.16) | 0.30 (0.25, 0.36) |
| Colombia | 0.24 (0.19, 0.29) | 0.37 (0.30, 0.44) |
| Comoros | 0.20 (0.17, 0.25) | 0.31 (0.26, 0.38) |
| Congo | 0.23 (0.20, 0.28) | 0.32 (0.27, 0.38) |
| Cook Islands | 0.16 (0.14, 0.20) | 0.23 (0.19, 0.28) |
| Costa Rica | 0.13 (0.11, 0.16) | 0.20 (0.16, 0.24) |
| Côte d'Ivoire | 0.18 (0.15, 0.22) | 0.24 (0.20, 0.29) |
| Croatia | 2.24 (1.87, 2.72) | 2.69 (2.29, 3.25) |
| Cuba | 0.78 (0.65, 0.94) | 1.03 (0.87, 1.23) |
| Cyprus | 2.51 (2.12, 3.11) | 3.76 (3.20, 4.55) |
| Czechia | 5.92 (5.06, 7.09) | 6.89 (5.96, 7.96) |
| Democratic People's Republic of Korea | 0.17 (0.14, 0.20) | 0.19 (0.16, 0.23) |
| Democratic Republic of the Congo | 0.21 (0.18, 0.25) | 0.20 (0.17, 0.25) |
| Denmark | 5.66 (4.90, 6.40) | 6.04 (5.23, 6.96) |
| Djibouti | 0.21 (0.17, 0.26) | 0.31 (0.26, 0.38) |
| Dominica | 0.58 (0.48, 0.70) | 0.85 (0.72, 1.03) |
| Dominican Republic | 0.48 (0.39, 0.58) | 0.67 (0.56, 0.81) |
| Ecuador | 0.24 (0.20, 0.29) | 0.35 (0.29, 0.42) |
| Egypt | 0.66 (0.56, 0.78) | 0.90 (0.75, 1.09) |
| El Salvador | 0.09 (0.07, 0.11) | 0.15 (0.12, 0.18) |
| Equatorial Guinea | 0.18 (0.16, 0.22) | 0.27 (0.22, 0.32) |
| Eritrea | 0.19 (0.15, 0.23) | 0.24 (0.20, 0.30) |
| Estonia | 1.27 (1.07, 1.52) | 1.47 (1.25, 1.76) |
| Eswatini | 0.17 (0.15, 0.21) | 0.28 (0.23, 0.34) |
| Ethiopia | 0.15 (0.13, 0.18) | 0.19 (0.16, 0.23) |
| Fiji | 0.13 (0.11, 0.16) | 0.16 (0.13, 0.19) |
| Finland | 4.91 (4.43, 5.45) | 6.70 (5.66, 7.75) |
| France | 3.32 (3.05, 3.57) | 4.59 (3.94, 5.49) |
| Gabon | 0.26 (0.22, 0.31) | 0.32 (0.27, 0.38) |
| Gambia | 0.19 (0.16, 0.23) | 0.23 (0.19, 0.29) |
| Georgia | 1.43 (1.20, 1.73) | 1.71 (1.46, 2.09) |
| Germany | 6.19 (5.26, 7.32) | 8.44 (7.22, 10.03) |
| Ghana | 0.24 (0.20, 0.29) | 0.33 (0.28, 0.41) |
| Greece | 0.94 (0.83, 1.07) | 1.35 (1.15, 1.61) |
| Greenland | 5.45 (4.62, 6.54) | 6.35 (5.46, 7.44) |
| Grenada | 0.48 (0.40, 0.58) | 0.75 (0.63, 0.90) |
| Guam | 0.18 (0.14, 0.21) | 0.22 (0.19, 0.28) |
| Guatemala | 0.07 (0.06, 0.09) | 0.11 (0.09, 0.14) |
| Guinea | 0.19 (0.16, 0.23) | 0.22 (0.18, 0.26) |
| Guinea-Bissau | 0.17 (0.14, 0.20) | 0.21 (0.18, 0.26) |
| Guyana | 0.47 (0.39, 0.57) | 0.63 (0.52, 0.77) |
| Haiti | 0.38 (0.31, 0.45) | 0.47 (0.37, 0.57) |
| Honduras | 0.08 (0.06, 0.09) | 0.11 (0.09, 0.14) |
| Hungary | 4.86 (4.32, 5.45) | 5.53 (4.72, 6.49) |
| Iceland | 6.07 (5.42, 6.76) | 6.25 (5.36, 7.18) |
| India | 0.72 (0.60, 0.88) | 1.16 (0.98, 1.41) |
| Indonesia | 0.12 (0.10, 0.14) | 0.16 (0.13, 0.19) |
| Iran (Islamic Republic of) | 0.54 (0.45, 0.67) | 0.95 (0.79, 1.17) |
| Iraq | 0.33 (0.28, 0.40) | 0.61 (0.50, 0.75) |
| Ireland | 3.88 (3.27, 4.62) | 5.30 (4.43, 6.28) |
| Israel | 3.03 (2.56, 3.60) | 3.62 (3.05, 4.35) |
| Italy | 3.61 (3.14, 4.19) | 3.58 (3.06, 4.26) |
| Jamaica | 0.59 (0.49, 0.72) | 0.89 (0.75, 1.08) |
| Japan | 0.85 (0.71, 1.03) | 1.16 (0.98, 1.41) |
| Jordan | 0.61 (0.52, 0.72) | 1.02 (0.85, 1.24) |
| Kazakhstan | 1.11 (0.93, 1.36) | 1.32 (1.11, 1.60) |
| Kenya | 0.17 (0.15, 0.21) | 0.26 (0.22, 0.31) |
| Kiribati | 0.11 (0.09, 0.13) | 0.12 (0.10, 0.15) |
| Kuwait | 1.00 (0.87, 1.14) | 1.30 (1.06, 1.61) |
| Kyrgyzstan | 0.86 (0.72, 1.05) | 1.06 (0.89, 1.29) |
| Lao People's Democratic Republic | 0.08 (0.07, 0.10) | 0.12 (0.10, 0.15) |
| Latvia | 1.37 (1.16, 1.67) | 1.56 (1.31, 1.86) |
| Lebanon | 1.81 (1.51, 2.21) | 2.76 (2.31, 3.38) |
| Lesotho | 0.20 (0.17, 0.24) | 0.28 (0.23, 0.34) |
| Liberia | 0.18 (0.15, 0.22) | 0.24 (0.20, 0.29) |
| Libya | 0.30 (0.25, 0.37) | 0.80 (0.66, 0.99) |
| Lithuania | 1.54 (1.29, 1.87) | 1.93 (1.61, 2.32) |
| Luxembourg | 4.01 (3.42, 4.76) | 5.38 (4.60, 6.48) |
| Madagascar | 0.19 (0.16, 0.23) | 0.22 (0.18, 0.27) |
| Malawi | 0.17 (0.14, 0.20) | 0.20 (0.17, 0.25) |
| Malaysia | 0.14 (0.12, 0.17) | 0.22 (0.19, 0.27) |
| Maldives | 0.09 (0.07, 0.11) | 0.19 (0.16, 0.24) |
| Mali | 0.17 (0.14, 0.21) | 0.19 (0.15, 0.22) |
| Malta | 2.65 (2.20, 3.20) | 3.42 (2.89, 4.19) |
| Marshall Islands | 0.09 (0.07, 0.11) | 0.13 (0.11, 0.16) |
| Mauritania | 0.22 (0.18, 0.26) | 0.30 (0.25, 0.36) |
| Mauritius | 0.15 (0.12, 0.18) | 0.21 (0.18, 0.26) |
| Mexico | 0.04 (0.03, 0.05) | 0.05 (0.04, 0.06) |
| Micronesia (Federated States of) | 0.10 (0.08, 0.12) | 0.14 (0.11, 0.17) |
| Monaco | 5.24 (4.46, 6.25) | 5.89 (5.04, 7.03) |
| Mongolia | 0.65 (0.54, 0.78) | 0.97 (0.80, 1.18) |
| Montenegro | 1.84 (1.55, 2.21) | 2.36 (2.00, 2.83) |
| Morocco | 0.48 (0.40, 0.59) | 0.84 (0.71, 1.02) |
| Mozambique | 0.17 (0.14, 0.20) | 0.17 (0.14, 0.21) |
| Myanmar | 0.10 (0.08, 0.12) | 0.13 (0.11, 0.16) |
| Namibia | 0.24 (0.20, 0.29) | 0.34 (0.29, 0.41) |
| Nauru | 0.11 (0.09, 0.14) | 0.12 (0.10, 0.15) |
| Nepal | 0.69 (0.57, 0.83) | 1.02 (0.83, 1.25) |
| Netherlands | 7.43 (6.72, 8.13) | 9.43 (8.20, 10.87) |
| New Zealand | 5.50 (4.75, 6.52) | 6.20 (5.37, 7.22) |
| Nicaragua | 0.08 (0.06, 0.10) | 0.13 (0.11, 0.16) |
| Niger | 0.15 (0.13, 0.19) | 0.17 (0.14, 0.20) |
| Nigeria | 0.22 (0.19, 0.27) | 0.26 (0.22, 0.31) |
| Niue | 0.17 (0.14, 0.20) | 0.21 (0.17, 0.25) |
| North Macedonia | 1.64 (1.34, 1.98) | 2.17 (1.83, 2.62) |
| Northern Mariana Islands | 0.19 (0.16, 0.24) | 0.23 (0.19, 0.29) |
| Norway | 6.50 (5.61, 7.52) | 8.26 (7.22, 9.46) |
| Oman | 0.51 (0.42, 0.62) | 0.97 (0.80, 1.20) |
| Pakistan | 0.60 (0.51, 0.73) | 0.73 (0.62, 0.89) |
| Palau | 0.17 (0.14, 0.20) | 0.23 (0.19, 0.28) |
| Palestine | 0.41 (0.34, 0.50) | 0.66 (0.54, 0.80) |
| Panama | 0.18 (0.14, 0.22) | 0.25 (0.21, 0.31) |
| Papua New Guinea | 0.10 (0.08, 0.13) | 0.11 (0.09, 0.14) |
| Paraguay | 0.31 (0.25, 0.37) | 0.33 (0.28, 0.40) |
| Peru | 0.26 (0.22, 0.31) | 0.36 (0.30, 0.43) |
| Philippines | 0.09 (0.08, 0.11) | 0.10 (0.09, 0.13) |
| Poland | 0.80 (0.68, 0.97) | 0.96 (0.82, 1.16) |
| Portugal | 2.05 (1.73, 2.45) | 2.72 (2.32, 3.23) |
| Puerto Rico | 0.78 (0.65, 0.93) | 1.07 (0.92, 1.30) |
| Qatar | 0.82 (0.67, 1.04) | 1.30 (1.07, 1.60) |
| Republic of Korea | 1.02 (0.87, 1.22) | 2.20 (1.87, 2.63) |
| Republic of Moldova | 0.96 (0.79, 1.18) | 1.25 (1.03, 1.53) |
| Romania | 0.45 (0.38, 0.54) | 0.53 (0.45, 0.64) |
| Russian Federation | 1.08 (0.92, 1.32) | 1.26 (1.06, 1.52) |
| Rwanda | 0.18 (0.15, 0.22) | 0.25 (0.21, 0.30) |
| Saint Kitts and Nevis | 0.59 (0.50, 0.72) | 0.94 (0.78, 1.14) |
| Saint Lucia | 0.50 (0.41, 0.60) | 0.92 (0.77, 1.13) |
| Saint Vincent and the Grenadines | 0.48 (0.40, 0.58) | 0.76 (0.63, 0.91) |
| Samoa | 0.12 (0.10, 0.14) | 0.14 (0.12, 0.17) |
| San Marino | 7.82 (6.67, 9.42) | 9.56 (8.23, 11.33) |
| Sao Tome and Principe | 0.24 (0.20, 0.28) | 0.34 (0.29, 0.42) |
| Saudi Arabia | 0.42 (0.35, 0.52) | 0.83 (0.68, 1.02) |
| Senegal | 0.18 (0.15, 0.22) | 0.26 (0.22, 0.32) |
| Serbia | 1.91 (1.60, 2.32) | 2.23 (1.89, 2.70) |
| Seychelles | 0.14 (0.12, 0.17) | 0.19 (0.16, 0.24) |
| Sierra Leone | 0.19 (0.16, 0.23) | 0.23 (0.19, 0.28) |
| Singapore | 0.21 (0.18, 0.26) | 0.30 (0.25, 0.36) |
| Slovakia | 1.93 (1.63, 2.33) | 2.37 (2.00, 2.92) |
| Slovenia | 2.91 (2.45, 3.49) | 3.54 (2.99, 4.22) |
| Solomon Islands | 0.08 (0.07, 0.10) | 0.10 (0.08, 0.13) |
| Somalia | 0.15 (0.12, 0.18) | 0.15 (0.12, 0.18) |
| South Africa | 0.27 (0.23, 0.33) | 0.44 (0.37, 0.53) |
| South Sudan | 0.18 (0.15, 0.22) | 0.19 (0.16, 0.24) |
| Spain | 3.17 (2.86, 3.54) | 3.86 (3.28, 4.61) |
| Sri Lanka | 0.18 (0.15, 0.22) | 0.24 (0.21, 0.30) |
| Sudan | 0.39 (0.32, 0.47) | 0.58 (0.47, 0.71) |
| Suriname | 0.56 (0.47, 0.68) | 0.76 (0.63, 0.95) |
| Sweden | 7.48 (6.54, 8.46) | 7.76 (6.90, 8.82) |
| Switzerland | 4.57 (3.90, 5.54) | 5.02 (4.28, 6.01) |
| Syrian Arab Republic | 0.42 (0.35, 0.51) | 0.91 (0.76, 1.11) |
| Taiwan (Province of China) | 0.17 (0.14, 0.21) | 0.23 (0.19, 0.28) |
| Tajikistan | 0.68 (0.56, 0.82) | 0.89 (0.74, 1.06) |
| Thailand | 0.12 (0.10, 0.14) | 0.19 (0.16, 0.23) |
| Timor-Leste | 0.09 (0.07, 0.11) | 0.11 (0.09, 0.13) |
| Togo | 0.19 (0.16, 0.23) | 0.26 (0.22, 0.31) |
| Tokelau | 0.13 (0.11, 0.16) | 0.18 (0.15, 0.22) |
| Tonga | 0.12 (0.10, 0.15) | 0.15 (0.13, 0.18) |
| Trinidad and Tobago | 0.63 (0.53, 0.77) | 0.96 (0.81, 1.17) |
| Tunisia | 0.58 (0.48, 0.71) | 1.14 (0.95, 1.36) |
| Türkiye | 0.58 (0.48, 0.71) | 1.14 (0.96, 1.36) |
| Turkmenistan | 0.73 (0.60, 0.88) | 1.06 (0.88, 1.28) |
| Tuvalu | 0.12 (0.10, 0.15) | 0.14 (0.11, 0.16) |
| Uganda | 0.15 (0.13, 0.18) | 0.19 (0.16, 0.23) |
| Ukraine | 1.09 (0.92, 1.30) | 1.08 (0.93, 1.31) |
| United Arab Emirate | 0.77 (0.63, 0.97) | 1.49 (1.20, 1.84) |
| United Kingdom | 4.06 (3.52, 4.81) | 5.03 (4.38, 5.80) |
| United Republic of Tanzania | 0.19 (0.16, 0.22) | 0.24 (0.20, 0.29) |
| United States of America | 5.33 (4.61, 6.23) | 6.39 (5.62, 7.30) |
| United States Virgin Islands | 0.82 (0.68, 0.99) | 1.20 (1.00, 1.45) |
| Uruguay | 1.48 (1.26, 1.81) | 1.60 (1.35, 1.94) |
| Uzbekistan | 0.76 (0.63, 0.92) | 1.05 (0.89, 1.29) |
| Vanuatu | 0.09 (0.08, 0.11) | 0.11 (0.09, 0.14) |
| Venezuela (Bolivarian Republic of) | 0.19 (0.16, 0.22) | 0.24 (0.19, 0.29) |
| Viet Nam | 0.11 (0.09, 0.14) | 0.17 (0.14, 0.21) |
| Yemen | 0.32 (0.26, 0.38) | 0.51 (0.42, 0.63) |
| Zambia | 0.17 (0.14, 0.20) | 0.22 (0.19, 0.27) |
| Zimbabwe | 0.18 (0.15, 0.22) | 0.24 (0.20, 0.30) |

Numbers in parenthesis represent 95% uncertainty intervals (UIs). IBD=inflammatory bowel disease. MASLD=metabolic dysfunction-associated steatotic liver disease. PAF=population attributable fraction. SDI=socio-demographic index.

**Table S4.** Age-standardized prevalence, DALYs, and mortality rates (per 10,000,000 individuals) due to MASLD–IBD comorbidity in 1990 and 2021 and EAPCs from 1990 to 2021, stratified by country

| **Country** | **Age-standardized prevalence rate** | | | **Age-standardized DALYs rate** | | | **Age-standardized mortality rate** | | |
| --- | --- | --- | --- | --- | --- | --- | --- | --- | --- |
|  | **1990 No. (95% UI)** | **2021 No. (95% UI)** | **EAPC % (95% CI)** | **1990 No. (95% UI)** | **2021 No. (95% UI)** | **EAPC % (95% CI)** | **1990 ‱ (95% UI)** | **2021 ‱ (95% UI)** | **EAPC % (95% CI)** |
| Afghanistan | 94.8 (86.9, 104.3) | 133.5 (122.0, 146.4) | 1.44 (1.25, 1.63) | 0.3 (0.2, 0.4) | 0.3 (0.2, 0.4) | 0.33 (0.13, 0.53) | 1.2 (0.7, 2.2) | 1.3 (0.8, 2.0) | 0.37 (0.17, 0.57) |
| Albania | 241.3 (220.4, 266.4) | 256.9 (235.0, 280.2) | 0.09 (-0.01, 0.18) | 0.7 (0.5, 1.0) | 0.5 (0.4, 0.7) | -0.98 (-1.48, -0.47) | 3.1 (2.2, 4.3) | 2.3 (1.6, 3.5) | -1.07 (-1.62, -0.51) |
| Algeria | 163.6 (149.9, 178.2) | 255.8 (236.0, 277.2) | 1.79 (1.59, 1.99) | 0.2 (0.1, 0.2) | 0.3 (0.2, 0.4) | 2.23 (1.96, 2.49) | 0.8 (0.5, 1.2) | 1.4 (0.9, 2.1) | 2.47 (2.18, 2.75) |
| American Samoa | 33.9 (31.0, 36.8) | 39.3 (36.1, 42.6) | 0.46 (0.40, 0.51) | 0.1 (0.1, 0.1) | 0.1 (0.1, 0.2) | 0.61 (0.44, 0.79) | 0.3 (0.2, 0.5) | 0.5 (0.3, 0.6) | 0.73 (0.55, 0.91) |
| Andorra | 315.5 (289.8, 346.0) | 423.2 (387.3, 461.9) | 1.02 (0.99, 1.05) | 2.6 (1.6, 3.7) | 2.2 (1.4, 3.2) | -0.13 (-0.30, 0.03) | 10.2 (6.4, 14.7) | 9.1 (5.8, 12.9) | -0.01 (-0.19, 0.17) |
| Angola | 32.0 (29.0, 35.2) | 38.8 (35.3, 42.6) | 0.57 (0.52, 0.63) | 0.2 (0.1, 0.3) | 0.2 (0.1, 0.3) | -0.27 (-0.49, -0.05) | 0.7 (0.4, 1.3) | 0.7 (0.4, 1.2) | -0.25 (-0.47, -0.03) |
| Antigua and Barbuda | 120.5 (109.9, 132.0) | 130.7 (119.6, 143.0) | 0.30 (0.28, 0.32) | 0.4 (0.3, 0.6) | 0.5 (0.3, 0.6) | -0.04 (-0.49, 0.42) | 1.7 (1.3, 2.4) | 1.9 (1.4, 2.5) | 0.06 (-0.38, 0.50) |
| Argentina | 94.6 (86.2, 104.3) | 129.4 (118.1, 141.9) | 1.07 (1.03, 1.12) | 0.4 (0.3, 0.6) | 0.4 (0.3, 0.6) | 0.34 (0.12, 0.56) | 1.7 (1.2, 2.3) | 1.6 (1.1, 2.2) | 0.39 (0.16, 0.62) |
| Armenia | 180.8 (165.0, 199.3) | 220.0 (200.7, 240.2) | 0.78 (0.73, 0.83) | 0.5 (0.3, 0.6) | 0.7 (0.5, 0.9) | 2.10 (1.30, 2.91) | 1.8 (1.4, 2.4) | 3.0 (2.2, 3.9) | 2.69 (1.74, 3.64) |
| Australia | 355.1 (324.3, 387.4) | 493.6 (451.2, 540.8) | 1.53 (1.19, 1.88) | 1.1 (0.8, 1.5) | 1.8 (1.5, 2.3) | 2.41 (2.04, 2.78) | 4.0 (3.0, 5.2) | 7.0 (5.6, 8.7) | 2.56 (2.20, 2.91) |
| Austria | 240.8 (218.3, 264.3) | 364.6 (333.5, 398.6) | 1.27 (1.18, 1.35) | 2.2 (1.5, 3.0) | 1.7 (1.2, 2.2) | -1.03 (-1.28, -0.77) | 7.7 (5.5, 10.4) | 6.7 (4.8, 8.6) | -0.61 (-0.87, -0.36) |
| Azerbaijan | 175.4 (159.7, 192.9) | 224.3 (205.6, 246.7) | 1.05 (0.82, 1.27) | 0.8 (0.5, 1.0) | 1.2 (0.8, 1.6) | 1.40 (1.14, 1.65) | 3.3 (2.3, 4.4) | 5.0 (3.2, 7.5) | 1.65 (1.41, 1.90) |
| Bahamas | 114.3 (104.2, 125.0) | 122.9 (111.8, 134.1) | 0.35 (0.28, 0.41) | 0.9 (0.6, 1.2) | 0.7 (0.5, 1.0) | -0.63 (-0.91, -0.34) | 3.1 (2.3, 4.2) | 2.5 (1.8, 3.5) | -0.61 (-0.89, -0.33) |
| Bahrain | 174.2 (160.4, 190.0) | 245.0 (226.8, 264.7) | 1.57 (1.40, 1.74) | 0.6 (0.4, 0.8) | 0.6 (0.4, 0.9) | -0.22 (-0.46, 0.02) | 3.2 (2.2, 4.5) | 3.5 (2.4, 5.0) | 0.11 (-0.18, 0.40) |
| Bangladesh | 137.5 (124.7, 151.3) | 187.1 (170.5, 205.2) | 1.25 (1.16, 1.35) | 0.3 (0.2, 0.5) | 0.3 (0.2, 0.5) | 0.09 (-0.07, 0.24) | 1.2 (0.8, 1.8) | 1.3 (0.9, 1.9) | 0.29 (0.11, 0.46) |
| Barbados | 126.9 (116.0, 139.2) | 140.0 (127.7, 152.7) | 0.35 (0.21, 0.49) | 0.6 (0.4, 0.8) | 0.4 (0.3, 0.6) | -1.11 (-1.38, -0.84) | 2.1 (1.5, 2.7) | 1.6 (1.1, 2.3) | -0.96 (-1.23, -0.69) |
| Belarus | 112.6 (101.9, 124.0) | 125.3 (114.7, 136.3) | 0.44 (0.24, 0.64) | 0.2 (0.1, 0.2) | 0.5 (0.4, 0.8) | 3.93 (2.98, 4.90) | 0.7 (0.5, 0.9) | 1.7 (1.1, 2.4) | 3.49 (2.68, 4.31) |
| Belgium | 184.4 (167.6, 201.5) | 298.9 (273.6, 325.7) | 1.38 (1.27, 1.49) | 1.4 (1.0, 1.9) | 1.7 (1.3, 2.2) | 0.39 (0.10, 0.69) | 5.4 (4.0, 7.1) | 6.3 (4.8, 8.0) | 0.42 (0.20, 0.65) |
| Belize | 94.2 (85.8, 103.2) | 114.5 (104.5, 124.5) | 0.72 (0.64, 0.80) | 0.4 (0.3, 0.6) | 0.8 (0.5, 1.0) | 1.81 (1.39, 2.23) | 1.6 (1.2, 2.2) | 2.8 (2.0, 3.7) | 1.56 (1.13, 1.99) |
| Benin | 37.5 (34.0, 41.3) | 53.8 (49.1, 59.0) | 1.29 (1.23, 1.36) | 0.3 (0.2, 0.4) | 0.3 (0.2, 0.4) | 0.39 (0.26, 0.52) | 1.0 (0.6, 1.6) | 1.2 (0.8, 1.7) | 0.49 (0.35, 0.63) |
| Bermuda | 132.1 (120.6, 144.1) | 152.4 (140.2, 166.8) | 0.52 (0.47, 0.57) | 0.7 (0.6, 1.0) | 0.4 (0.3, 0.6) | -1.71 (-2.41, -1.00) | 3.0 (2.2, 3.8) | 1.7 (1.2, 2.3) | -1.87 (-2.57, -1.16) |
| Bhutan | 123.9 (112.0, 135.2) | 171.3 (156.4, 186.8) | 1.16 (1.09, 1.23) | 0.4 (0.2, 0.6) | 0.5 (0.4, 0.8) | 1.48 (1.40, 1.57) | 1.3 (0.8, 2.2) | 2.2 (1.5, 3.1) | 1.78 (1.70, 1.87) |
| Bolivia (Plurinational State of) | 32.0 (29.2, 35.2) | 41.1 (37.7, 45.0) | 0.78 (0.74, 0.83) | 0.4 (0.2, 0.7) | 0.5 (0.3, 0.8) | 0.67 (0.61, 0.74) | 1.6 (0.8, 2.6) | 2.3 (1.3, 3.6) | 0.99 (0.95, 1.03) |
| Bosnia and Herzegovina | 188.3 (171.4, 206.4) | 239.4 (219.2, 261.1) | 0.83 (0.76, 0.89) | 0.5 (0.4, 0.6) | 0.5 (0.4, 0.7) | 0.25 (0.10, 0.40) | 1.8 (1.3, 2.4) | 2.2 (1.5, 3.0) | 0.46 (0.31, 0.62) |
| Botswana | 42.3 (38.5, 46.4) | 59.3 (54.0, 65.1) | 1.00 (0.96, 1.04) | 0.2 (0.1, 0.3) | 0.2 (0.1, 0.3) | 0.54 (0.29, 0.79) | 0.6 (0.4, 1.1) | 0.8 (0.5, 1.2) | 0.61 (0.39, 0.83) |
| Brazil | 48.8 (44.8, 53.4) | 87.6 (80.1, 95.7) | 1.56 (1.06, 2.06) | 0.1 (0.1, 0.2) | 0.2 (0.2, 0.3) | 1.68 (1.16, 2.20) | 0.4 (0.3, 0.5) | 0.7 (0.5, 0.9) | 1.91 (1.41, 2.43) |
| Brunei Darussalam | 26.1 (24.0, 28.8) | 35.4 (32.4, 38.6) | 1.01 (0.96, 1.07) | 0.1 (0.1, 0.1) | 0.1 (0.1, 0.1) | -0.63 (-0.79, -0.47) | 0.4 (0.3, 0.5) | 0.3 (0.2, 0.4) | -0.42 (-0.57, -0.27) |
| Bulgaria | 194.8 (178.1, 212.9) | 202.3 (185.3, 221.7) | 0.17 (0.06, 0.28) | 0.7 (0.6, 1.0) | 0.9 (0.6, 1.2) | 0.46 (0.14, 0.78) | 2.9 (2.1, 3.8) | 2.9 (2.0, 4.1) | 0.07 (-0.25, 0.38) |
| Burkina Faso | 24.6 (22.2, 27.2) | 35.2 (32.1, 38.6) | 1.17 (1.14, 1.21) | 0.2 (0.1, 0.4) | 0.3 (0.2, 0.4) | 0.53 (0.46, 0.61) | 0.9 (0.5, 1.7) | 1.1 (0.6, 1.8) | 0.55 (0.47, 0.62) |
| Burundi | 33.7 (30.8, 37.3) | 37.1 (33.7, 40.8) | 0.02 (-0.11, 0.15) | 0.2 (0.1, 0.2) | 0.1 (0.1, 0.2) | -1.85 (-2.13, -1.57) | 0.6 (0.4, 1.0) | 0.5 (0.3, 0.7) | -1.69 (-1.96, -1.43) |
| Cabo Verde | 51.2 (46.7, 56.2) | 73.9 (67.5, 81.1) | 1.27 (1.22, 1.32) | 0.2 (0.1, 0.3) | 0.4 (0.2, 0.5) | 1.67 (1.54, 1.79) | 0.8 (0.5, 1.3) | 1.6 (1.0, 2.3) | 1.94 (1.77, 2.12) |
| Cambodia | 14.5 (13.2, 15.9) | 14.8 (13.5, 16.2) | 0.21 (0.17, 0.26) | 0.1 (0.0, 0.1) | 0.1 (0.0, 0.1) | -0.88 (-0.93, -0.83) | 0.3 (0.2, 0.6) | 0.3 (0.2, 0.4) | -0.69 (-0.75, -0.64) |
| Cameroon | 42.9 (39.0, 47.2) | 54.0 (49.3, 59.1) | 0.80 (0.68, 0.92) | 0.3 (0.2, 0.4) | 0.3 (0.2, 0.5) | -0.06 (-0.25, 0.12) | 1.3 (0.9, 1.8) | 1.4 (0.8, 2.0) | -0.04 (-0.23, 0.15) |
| Canada | 699.3 (634.2, 764.0) | 706.2 (643.6, 773.8) | 0.08 (-0.28, 0.44) | 3.1 (2.3, 4.2) | 4.6 (3.5, 5.7) | 1.55 (1.17, 1.93) | 11.9 (8.8, 15.8) | 17.7 (13.8, 22.3) | 1.59 (1.21, 1.97) |
| Central African Republic | 26.2 (23.9, 28.9) | 30.4 (27.6, 33.4) | 0.38 (0.35, 0.42) | 0.2 (0.1, 0.2) | 0.2 (0.1, 0.3) | -0.37 (-0.51, -0.23) | 0.6 (0.4, 1.0) | 0.6 (0.4, 1.0) | -0.36 (-0.50, -0.23) |
| Chad | 32.1 (29.2, 35.4) | 42.1 (38.2, 46.6) | 0.99 (0.92, 1.06) | 0.2 (0.1, 0.3) | 0.2 (0.1, 0.4) | 0.71 (0.57, 0.84) | 0.7 (0.4, 1.3) | 0.9 (0.6, 1.5) | 0.72 (0.59, 0.85) |
| Chile | 110.3 (100.3, 121.1) | 156.2 (142.2, 170.5) | 1.13 (1.03, 1.23) | 1.3 (0.9, 1.8) | 1.0 (0.8, 1.4) | -0.15 (-0.43, 0.12) | 4.4 (3.0, 5.9) | 4.0 (2.9, 5.4) | 0.15 (-0.15, 0.44) |
| China | 18.2 (16.6, 20.0) | 36.4 (33.3, 39.7) | 3.23 (2.64, 3.83) | 0.0 (0.0, 0.0) | 0.0 (0.0, 0.1) | 1.54 (0.97, 2.12) | 0.1 (0.1, 0.2) | 0.2 (0.1, 0.2) | 1.94 (1.33, 2.56) |
| Colombia | 43.7 (39.8, 47.9) | 54.7 (49.8, 59.8) | 0.67 (0.42, 0.92) | 0.1 (0.1, 0.1) | 0.1 (0.1, 0.2) | 0.09 (-0.20, 0.39) | 0.4 (0.3, 0.5) | 0.5 (0.3, 0.7) | 0.36 (0.06, 0.65) |
| Comoros | 52.3 (47.7, 57.4) | 61.7 (56.0, 67.4) | 0.57 (0.40, 0.75) | 0.2 (0.1, 0.3) | 0.2 (0.1, 0.3) | 0.01 (-0.12, 0.14) | 0.8 (0.6, 1.2) | 0.9 (0.6, 1.3) | 0.12 (-0.01, 0.25) |
| Congo | 38.4 (35.0, 42.2) | 46.2 (42.0, 50.5) | 0.64 (0.54, 0.73) | 0.3 (0.2, 0.4) | 0.3 (0.2, 0.5) | -0.43 (-0.59, -0.26) | 1.1 (0.7, 1.7) | 1.0 (0.6, 1.7) | -0.37 (-0.52, -0.23) |
| Cook Islands | 31.8 (29.1, 34.8) | 34.1 (31.4, 37.3) | 0.25 (0.16, 0.34) | 0.1 (0.0, 0.1) | 0.1 (0.0, 0.1) | 0.46 (0.26, 0.67) | 0.2 (0.2, 0.4) | 0.3 (0.2, 0.4) | 0.36 (0.12, 0.61) |
| Costa Rica | 24.1 (22.0, 26.2) | 29.3 (26.9, 32.0) | 0.57 (0.53, 0.62) | 0.1 (0.1, 0.1) | 0.2 (0.1, 0.2) | 0.83 (0.56, 1.11) | 0.4 (0.3, 0.5) | 0.7 (0.5, 0.9) | 0.96 (0.69, 1.24) |
| Côte d'Ivoire | 35.6 (32.3, 39.1) | 44.7 (40.7, 49.0) | 0.74 (0.65, 0.84) | 0.2 (0.1, 0.2) | 0.2 (0.1, 0.3) | 0.16 (0.08, 0.24) | 0.7 (0.5, 0.9) | 0.7 (0.4, 1.0) | 0.15 (0.07, 0.24) |
| Croatia | 210.0 (192.1, 230.8) | 260.3 (238.2, 285.4) | 0.49 (0.03, 0.96) | 0.9 (0.6, 1.2) | 0.7 (0.5, 0.9) | -0.94 (-1.48, -0.40) | 3.2 (2.3, 4.3) | 2.7 (2.0, 3.7) | -0.57 (-1.09, -0.04) |
| Cuba | 112.2 (102.4, 122.8) | 127.8 (117.3, 139.4) | 0.49 (0.42, 0.56) | 0.3 (0.2, 0.4) | 0.5 (0.4, 0.8) | 1.66 (1.12, 2.19) | 1.3 (1.0, 1.8) | 1.9 (1.4, 2.6) | 1.13 (0.58, 1.69) |
| Cyprus | 210.6 (190.8, 230.2) | 316.7 (288.6, 345.7) | 1.38 (1.31, 1.46) | 1.0 (0.7, 1.4) | 0.7 (0.5, 1.0) | -1.13 (-1.26, -1.00) | 4.5 (3.1, 6.3) | 3.2 (2.3, 4.2) | -1.31 (-1.43, -1.19) |
| Czechia | 501.4 (460.5, 549.3) | 585.7 (537.2, 638.1) | 1.00 (0.64, 1.36) | 1.8 (1.3, 2.4) | 1.9 (1.4, 2.7) | 0.96 (0.54, 1.38) | 6.4 (4.8, 8.5) | 6.8 (4.8, 9.2) | 0.81 (0.43, 1.20) |
| Democratic People's Republic of Korea | 20.5 (18.6, 22.4) | 22.1 (20.2, 24.2) | 0.43 (0.34, 0.51) | 0.0 (0.0, 0.1) | 0.0 (0.0, 0.0) | -0.93 (-0.98, -0.89) | 0.2 (0.1, 0.3) | 0.1 (0.1, 0.2) | -0.89 (-0.94, -0.83) |
| Democratic Republic of the Congo | 38.6 (35.0, 42.7) | 36.7 (33.5, 40.1) | -0.36 (-0.64, -0.08) | 0.1 (0.1, 0.2) | 0.1 (0.1, 0.2) | -1.14 (-1.46, -0.82) | 0.5 (0.3, 0.8) | 0.4 (0.3, 0.6) | -1.11 (-1.42, -0.80) |
| Denmark | 314.4 (287.8, 345.6) | 396.7 (361.9, 431.4) | 0.85 (0.52, 1.17) | 1.8 (1.3, 2.5) | 1.6 (1.2, 2.2) | -0.68 (-1.52, 0.17) | 5.7 (4.1, 7.7) | 6.2 (4.5, 8.0) | -0.12 (-0.93, 0.70) |
| Djibouti | 39.9 (36.3, 43.8) | 53.3 (48.3, 58.8) | 0.95 (0.86, 1.05) | 0.1 (0.1, 0.2) | 0.2 (0.1, 0.3) | 0.92 (0.84, 1.00) | 0.6 (0.4, 0.9) | 0.8 (0.5, 1.1) | 1.02 (0.95, 1.08) |
| Dominica | 102.9 (93.9, 112.4) | 120.8 (110.4, 132.0) | 0.64 (0.57, 0.72) | 0.5 (0.3, 0.7) | 0.6 (0.4, 0.8) | 0.62 (0.45, 0.78) | 2.0 (1.4, 2.8) | 2.4 (1.6, 3.2) | 0.45 (0.32, 0.58) |
| Dominican Republic | 85.1 (77.8, 93.1) | 98.8 (90.2, 107.8) | 0.59 (0.46, 0.72) | 0.6 (0.4, 0.8) | 0.7 (0.4, 1.1) | 0.99 (0.62, 1.35) | 2.4 (1.6, 3.3) | 2.7 (1.6, 4.3) | 0.86 (0.55, 1.17) |
| Ecuador | 55.1 (50.2, 60.2) | 66.8 (61.3, 72.9) | 0.72 (0.63, 0.81) | 0.5 (0.4, 0.6) | 0.6 (0.4, 0.7) | 0.66 (0.43, 0.89) | 1.9 (1.4, 2.5) | 2.4 (1.7, 3.2) | 1.13 (0.88, 1.39) |
| Egypt | 221.2 (202.8, 239.9) | 327.2 (302.4, 353.6) | 1.68 (1.49, 1.87) | 1.6 (1.1, 2.2) | 2.0 (1.4, 2.8) | 1.35 (1.11, 1.58) | 9.0 (5.6, 13.6) | 9.8 (6.7, 13.6) | 0.75 (0.56, 0.94) |
| El Salvador | 17.5 (15.9, 19.3) | 25.8 (23.5, 28.2) | 1.38 (1.27, 1.49) | 0.1 (0.1, 0.2) | 0.2 (0.1, 0.3) | 1.88 (1.72, 2.03) | 0.4 (0.3, 0.5) | 0.7 (0.4, 0.9) | 1.91 (1.77, 2.06) |
| Equatorial Guinea | 32.4 (29.7, 35.5) | 55.1 (50.5, 60.5) | 1.92 (1.70, 2.14) | 0.1 (0.1, 0.2) | 0.2 (0.1, 0.4) | 2.55 (2.40, 2.70) | 0.4 (0.3, 0.7) | 0.9 (0.6, 1.5) | 2.80 (2.66, 2.94) |
| Eritrea | 35.3 (31.9, 38.9) | 40.6 (37.0, 44.7) | 0.16 (0.05, 0.27) | 0.2 (0.1, 0.2) | 0.2 (0.1, 0.2) | -0.41 (-0.58, -0.23) | 0.6 (0.4, 0.9) | 0.7 (0.4, 0.9) | -0.27 (-0.44, -0.09) |
| Estonia | 105.3 (96.2, 115.5) | 119.8 (109.3, 130.7) | 0.69 (0.50, 0.88) | 0.3 (0.2, 0.3) | 0.6 (0.4, 0.9) | 2.81 (2.30, 3.33) | 0.9 (0.6, 1.1) | 2.0 (1.4, 2.8) | 2.73 (2.30, 3.17) |
| Eswatini | 44.4 (40.4, 48.7) | 60.0 (54.6, 65.8) | 0.87 (0.79, 0.95) | 0.3 (0.2, 0.4) | 0.6 (0.3, 0.9) | 2.30 (1.37, 3.23) | 1.1 (0.7, 1.8) | 2.1 (1.2, 3.3) | 2.08 (1.23, 2.93) |
| Ethiopia | 29.5 (26.8, 32.3) | 38.8 (35.5, 42.5) | 0.63 (0.44, 0.82) | 0.1 (0.1, 0.2) | 0.1 (0.1, 0.1) | -1.25 (-1.59, -0.92) | 0.5 (0.3, 0.7) | 0.5 (0.3, 0.6) | -1.08 (-1.42, -0.74) |
| Fiji | 28.0 (25.6, 30.7) | 32.3 (29.5, 35.3) | 0.48 (0.43, 0.52) | 0.0 (0.0, 0.1) | 0.1 (0.0, 0.1) | 0.67 (0.61, 0.74) | 0.1 (0.1, 0.2) | 0.2 (0.1, 0.3) | 0.80 (0.73, 0.87) |
| Finland | 272.9 (248.6, 298.3) | 401.1 (365.6, 440.5) | 0.52 (-0.01, 1.06) | 1.7 (1.3, 2.3) | 2.7 (2.0, 3.7) | 1.34 (0.55, 2.13) | 6.1 (4.5, 8.0) | 9.6 (7.1, 12.8) | 1.28 (0.61, 1.96) |
| France | 221.9 (202.1, 243.1) | 343.4 (313.1, 375.8) | 1.34 (1.16, 1.53) | 1.7 (1.2, 2.3) | 1.4 (1.0, 1.8) | -0.76 (-1.10, -0.41) | 6.1 (4.5, 8.2) | 5.5 (4.0, 7.1) | -0.44 (-0.77, -0.11) |
| Gabon | 40.0 (36.4, 44.1) | 53.6 (49.0, 59.1) | 0.82 (0.76, 0.87) | 0.3 (0.2, 0.4) | 0.4 (0.2, 0.5) | 1.07 (0.87, 1.27) | 0.9 (0.6, 1.5) | 1.4 (0.9, 2.1) | 1.12 (0.91, 1.33) |
| Gambia | 45.4 (41.1, 49.7) | 52.6 (48.3, 57.9) | 0.53 (0.45, 0.61) | 0.3 (0.2, 0.5) | 0.5 (0.3, 0.7) | 1.07 (0.91, 1.23) | 1.3 (0.9, 1.8) | 1.9 (1.1, 2.9) | 1.10 (0.98, 1.22) |
| Georgia | 198.0 (181.1, 217.7) | 212.9 (194.6, 231.7) | 0.40 (0.29, 0.51) | 0.7 (0.5, 1.0) | 0.7 (0.5, 1.0) | 0.61 (0.23, 1.00) | 2.7 (2.0, 3.7) | 2.7 (1.9, 3.7) | 0.43 (-0.01, 0.87) |
| Germany | 364.7 (330.5, 398.5) | 602.4 (550.2, 657.4) | 1.75 (1.33, 2.17) | 3.7 (2.6, 5.0) | 3.7 (2.7, 4.8) | -0.24 (-0.68, 0.21) | 13.3 (9.5, 17.6) | 14.1 (10.6, 18.0) | 0.05 (-0.37, 0.48) |
| Ghana | 45.1 (41.0, 49.6) | 66.5 (60.5, 73.0) | 1.14 (1.10, 1.18) | 0.2 (0.1, 0.4) | 0.4 (0.2, 0.6) | 1.45 (1.28, 1.62) | 0.9 (0.6, 1.5) | 1.7 (1.1, 2.3) | 1.67 (1.48, 1.85) |
| Greece | 68.2 (62.4, 74.8) | 107.9 (98.5, 117.3) | 1.41 (0.82, 2.00) | 0.3 (0.2, 0.4) | 0.3 (0.2, 0.4) | 0.53 (-0.11, 1.17) | 1.2 (0.9, 1.6) | 1.3 (1.0, 1.6) | 0.03 (-0.56, 0.62) |
| Greenland | 427.5 (389.9, 467.5) | 478.7 (434.6, 522.0) | 0.44 (0.41, 0.47) | 3.0 (2.2, 4.0) | 2.9 (2.1, 4.0) | 0.28 (0.16, 0.39) | 9.6 (7.3, 13.0) | 9.9 (7.2, 13.2) | 0.42 (0.29, 0.55) |
| Grenada | 80.6 (74.0, 88.4) | 101.0 (92.4, 110.6) | 0.74 (0.72, 0.77) | 0.4 (0.3, 0.5) | 0.5 (0.3, 0.6) | 0.78 (0.51, 1.06) | 1.3 (0.9, 1.8) | 1.8 (1.3, 2.5) | 0.95 (0.68, 1.23) |
| Guam | 30.1 (27.5, 32.9) | 33.8 (31.0, 36.8) | 0.41 (0.35, 0.47) | 0.1 (0.1, 0.1) | 0.1 (0.1, 0.2) | 1.20 (0.97, 1.42) | 0.4 (0.3, 0.5) | 0.4 (0.3, 0.5) | 0.70 (0.43, 0.97) |
| Guatemala | 17.2 (15.7, 18.9) | 22.5 (20.5, 24.6) | 0.89 (0.87, 0.91) | 0.2 (0.1, 0.2) | 0.2 (0.1, 0.3) | 0.80 (0.44, 1.17) | 0.5 (0.4, 0.7) | 0.7 (0.5, 1.0) | 0.85 (0.51, 1.19) |
| Guinea | 41.2 (37.5, 45.0) | 54.2 (49.4, 59.7) | 0.97 (0.90, 1.05) | 0.3 (0.2, 0.4) | 0.3 (0.2, 0.5) | 0.71 (0.57, 0.86) | 1.1 (0.8, 1.6) | 1.3 (0.9, 1.8) | 0.73 (0.58, 0.88) |
| Guinea-Bissau | 35.5 (32.4, 38.8) | 46.6 (42.2, 51.0) | 0.93 (0.89, 0.98) | 0.3 (0.2, 0.5) | 0.3 (0.2, 0.5) | -0.19 (-0.27, -0.12) | 1.4 (0.8, 2.0) | 1.4 (1.0, 2.0) | -0.05 (-0.13, 0.02) |
| Guyana | 78.7 (71.6, 86.8) | 92.9 (85.0, 101.8) | 0.54 (0.45, 0.62) | 0.8 (0.5, 1.1) | 1.0 (0.6, 1.4) | 1.06 (0.76, 1.36) | 2.9 (2.0, 3.9) | 3.2 (2.1, 4.8) | 0.87 (0.55, 1.18) |
| Haiti | 66.3 (60.2, 72.9) | 73.2 (66.7, 80.1) | 0.38 (0.33, 0.43) | 0.5 (0.3, 0.7) | 0.4 (0.2, 0.6) | -0.65 (-0.77, -0.52) | 1.9 (1.1, 2.9) | 1.5 (0.8, 2.4) | -0.62 (-0.74, -0.50) |
| Honduras | 19.0 (17.2, 20.9) | 24.2 (22.0, 26.4) | 0.75 (0.73, 0.77) | 0.1 (0.1, 0.2) | 0.2 (0.1, 0.3) | 1.21 (1.09, 1.32) | 0.4 (0.3, 0.6) | 0.7 (0.3, 1.0) | 1.59 (1.46, 1.72) |
| Hungary | 466.6 (426.3, 510.8) | 535.6 (488.8, 587.5) | 0.26 (-0.02, 0.54) | 3.3 (2.3, 4.5) | 1.9 (1.3, 2.8) | -3.29 (-3.91, -2.67) | 10.1 (7.3, 13.9) | 6.6 (4.5, 9.5) | -2.71 (-3.27, -2.15) |
| Iceland | 487.9 (446.4, 532.5) | 530.3 (487.8, 577.6) | 0.09 (-0.05, 0.22) | 0.8 (0.6, 1.1) | 0.8 (0.6, 1.0) | -0.60 (-0.84, -0.35) | 3.0 (2.3, 4.0) | 3.2 (2.4, 4.2) | -0.29 (-0.54, -0.04) |
| India | 120.1 (109.6, 131.5) | 166.0 (151.7, 180.9) | 1.20 (1.07, 1.33) | 0.3 (0.2, 0.4) | 0.4 (0.3, 0.6) | 1.16 (1.04, 1.28) | 1.1 (0.8, 1.5) | 1.6 (1.1, 2.2) | 1.43 (1.31, 1.56) |
| Indonesia | 22.1 (20.2, 24.3) | 25.1 (22.9, 27.4) | 0.40 (0.34, 0.47) | 0.1 (0.0, 0.1) | 0.1 (0.1, 0.1) | 0.63 (0.57, 0.70) | 0.3 (0.2, 0.5) | 0.4 (0.3, 0.5) | 1.01 (0.94, 1.09) |
| Iran (Islamic Republic of) | 196.6 (180.0, 213.9) | 262.3 (242.6, 283.6) | 1.60 (1.08, 2.12) | 0.4 (0.3, 0.6) | 0.4 (0.3, 0.5) | 0.61 (0.32, 0.90) | 1.8 (1.2, 2.7) | 1.9 (1.4, 2.5) | 0.67 (0.39, 0.96) |
| Iraq | 109.0 (99.8, 118.8) | 182.2 (167.6, 197.2) | 1.81 (1.67, 1.95) | 0.2 (0.1, 0.3) | 0.3 (0.2, 0.4) | 1.16 (0.88, 1.43) | 0.8 (0.5, 1.2) | 1.2 (0.8, 1.7) | 1.43 (1.12, 1.74) |
| Ireland | 329.4 (300.8, 359.0) | 469.7 (429.8, 513.5) | 1.41 (1.24, 1.59) | 0.8 (0.6, 1.1) | 1.2 (0.9, 1.5) | 1.63 (1.02, 2.24) | 3.2 (2.4, 4.2) | 4.4 (3.3, 5.6) | 1.47 (0.96, 1.99) |
| Israel | 340.3 (310.6, 373.0) | 475.8 (437.2, 518.7) | 0.38 (-0.24, 0.99) | 1.1 (0.8, 1.5) | 0.9 (0.7, 1.2) | -1.61 (-2.39, -0.82) | 4.7 (3.4, 6.3) | 3.9 (2.9, 5.0) | -1.61 (-2.40, -0.82) |
| Italy | 299.1 (273.2, 327.2) | 322.8 (296.5, 351.8) | 0.42 (0.26, 0.58) | 2.5 (1.9, 3.2) | 0.7 (0.6, 0.9) | -4.02 (-4.19, -3.84) | 10.0 (7.5, 12.8) | 3.3 (2.5, 4.1) | -3.64 (-3.78, -3.50) |
| Jamaica | 105.6 (95.7, 115.4) | 132.5 (121.3, 144.7) | 0.77 (0.74, 0.81) | 0.2 (0.1, 0.3) | 0.2 (0.2, 0.3) | 0.45 (0.11, 0.78) | 0.8 (0.6, 1.0) | 0.9 (0.6, 1.3) | 0.50 (0.19, 0.81) |
| Japan | 49.7 (45.5, 53.9) | 58.8 (53.9, 63.9) | 0.18 (-0.31, 0.68) | 0.2 (0.2, 0.3) | 0.1 (0.1, 0.1) | -2.53 (-3.18, -1.87) | 0.9 (0.7, 1.1) | 0.6 (0.4, 0.7) | -2.09 (-2.74, -1.43) |
| Jordan | 231.2 (212.5, 253.0) | 327.6 (303.4, 354.2) | 1.62 (1.41, 1.82) | 0.3 (0.2, 0.5) | 0.3 (0.2, 0.4) | -0.30 (-0.63, 0.03) | 1.6 (1.0, 2.3) | 1.5 (1.0, 2.1) | -0.17 (-0.51, 0.16) |
| Kazakhstan | 155.0 (141.9, 170.4) | 185.6 (169.3, 203.4) | 0.59 (0.45, 0.73) | 0.6 (0.5, 0.8) | 1.5 (1.1, 2.1) | 3.26 (2.79, 3.73) | 2.4 (1.8, 3.1) | 5.8 (4.2, 7.9) | 3.45 (3.03, 3.86) |
| Kenya | 43.2 (39.4, 47.2) | 55.2 (50.3, 60.3) | 0.60 (0.53, 0.68) | 0.2 (0.1, 0.3) | 0.3 (0.2, 0.5) | 1.95 (1.83, 2.06) | 0.8 (0.4, 1.3) | 1.4 (0.9, 2.2) | 2.02 (1.93, 2.11) |
| Kiribati | 22.3 (20.5, 24.5) | 25.4 (23.2, 27.9) | 0.33 (0.25, 0.41) | 0.1 (0.1, 0.1) | 0.1 (0.1, 0.1) | -0.26 (-0.52, 0.01) | 0.3 (0.2, 0.4) | 0.3 (0.2, 0.4) | -0.18 (-0.45, 0.09) |
| Kuwait | 305.0 (280.7, 331.5) | 341.6 (316.6, 368.3) | 0.31 (-0.06, 0.68) | 0.3 (0.3, 0.5) | 0.3 (0.2, 0.4) | 0.05 (-0.45, 0.55) | 1.5 (1.1, 2.0) | 1.5 (1.0, 2.0) | 0.56 (0.07, 1.06) |
| Kyrgyzstan | 165.5 (151.2, 181.7) | 187.7 (171.7, 205.5) | 0.34 (0.22, 0.45) | 0.7 (0.5, 1.0) | 0.8 (0.6, 1.2) | 0.38 (-0.04, 0.80) | 2.7 (1.9, 3.7) | 2.8 (1.9, 3.9) | 0.16 (-0.20, 0.52) |
| Lao People's Democratic Republic | 12.3 (11.2, 13.5) | 15.1 (13.7, 16.6) | 0.84 (0.76, 0.92) | 0.1 (0.0, 0.1) | 0.0 (0.0, 0.1) | -0.78 (-0.83, -0.74) | 0.2 (0.1, 0.3) | 0.2 (0.1, 0.2) | -0.61 (-0.66, -0.56) |
| Latvia | 109.2 (100.0, 119.4) | 121.8 (111.6, 132.8) | 0.52 (0.36, 0.69) | 0.3 (0.2, 0.4) | 0.6 (0.4, 0.9) | 2.56 (1.99, 3.13) | 0.9 (0.7, 1.2) | 2.0 (1.4, 2.7) | 2.32 (1.83, 2.81) |
| Lebanon | 468.8 (431.4, 510.5) | 697.5 (644.1, 756.0) | 1.50 (1.39, 1.60) | 0.7 (0.5, 1.0) | 0.7 (0.5, 0.9) | 0.32 (0.07, 0.57) | 3.4 (2.2, 5.0) | 3.6 (2.5, 5.0) | 0.51 (0.28, 0.75) |
| Lesotho | 34.3 (31.3, 37.6) | 48.0 (43.7, 52.6) | 1.06 (1.01, 1.10) | 0.1 (0.1, 0.3) | 0.4 (0.2, 0.7) | 3.89 (3.31, 4.47) | 0.6 (0.3, 1.2) | 1.6 (0.9, 2.5) | 3.60 (3.05, 4.15) |
| Liberia | 39.6 (35.8, 43.6) | 52.8 (48.1, 57.8) | 1.37 (1.21, 1.54) | 0.3 (0.2, 0.4) | 0.4 (0.3, 0.5) | 1.12 (0.82, 1.43) | 1.3 (0.8, 1.8) | 1.5 (1.1, 2.1) | 1.17 (0.87, 1.46) |
| Libya | 109.0 (99.7, 118.8) | 207.4 (192.0, 224.7) | 2.54 (2.08, 3.01) | 0.2 (0.1, 0.4) | 0.5 (0.3, 0.8) | 3.76 (3.35, 4.18) | 1.0 (0.5, 2.1) | 2.3 (1.4, 3.8) | 3.81 (3.37, 4.25) |
| Lithuania | 124.4 (113.4, 137.3) | 141.5 (129.3, 154.6) | 0.89 (0.32, 1.45) | 0.3 (0.2, 0.4) | 0.8 (0.6, 1.2) | 4.39 (2.90, 5.90) | 0.9 (0.7, 1.2) | 2.6 (1.8, 3.6) | 4.19 (2.79, 5.61) |
| Luxembourg | 247.1 (225.3, 272.1) | 397.1 (362.3, 436.1) | 1.48 (1.39, 1.57) | 2.2 (1.5, 3.0) | 1.6 (1.1, 2.1) | -1.03 (-1.26, -0.81) | 7.9 (5.7, 10.4) | 6.4 (4.6, 8.4) | -0.69 (-0.89, -0.49) |
| Madagascar | 35.5 (32.5, 38.9) | 42.1 (38.2, 46.0) | 0.29 (0.13, 0.46) | 0.1 (0.1, 0.2) | 0.1 (0.1, 0.2) | -0.21 (-0.45, 0.02) | 0.5 (0.3, 0.7) | 0.5 (0.3, 0.8) | -0.14 (-0.37, 0.09) |
| Malawi | 35.6 (32.3, 39.0) | 43.8 (40.0, 48.2) | 0.50 (0.44, 0.57) | 0.2 (0.1, 0.2) | 0.2 (0.2, 0.3) | 0.47 (0.15, 0.78) | 0.7 (0.5, 0.9) | 0.9 (0.6, 1.3) | 0.54 (0.24, 0.83) |
| Malaysia | 29.0 (26.4, 31.8) | 41.1 (37.8, 44.8) | 1.80 (1.47, 2.12) | 0.0 (0.0, 0.0) | 0.1 (0.1, 0.1) | 2.65 (2.17, 3.14) | 0.1 (0.1, 0.2) | 0.3 (0.2, 0.4) | 2.83 (2.24, 3.42) |
| Maldives | 19.1 (17.4, 20.9) | 26.8 (24.5, 29.3) | 0.90 (0.81, 0.98) | 0.0 (0.0, 0.0) | 0.0 (0.0, 0.0) | -1.03 (-1.16, -0.89) | 0.1 (0.1, 0.2) | 0.1 (0.1, 0.2) | -0.52 (-0.63, -0.40) |
| Mali | 39.8 (36.1, 43.7) | 55.0 (50.0, 60.6) | 1.19 (1.11, 1.28) | 0.2 (0.2, 0.3) | 0.3 (0.2, 0.5) | 1.33 (1.17, 1.49) | 0.9 (0.7, 1.3) | 1.4 (1.0, 1.9) | 1.39 (1.26, 1.51) |
| Malta | 214.6 (195.3, 235.1) | 291.4 (265.3, 318.2) | 0.93 (0.87, 0.98) | 0.6 (0.5, 0.8) | 0.6 (0.4, 0.8) | -0.27 (-0.57, 0.04) | 2.4 (1.7, 3.2) | 2.3 (1.7, 3.1) | -0.42 (-0.73, -0.10) |
| Marshall Islands | 19.5 (17.8, 21.4) | 22.5 (20.5, 24.5) | 0.43 (0.38, 0.47) | 0.1 (0.0, 0.1) | 0.1 (0.0, 0.1) | 0.10 (-0.01, 0.22) | 0.2 (0.1, 0.3) | 0.2 (0.1, 0.3) | 0.13 (0.02, 0.24) |
| Mauritania | 56.1 (50.9, 61.9) | 87.4 (79.9, 95.6) | 1.22 (1.10, 1.35) | 0.5 (0.2, 1.0) | 0.5 (0.3, 0.8) | -0.42 (-0.55, -0.28) | 2.1 (1.0, 4.0) | 2.3 (1.5, 3.3) | -0.27 (-0.40, -0.13) |
| Mauritius | 19.8 (18.1, 21.7) | 25.3 (23.3, 27.7) | 0.91 (0.85, 0.97) | 0.0 (0.0, 0.1) | 0.0 (0.0, 0.0) | -1.09 (-1.30, -0.87) | 0.2 (0.1, 0.2) | 0.1 (0.1, 0.1) | -1.16 (-1.37, -0.95) |
| Mexico | 7.7 (7.0, 8.4) | 8.2 (7.5, 8.9) | 0.53 (0.28, 0.79) | 0.1 (0.1, 0.1) | 0.1 (0.1, 0.1) | 0.75 (0.50, 1.00) | 0.3 (0.2, 0.4) | 0.3 (0.3, 0.4) | 0.75 (0.50, 1.00) |
| Micronesia (Federated States of) | 21.0 (19.1, 23.1) | 24.2 (22.2, 26.5) | 0.35 (0.27, 0.42) | 0.1 (0.1, 0.1) | 0.1 (0.1, 0.1) | -0.19 (-0.43, 0.04) | 0.2 (0.2, 0.4) | 0.3 (0.2, 0.4) | -0.08 (-0.33, 0.16) |
| Monaco | 319.1 (291.7, 348.3) | 441.6 (404.3, 480.8) | 1.12 (1.08, 1.16) | 2.2 (1.5, 3.1) | 3.0 (2.2, 4.2) | 1.26 (0.98, 1.54) | 8.0 (5.6, 11.5) | 11.4 (8.3, 15.1) | 1.29 (1.01, 1.58) |
| Mongolia | 128.2 (117.3, 140.9) | 138.0 (125.5, 151.3) | 0.35 (0.31, 0.40) | 1.6 (1.1, 2.2) | 2.0 (1.4, 2.7) | 0.99 (0.63, 1.34) | 6.1 (4.4, 8.4) | 8.7 (6.2, 12.1) | 1.55 (1.11, 1.99) |
| Montenegro | 204.6 (185.5, 223.5) | 253.7 (232.5, 276.9) | 0.77 (0.71, 0.83) | 0.5 (0.3, 0.6) | 0.6 (0.4, 0.8) | 1.19 (1.12, 1.26) | 1.8 (1.3, 2.4) | 2.5 (1.8, 3.6) | 1.48 (1.39, 1.57) |
| Morocco | 132.3 (121.9, 145.1) | 212.1 (194.5, 230.2) | 1.81 (1.66, 1.97) | 0.1 (0.1, 0.2) | 0.2 (0.2, 0.4) | 2.44 (2.25, 2.64) | 0.6 (0.3, 1.0) | 1.1 (0.7, 1.7) | 2.58 (2.38, 2.79) |
| Mozambique | 28.6 (26.0, 31.4) | 35.2 (32.0, 38.5) | 0.50 (0.42, 0.58) | 0.2 (0.1, 0.2) | 0.3 (0.2, 0.5) | 1.47 (1.36, 1.57) | 0.8 (0.5, 1.2) | 1.3 (0.7, 2.2) | 1.48 (1.38, 1.57) |
| Myanmar | 16.7 (15.1, 18.3) | 19.9 (18.2, 21.8) | 0.82 (0.74, 0.90) | 0.0 (0.0, 0.1) | 0.0 (0.0, 0.0) | 0.03 (-0.03, 0.09) | 0.1 (0.1, 0.2) | 0.1 (0.1, 0.2) | 0.18 (0.12, 0.24) |
| Namibia | 42.3 (38.5, 46.4) | 54.1 (49.3, 59.7) | 0.69 (0.62, 0.77) | 0.1 (0.1, 0.3) | 0.2 (0.1, 0.3) | 0.63 (0.35, 0.91) | 0.6 (0.3, 1.0) | 0.8 (0.5, 1.1) | 0.65 (0.40, 0.90) |
| Nauru | 21.4 (19.5, 23.3) | 23.6 (21.6, 25.8) | 0.25 (0.11, 0.39) | 0.1 (0.1, 0.1) | 0.1 (0.0, 0.1) | -0.76 (-1.02, -0.50) | 0.3 (0.2, 0.5) | 0.3 (0.1, 0.4) | -0.87 (-1.15, -0.59) |
| Nepal | 114.2 (103.4, 125.4) | 142.2 (130.3, 156.6) | 0.77 (0.71, 0.84) | 0.2 (0.1, 0.4) | 0.4 (0.3, 0.6) | 2.13 (1.83, 2.44) | 0.9 (0.5, 1.6) | 1.6 (1.1, 2.4) | 2.33 (2.02, 2.63) |
| Netherlands | 498.0 (456.3, 543.4) | 694.7 (634.8, 757.4) | 1.43 (0.81, 2.06) | 1.3 (1.0, 1.8) | 1.5 (1.1, 1.9) | 0.54 (-0.13, 1.22) | 5.0 (3.6, 6.6) | 6.3 (4.7, 8.0) | 0.93 (0.28, 1.58) |
| New Zealand | 390.3 (355.9, 427.7) | 473.0 (431.9, 516.3) | 0.66 (0.52, 0.80) | 0.8 (0.7, 1.1) | 1.1 (0.9, 1.3) | 0.91 (0.77, 1.04) | 3.1 (2.5, 3.9) | 4.4 (3.6, 5.1) | 1.04 (0.89, 1.20) |
| Nicaragua | 18.5 (16.9, 20.2) | 23.3 (21.3, 25.4) | 0.76 (0.67, 0.85) | 0.1 (0.1, 0.1) | 0.1 (0.1, 0.2) | 1.95 (1.76, 2.14) | 0.3 (0.2, 0.4) | 0.5 (0.4, 0.7) | 1.91 (1.71, 2.10) |
| Niger | 37.5 (34.1, 41.2) | 47.5 (43.0, 52.1) | 0.81 (0.77, 0.86) | 0.2 (0.1, 0.4) | 0.2 (0.1, 0.3) | -0.47 (-0.52, -0.41) | 0.9 (0.5, 1.5) | 0.8 (0.5, 1.4) | -0.35 (-0.39, -0.30) |
| Nigeria | 47.6 (43.6, 52.0) | 68.9 (62.9, 75.6) | 1.28 (1.20, 1.37) | 0.2 (0.1, 0.3) | 0.2 (0.2, 0.3) | 0.75 (0.66, 0.83) | 0.8 (0.5, 1.3) | 1.0 (0.7, 1.4) | 0.79 (0.69, 0.88) |
| Niue | 28.8 (26.2, 31.6) | 31.8 (29.2, 34.6) | 0.31 (0.26, 0.36) | 0.1 (0.1, 0.1) | 0.1 (0.1, 0.1) | 0.36 (0.19, 0.53) | 0.3 (0.2, 0.4) | 0.3 (0.2, 0.5) | 0.49 (0.33, 0.64) |
| North Macedonia | 188.3 (171.8, 206.8) | 237.1 (216.0, 259.5) | 0.68 (0.60, 0.76) | 0.5 (0.4, 0.7) | 0.6 (0.4, 0.8) | 0.18 (0.06, 0.29) | 2.1 (1.5, 2.9) | 2.7 (1.9, 3.7) | 0.53 (0.42, 0.64) |
| Northern Mariana Islands | 36.8 (33.8, 40.2) | 37.7 (34.4, 41.0) | -0.05 (-0.13, 0.03) | 0.2 (0.1, 0.3) | 0.2 (0.1, 0.2) | -0.89 (-1.09, -0.70) | 0.7 (0.5, 1.0) | 0.7 (0.5, 0.9) | -0.78 (-0.96, -0.59) |
| Norway | 391.8 (357.4, 429.2) | 571.5 (524.2, 623.7) | 0.98 (0.88, 1.08) | 0.9 (0.7, 1.2) | 1.1 (0.9, 1.4) | 0.34 (0.08, 0.61) | 3.4 (2.5, 4.4) | 4.6 (3.7, 5.6) | 0.70 (0.46, 0.94) |
| Oman | 152.3 (139.1, 166.4) | 270.4 (249.8, 294.4) | 2.22 (2.01, 2.43) | 0.2 (0.1, 0.4) | 0.5 (0.3, 0.6) | 3.20 (2.82, 3.58) | 1.1 (0.6, 1.8) | 2.2 (1.4, 3.0) | 3.48 (3.10, 3.85) |
| Pakistan | 129.3 (117.7, 141.4) | 153.9 (139.9, 168.1) | 0.76 (0.65, 0.87) | 0.3 (0.2, 0.6) | 0.4 (0.2, 0.5) | 0.49 (0.41, 0.57) | 1.2 (0.6, 2.3) | 1.4 (0.9, 2.0) | 0.59 (0.51, 0.67) |
| Palau | 29.0 (26.5, 31.8) | 31.9 (29.3, 34.6) | 0.26 (0.21, 0.32) | 0.1 (0.1, 0.1) | 0.1 (0.1, 0.2) | 0.42 (0.18, 0.65) | 0.3 (0.2, 0.4) | 0.4 (0.2, 0.5) | 0.31 (0.08, 0.54) |
| Palestine | 154.5 (142.3, 168.2) | 220.6 (202.8, 239.2) | 1.45 (1.30, 1.60) | 0.3 (0.2, 0.6) | 0.3 (0.2, 0.4) | -0.19 (-0.37, -0.02) | 1.6 (1.0, 2.9) | 1.5 (1.1, 2.0) | -0.10 (-0.29, 0.10) |
| Panama | 31.5 (28.8, 34.6) | 40.4 (37.0, 44.4) | 0.79 (0.76, 0.83) | 0.1 (0.1, 0.1) | 0.1 (0.1, 0.2) | 0.87 (0.67, 1.06) | 0.4 (0.3, 0.5) | 0.5 (0.3, 0.7) | 0.79 (0.57, 1.01) |
| Papua New Guinea | 17.6 (16.0, 19.4) | 19.4 (17.8, 21.3) | 0.29 (0.23, 0.35) | 0.0 (0.0, 0.0) | 0.0 (0.0, 0.0) | -1.22 (-1.37, -1.07) | 0.1 (0.0, 0.2) | 0.1 (0.0, 0.1) | -1.22 (-1.36, -1.08) |
| Paraguay | 52.2 (47.6, 57.3) | 48.5 (44.3, 53.0) | 0.08 (-0.20, 0.35) | 0.1 (0.1, 0.1) | 0.1 (0.1, 0.1) | 1.55 (1.23, 1.87) | 0.3 (0.2, 0.4) | 0.4 (0.3, 0.5) | 1.61 (1.27, 1.94) |
| Peru | 41.3 (37.8, 45.0) | 48.4 (44.2, 52.8) | 0.53 (0.40, 0.66) | 0.4 (0.3, 0.5) | 0.5 (0.3, 0.7) | 0.24 (-0.16, 0.63) | 1.4 (1.0, 2.0) | 1.8 (1.2, 2.7) | 0.56 (0.19, 0.92) |
| Philippines | 15.6 (14.2, 17.0) | 14.8 (13.6, 16.2) | -0.16 (-0.21, -0.11) | 0.0 (0.0, 0.0) | 0.0 (0.0, 0.0) | -0.29 (-0.37, -0.20) | 0.1 (0.1, 0.2) | 0.1 (0.1, 0.1) | -0.15 (-0.23, -0.06) |
| Poland | 80.5 (73.7, 87.7) | 86.6 (79.4, 94.0) | 0.39 (0.31, 0.46) | 0.2 (0.1, 0.2) | 0.3 (0.2, 0.4) | 2.54 (2.32, 2.77) | 0.6 (0.4, 0.8) | 1.0 (0.8, 1.4) | 1.92 (1.74, 2.11) |
| Portugal | 160.2 (145.9, 176.2) | 238.2 (217.1, 261.5) | 1.34 (1.14, 1.54) | 1.5 (1.0, 2.0) | 0.9 (0.7, 1.1) | -1.95 (-2.31, -1.59) | 5.2 (3.7, 7.0) | 3.3 (2.5, 4.3) | -1.76 (-2.04, -1.47) |
| Puerto Rico | 127.9 (116.8, 139.8) | 149.8 (137.5, 162.8) | 0.61 (0.55, 0.66) | 1.1 (0.8, 1.5) | 0.8 (0.6, 1.1) | -1.27 (-1.61, -0.93) | 4.0 (3.0, 5.3) | 3.1 (2.2, 4.1) | -1.16 (-1.47, -0.84) |
| Qatar | 219.6 (202.2, 238.0) | 342.6 (318.0, 369.6) | 1.71 (1.56, 1.87) | 1.0 (0.7, 1.5) | 1.4 (0.9, 2.1) | 1.07 (0.37, 1.77) | 5.5 (3.6, 7.9) | 7.6 (5.0, 10.7) | 1.07 (0.28, 1.85) |
| Republic of Korea | 88.4 (80.4, 97.6) | 153.8 (140.8, 167.7) | 2.95 (2.17, 3.74) | 0.8 (0.5, 1.0) | 0.4 (0.3, 0.6) | -1.50 (-2.00, -1.00) | 3.3 (2.4, 4.5) | 2.0 (1.4, 2.9) | -1.16 (-1.62, -0.69) |
| Republic of Moldova | 93.4 (85.2, 102.6) | 106.8 (97.4, 116.9) | 0.71 (0.44, 0.98) | 1.3 (0.9, 1.9) | 1.4 (1.0, 2.0) | 0.66 (0.32, 1.00) | 4.5 (3.1, 6.5) | 4.8 (3.4, 6.6) | 0.58 (0.28, 0.88) |
| Romania | 44.8 (40.7, 48.8) | 52.5 (47.7, 57.5) | 1.03 (0.23, 1.83) | 0.2 (0.1, 0.3) | 0.3 (0.2, 0.5) | 1.80 (0.91, 2.70) | 0.6 (0.5, 0.9) | 1.2 (0.9, 1.7) | 1.97 (1.09, 2.85) |
| Russian Federation | 105.6 (96.8, 114.9) | 115.2 (105.3, 125.4) | 0.35 (0.13, 0.58) | 0.2 (0.2, 0.3) | 0.9 (0.6, 1.2) | 4.85 (4.09, 5.62) | 0.8 (0.6, 1.0) | 2.6 (1.9, 3.5) | 4.29 (3.71, 4.87) |
| Rwanda | 31.1 (28.1, 34.1) | 36.4 (33.1, 40.0) | 0.37 (0.23, 0.51) | 0.2 (0.1, 0.3) | 0.2 (0.1, 0.3) | -1.35 (-1.77, -0.92) | 0.9 (0.6, 1.2) | 0.8 (0.5, 1.2) | -1.04 (-1.43, -0.64) |
| Saint Kitts and Nevis | 103.3 (94.4, 113.3) | 122.2 (112.1, 133.7) | 0.68 (0.56, 0.80) | 0.8 (0.6, 1.1) | 0.7 (0.5, 1.0) | -0.23 (-0.89, 0.43) | 2.9 (2.1, 3.9) | 2.7 (1.9, 3.7) | 0.02 (-0.56, 0.61) |
| Saint Lucia | 85.0 (77.4, 93.2) | 108.2 (98.9, 117.5) | 0.69 (0.60, 0.78) | 0.6 (0.4, 0.8) | 0.6 (0.4, 0.8) | -0.54 (-1.00, -0.08) | 2.4 (1.7, 3.3) | 2.2 (1.5, 3.1) | -0.83 (-1.36, -0.31) |
| Saint Vincent and the Grenadines | 88.2 (80.4, 97.0) | 102.2 (93.4, 112.0) | 0.63 (0.57, 0.70) | 0.3 (0.2, 0.4) | 0.5 (0.3, 0.6) | 1.31 (0.77, 1.85) | 1.2 (0.9, 1.6) | 1.7 (1.2, 2.3) | 1.11 (0.55, 1.67) |
| Samoa | 26.6 (24.3, 29.1) | 28.3 (25.9, 30.9) | 0.05 (-0.03, 0.14) | 0.1 (0.0, 0.1) | 0.1 (0.0, 0.1) | -0.50 (-0.69, -0.31) | 0.3 (0.2, 0.4) | 0.3 (0.2, 0.4) | -0.50 (-0.69, -0.31) |
| San Marino | 552.3 (502.0, 604.9) | 728.2 (665.4, 794.3) | 1.15 (1.01, 1.29) | 4.1 (2.8, 5.5) | 3.0 (1.8, 4.6) | -0.02 (-0.44, 0.40) | 18.3 (12.4, 25.0) | 12.5 (7.5, 19.2) | -0.24 (-0.65, 0.18) |
| Sao Tome and Principe | 52.8 (48.2, 58.0) | 69.6 (63.1, 76.7) | 0.94 (0.88, 1.00) | 0.3 (0.2, 0.4) | 0.4 (0.2, 0.6) | 0.79 (0.65, 0.93) | 1.2 (0.8, 1.8) | 1.5 (0.8, 2.4) | 0.85 (0.74, 0.95) |
| Saudi Arabia | 129.0 (118.6, 141.0) | 218.7 (202.7, 237.1) | 2.01 (1.86, 2.17) | 0.5 (0.3, 0.9) | 0.8 (0.6, 1.2) | 2.04 (1.91, 2.18) | 2.3 (1.2, 4.7) | 4.1 (2.7, 5.7) | 2.10 (1.96, 2.25) |
| Senegal | 50.1 (45.7, 54.9) | 67.0 (60.9, 73.2) | 0.75 (0.61, 0.88) | 0.2 (0.2, 0.3) | 0.3 (0.2, 0.4) | 0.05 (-0.07, 0.17) | 0.9 (0.7, 1.2) | 1.1 (0.7, 1.5) | 0.14 (0.02, 0.26) |
| Serbia | 234.7 (214.1, 256.3) | 283.0 (258.9, 307.9) | 0.78 (0.63, 0.94) | 0.5 (0.3, 0.6) | 0.5 (0.4, 0.7) | 0.08 (-0.02, 0.17) | 1.8 (1.3, 2.5) | 1.9 (1.4, 2.7) | 0.10 (0.00, 0.19) |
| Seychelles | 23.7 (21.7, 25.8) | 27.1 (24.8, 29.8) | 0.47 (0.42, 0.52) | 0.1 (0.1, 0.1) | 0.1 (0.1, 0.1) | -0.30 (-0.40, -0.19) | 0.3 (0.3, 0.4) | 0.3 (0.2, 0.4) | -0.10 (-0.20, 0.00) |
| Sierra Leone | 34.3 (31.1, 37.8) | 46.3 (42.2, 51.1) | 1.01 (0.91, 1.12) | 0.2 (0.1, 0.4) | 0.2 (0.1, 0.3) | 0.04 (-0.07, 0.16) | 0.8 (0.5, 1.5) | 0.8 (0.6, 1.2) | -0.01 (-0.13, 0.11) |
| Singapore | 22.1 (20.2, 24.2) | 29.0 (26.5, 31.7) | 0.74 (0.61, 0.87) | 0.0 (0.0, 0.1) | 0.0 (0.0, 0.0) | -1.79 (-2.14, -1.45) | 0.2 (0.1, 0.2) | 0.1 (0.1, 0.2) | -1.26 (-1.62, -0.90) |
| Slovakia | 169.3 (154.8, 184.7) | 200.1 (182.6, 218.4) | 0.41 (0.32, 0.49) | 0.9 (0.6, 1.2) | 0.8 (0.6, 1.1) | -0.23 (-0.38, -0.08) | 2.9 (2.2, 3.9) | 2.8 (2.0, 3.9) | -0.19 (-0.32, -0.06) |
| Slovenia | 248.3 (227.0, 272.2) | 306.1 (278.4, 335.2) | 0.65 (0.49, 0.81) | 1.3 (0.9, 1.8) | 0.8 (0.6, 1.1) | -1.76 (-2.29, -1.22) | 4.4 (3.2, 6.1) | 3.5 (2.5, 4.7) | -1.04 (-1.53, -0.56) |
| Solomon Islands | 17.3 (15.7, 19.0) | 19.5 (17.7, 21.4) | 0.36 (0.31, 0.41) | 0.1 (0.0, 0.1) | 0.1 (0.0, 0.1) | 0.49 (0.34, 0.65) | 0.2 (0.1, 0.3) | 0.2 (0.1, 0.3) | 0.44 (0.29, 0.59) |
| Somalia | 36.7 (33.4, 40.1) | 39.7 (36.3, 43.4) | 0.07 (-0.02, 0.15) | 0.2 (0.1, 0.3) | 0.2 (0.1, 0.3) | -0.61 (-0.78, -0.45) | 0.9 (0.6, 1.4) | 0.9 (0.5, 1.3) | -0.44 (-0.60, -0.29) |
| South Africa | 55.5 (50.7, 60.7) | 75.6 (69.3, 82.5) | 0.90 (0.86, 0.94) | 0.2 (0.1, 0.3) | 0.3 (0.3, 0.4) | 1.54 (1.01, 2.09) | 0.7 (0.4, 1.0) | 1.2 (1.0, 1.5) | 1.75 (1.25, 2.26) |
| South Sudan | 39.3 (36.0, 43.2) | 45.3 (41.3, 49.7) | 0.23 (0.17, 0.30) | 0.2 (0.1, 0.3) | 0.2 (0.2, 0.3) | 0.72 (0.49, 0.95) | 0.7 (0.5, 1.0) | 0.9 (0.6, 1.4) | 0.74 (0.53, 0.95) |
| Spain | 249.1 (227.7, 271.5) | 334.0 (304.9, 365.8) | 0.77 (0.28, 1.28) | 1.8 (1.4, 2.5) | 1.0 (0.7, 1.3) | -2.29 (-2.85, -1.72) | 7.1 (5.1, 9.6) | 4.1 (3.0, 5.3) | -2.11 (-2.66, -1.55) |
| Sri Lanka | 29.8 (27.3, 32.6) | 35.2 (32.3, 38.4) | 0.93 (0.66, 1.20) | 0.0 (0.0, 0.1) | 0.0 (0.0, 0.0) | -1.57 (-1.79, -1.35) | 0.2 (0.1, 0.3) | 0.1 (0.1, 0.2) | -1.18 (-1.37, -0.99) |
| Sudan | 88.2 (80.1, 96.7) | 148.0 (135.0, 161.9) | 1.94 (1.77, 2.10) | 0.2 (0.1, 0.3) | 0.3 (0.2, 0.5) | 2.04 (1.75, 2.33) | 0.8 (0.4, 1.7) | 1.3 (0.8, 2.5) | 2.09 (1.79, 2.38) |
| Suriname | 89.4 (81.1, 97.8) | 107.5 (98.2, 117.3) | 0.66 (0.62, 0.69) | 0.6 (0.4, 0.9) | 0.6 (0.4, 0.9) | 0.21 (-0.04, 0.46) | 2.3 (1.6, 3.1) | 2.4 (1.5, 3.4) | 0.23 (0.02, 0.45) |
| Sweden | 441.8 (403.9, 480.5) | 550.5 (504.9, 599.5) | 0.85 (0.70, 1.00) | 1.1 (0.8, 1.4) | 1.0 (0.7, 1.4) | 0.53 (0.13, 0.93) | 4.1 (3.0, 5.2) | 4.1 (2.8, 5.7) | 0.81 (0.42, 1.20) |
| Switzerland | 276.4 (252.8, 301.5) | 355.5 (324.3, 386.8) | 0.87 (0.85, 0.89) | 1.2 (0.9, 1.6) | 0.8 (0.6, 1.1) | -1.11 (-1.37, -0.84) | 4.2 (3.2, 5.5) | 3.5 (2.6, 4.5) | -0.58 (-0.88, -0.29) |
| Syrian Arab Republic | 149.6 (136.8, 163.1) | 240.2 (221.6, 261.4) | 1.87 (1.59, 2.15) | 0.3 (0.2, 0.4) | 0.4 (0.3, 0.6) | 1.35 (1.23, 1.47) | 1.3 (0.9, 2.1) | 2.0 (1.3, 2.9) | 1.58 (1.43, 1.73) |
| Taiwan (Province of China) | 23.3 (21.2, 25.4) | 29.1 (26.6, 31.7) | 2.34 (1.29, 3.41) | 0.1 (0.0, 0.1) | 0.1 (0.0, 0.1) | 2.39 (1.27, 3.52) | 0.2 (0.2, 0.3) | 0.2 (0.2, 0.3) | 2.66 (1.51, 3.82) |
| Tajikistan | 148.1 (134.1, 162.3) | 163.8 (149.5, 179.6) | 0.48 (0.36, 0.61) | 0.4 (0.3, 0.6) | 0.4 (0.3, 0.6) | -0.78 (-0.93, -0.64) | 1.7 (1.1, 2.3) | 1.4 (1.0, 2.0) | -0.66 (-0.86, -0.47) |
| Thailand | 17.0 (15.5, 18.6) | 21.9 (20.0, 24.0) | 0.91 (0.88, 0.94) | 0.1 (0.0, 0.1) | 0.1 (0.1, 0.1) | 0.71 (0.48, 0.95) | 0.3 (0.2, 0.4) | 0.3 (0.2, 0.5) | 0.65 (0.41, 0.89) |
| Timor-Leste | 16.5 (15.0, 18.2) | 19.2 (17.5, 21.0) | 0.60 (0.55, 0.65) | 0.0 (0.0, 0.0) | 0.0 (0.0, 0.0) | -0.01 (-0.27, 0.25) | 0.1 (0.1, 0.2) | 0.1 (0.1, 0.2) | 0.11 (-0.12, 0.34) |
| Togo | 40.2 (36.6, 44.1) | 50.7 (46.5, 55.9) | 0.72 (0.68, 0.77) | 0.2 (0.1, 0.2) | 0.2 (0.1, 0.3) | 0.58 (0.51, 0.65) | 0.7 (0.5, 1.0) | 0.9 (0.6, 1.3) | 0.68 (0.61, 0.74) |
| Tokelau | 22.1 (20.2, 24.3) | 29.3 (26.8, 32.0) | 1.06 (0.98, 1.15) | 0.1 (0.0, 0.1) | 0.1 (0.1, 0.1) | 0.62 (0.52, 0.73) | 0.2 (0.1, 0.3) | 0.2 (0.2, 0.3) | 0.56 (0.46, 0.66) |
| Tonga | 28.3 (25.9, 30.8) | 32.3 (29.7, 35.1) | 0.30 (0.20, 0.40) | 0.2 (0.1, 0.3) | 0.2 (0.1, 0.3) | 0.00 (-0.40, 0.41) | 0.7 (0.5, 1.0) | 0.8 (0.5, 1.1) | 0.05 (-0.36, 0.47) |
| Trinidad and Tobago | 98.8 (90.0, 108.6) | 121.6 (111.3, 132.9) | 1.00 (0.87, 1.14) | 0.5 (0.4, 0.7) | 0.5 (0.3, 0.7) | 0.20 (-0.12, 0.52) | 2.0 (1.4, 2.6) | 1.7 (1.2, 2.5) | -0.11 (-0.45, 0.23) |
| Tunisia | 159.5 (145.7, 174.6) | 259.2 (239.1, 281.4) | 1.81 (1.67, 1.96) | 0.2 (0.1, 0.3) | 0.4 (0.2, 0.5) | 2.14 (1.93, 2.36) | 1.0 (0.6, 1.7) | 1.7 (1.1, 2.7) | 2.18 (1.95, 2.41) |
| Turkmenistan | 153.4 (139.9, 168.0) | 177.4 (162.5, 192.2) | 0.59 (0.53, 0.65) | 0.8 (0.5, 1.0) | 2.0 (1.4, 2.9) | 3.68 (3.40, 3.97) | 2.8 (2.0, 3.9) | 6.4 (4.4, 9.1) | 3.03 (2.77, 3.30) |
| Tuvalu | 20.0 (18.1, 21.9) | 23.0 (21.0, 25.0) | 0.44 (0.40, 0.49) | 0.1 (0.0, 0.1) | 0.1 (0.0, 0.1) | -0.10 (-0.19, -0.01) | 0.2 (0.1, 0.3) | 0.2 (0.2, 0.3) | -0.03 (-0.11, 0.05) |
| Uganda | 28.0 (25.5, 30.6) | 37.6 (34.2, 41.3) | 0.99 (0.93, 1.05) | 0.1 (0.1, 0.2) | 0.2 (0.1, 0.3) | 0.45 (0.22, 0.67) | 0.5 (0.4, 0.8) | 0.7 (0.5, 1.1) | 0.53 (0.33, 0.74) |
| Ukraine | 98.0 (89.3, 107.1) | 91.2 (83.1, 99.5) | -0.12 (-0.25, 0.02) | 0.3 (0.2, 0.4) | 0.6 (0.4, 1.0) | 2.50 (1.59, 3.41) | 0.9 (0.7, 1.2) | 1.7 (1.1, 2.6) | 1.96 (1.20, 2.73) |
| United Arab Emirate | 199.5 (184.2, 216.5) | 293.3 (272.0, 316.9) | 1.56 (1.36, 1.76) | 0.6 (0.4, 0.8) | 1.1 (0.7, 1.5) | 3.40 (2.83, 3.98) | 2.7 (1.8, 3.8) | 5.4 (3.7, 8.0) | 4.22 (3.54, 4.90) |
| United Kingdom | 259.5 (238.4, 283.6) | 390.7 (359.3, 425.6) | 0.75 (0.54, 0.96) | 1.1 (0.8, 1.4) | 2.7 (2.1, 3.3) | 2.58 (1.83, 3.33) | 3.6 (2.8, 4.5) | 9.0 (7.3, 10.8) | 2.63 (1.98, 3.29) |
| United Republic of Tanzania | 38.5 (34.9, 42.3) | 50.2 (45.6, 55.3) | 0.74 (0.69, 0.79) | 0.2 (0.1, 0.2) | 0.2 (0.1, 0.3) | 0.45 (0.34, 0.56) | 0.7 (0.5, 0.9) | 0.8 (0.5, 1.2) | 0.53 (0.41, 0.64) |
| United States of America | 376.4 (343.7, 410.6) | 479.0 (437.1, 520.6) | 0.98 (0.82, 1.14) | 1.4 (1.0, 1.9) | 1.9 (1.5, 2.5) | 1.31 (1.16, 1.47) | 5.0 (3.7, 6.5) | 7.0 (5.4, 8.8) | 1.39 (1.24, 1.54) |
| United States Virgin Islands | 109.2 (99.5, 120.4) | 136.2 (124.8, 147.9) | 0.79 (0.75, 0.84) | 0.9 (0.7, 1.3) | 0.9 (0.6, 1.2) | 0.09 (-0.06, 0.23) | 3.5 (2.4, 4.7) | 3.0 (2.1, 4.0) | -0.40 (-0.54, -0.25) |
| Uruguay | 115.5 (105.6, 126.5) | 135.5 (123.9, 147.8) | 0.49 (0.45, 0.54) | 0.4 (0.3, 0.5) | 0.4 (0.3, 0.5) | -0.39 (-0.53, -0.25) | 1.5 (1.0, 2.0) | 1.4 (1.0, 1.8) | -0.34 (-0.48, -0.21) |
| Uzbekistan | 165.1 (151.0, 181.1) | 177.6 (162.5, 194.7) | 0.39 (0.27, 0.52) | 0.7 (0.5, 0.9) | 0.9 (0.6, 1.2) | 0.69 (0.44, 0.95) | 2.3 (1.6, 3.3) | 2.9 (2.0, 4.0) | 0.63 (0.38, 0.88) |
| Vanuatu | 21.6 (19.8, 23.8) | 25.6 (23.4, 27.7) | 0.52 (0.48, 0.56) | 0.1 (0.0, 0.1) | 0.1 (0.0, 0.1) | 0.10 (0.02, 0.17) | 0.2 (0.1, 0.4) | 0.2 (0.1, 0.4) | 0.13 (0.06, 0.21) |
| Venezuela (Bolivarian Republic of) | 35.2 (32.1, 38.4) | 34.0 (31.2, 37.4) | 0.31 (0.06, 0.55) | 0.2 (0.1, 0.3) | 0.2 (0.1, 0.2) | -0.27 (-0.85, 0.31) | 0.7 (0.5, 0.9) | 0.6 (0.4, 0.8) | -0.35 (-0.94, 0.25) |
| Viet Nam | 18.7 (17.1, 20.5) | 21.1 (19.2, 23.0) | 0.55 (0.50, 0.59) | 0.1 (0.0, 0.1) | 0.1 (0.0, 0.1) | 0.24 (0.12, 0.37) | 0.2 (0.2, 0.4) | 0.3 (0.2, 0.4) | 0.28 (0.18, 0.39) |
| Yemen | 100.0 (91.0, 109.2) | 155.0 (141.9, 169.8) | 1.74 (1.55, 1.92) | 0.2 (0.1, 0.3) | 0.1 (0.1, 0.2) | -0.23 (-0.39, -0.06) | 0.7 (0.3, 1.6) | 0.7 (0.4, 1.2) | -0.19 (-0.35, -0.04) |
| Zambia | 35.2 (32.1, 38.6) | 46.7 (42.8, 51.4) | 0.69 (0.54, 0.85) | 0.2 (0.2, 0.3) | 0.2 (0.1, 0.3) | -1.07 (-1.40, -0.74) | 0.9 (0.6, 1.4) | 0.9 (0.6, 1.4) | -0.88 (-1.19, -0.57) |
| Zimbabwe | 37.4 (34.0, 41.2) | 48.2 (43.7, 52.6) | 0.21 (-0.05, 0.48) | 0.2 (0.1, 0.2) | 0.3 (0.2, 0.5) | 1.01 (0.32, 1.70) | 0.8 (0.5, 1.0) | 1.3 (0.7, 1.9) | 0.88 (0.25, 1.52) |

Numbers in parenthesis represent 95% uncertainty intervals (UIs). Numbers in parenthesis represent 95% confidence intervals (CIs). DALYs=disability-adjusted life years. IBD=inflammatory bowel disease. EAPC=estimated annual percentage change. MASLD=metabolic dysfunction-associated steatotic liver disease. SDI=socio-demographic index.

**Table S5.** Prevalent cases, DALYs, and deaths due to MASLD–IBD comorbidity in 1990 and 2021 and relative changes between 1990 and 2021, stratified by SDI, region, and country

| **Location** | **Prevalent cases** | | | **DALYs** | | | **Deaths** | | |
| --- | --- | --- | --- | --- | --- | --- | --- | --- | --- |
|  | **1990 No.(95% UI)** | **2021 No.(95% UI)** | **Relative change %(95% CI)** | **1990 No.(95% UI)** | **2021 No.(95% UI)** | **Relative change %(95% CI)** | **1990 No.(95% UI)** | **2021 No.(95% UI)** | **Relative change %(95% CI)** |
| **Global** | 58513.0 (53546.6, 64076.7) | 156776.4 (143182.6, 170695.7) | 155.18 (123.22,188.85) | 17512.0 (13398.7, 22874.5) | 45347.1 (35903.7, 56971.0) | 149.77 (71.38,255.43) | 617.2 (473.4, 791.0) | 1710.5 (1339.0, 2150.4) | 166.59 (84.91,275.31) |
| **SDI** |  |  |  |  |  |  |  |  |  |
| High SDI | 32656.7 (29915.6, 35586.7) | 79132.5 (72581.7, 85722.1) | 133.94 (106.05,163.28) | 15299.7 (11856.9, 19778.0) | 32680.6 (25705.1, 41053.6) | 112.03 (46.37,199.20) | 587.3 (445.9, 758.4) | 1406.0 (1084.3, 1755.9) | 135.25 (61.20,235.52) |
| High-middle SDI | 11779.5 (10785.0, 12885.5) | 27621.1 (25328.8, 30011.2) | 124.02 (96.90,152.60) | 3465.8 (2643.7, 4458.9) | 7550.7 (5866.7, 9703.7) | 116.73 (47.50,209.33) | 127.3 (96.8, 162.5) | 285.9 (221.2, 362.0) | 121.43 (52.36,212.73) |
| Middle SDI | 5538.2 (5074.3, 6093.9) | 25702.1 (23491.3, 28026.9) | 333.80 (279.13,391.47) | 1411.1 (1077.5, 1819.2) | 7139.3 (5617.4, 9011.8) | 369.24 (225.62,558.95) | 46.2 (35.5, 58.7) | 263.7 (207.5, 331.6) | 428.41 (270.23,636.15) |
| Low-middle SDI | 5674.5 (5197.6, 6251.3) | 20542.8 (18763.5, 22441.3) | 238.74 (195.68,284.19) | 1424.3 (1038.5, 2007.4) | 5513.1 (4196.6, 7173.9) | 261.59 (129.98,456.20) | 48.3 (34.8, 67.4) | 195.0 (144.7, 253.0) | 277.69 (140.52,480.21) |
| Low SDI | 1306.2 (1191.0, 1441.9) | 4086.1 (3738.9, 4521.3) | 186.43 (147.68,227.44) | 393.3 (287.8, 535.9) | 958.0 (743.6, 1233.1) | 125.77 (47.56,237.50) | 13.1 (9.5, 18.1) | 32.0 (24.9, 41.4) | 127.76 (46.79,244.54) |
| **Regions** |  |  |  |  |  |  |  |  |  |
| High-income Asia Pacific | 1336.4 (1223.2, 1457.2) | 3516.5 (3223.5, 3814.1) | 153.88 (123.02,186.36) | 684.4 (554.9, 853.6) | 1131.6 (874.1, 1389.1) | 56.44 (12.24,111.85) | 27.3 (21.5, 33.6) | 64.8 (48.6, 81.2) | 121.90 (56.08,206.11) |
| Central Asia | 738.1 (676.1, 809.0) | 1790.2 (1634.9, 1963.2) | 129.82 (100.38,160.93) | 271.2 (204.4, 356.4) | 978.1 (708.4, 1344.5) | 250.57 (125.16,423.16) | 9.4 (7.0, 12.4) | 32.4 (23.5, 43.9) | 234.67 (114.90,401.72) |
| East Asia | 1927.3 (1761.6, 2125.6) | 8979.3 (8174.9, 9796.0) | 339.03 (283.45,397.74) | 357.3 (274.9, 448.2) | 1296.5 (1003.0, 1644.7) | 253.70 (147.59,391.27) | 11.8 (9.1, 14.9) | 52.4 (40.4, 65.5) | 328.18 (199.47,495.09) |
| South Asia | 6966.8 (6357.2, 7695.1) | 27658.3 (25230.6, 30254.5) | 270.11 (221.43,321.56) | 1470.7 (1064.2, 2065.2) | 5986.3 (4399.7, 7997.4) | 277.64 (135.48,492.22) | 45.1 (32.0, 63.9) | 206.7 (150.7, 275.9) | 323.82 (162.89,568.55) |
| Southeast Asia | 566.2 (518.0, 622.4) | 1785.2 (1626.1, 1953.3) | 205.48 (166.28,246.83) | 138.2 (100.8, 194.9) | 485.9 (364.6, 638.9) | 233.58 (112.15,412.90) | 4.6 (3.3, 6.6) | 17.8 (13.0, 23.3) | 265.52 (131.42,471.67) |
| Australasia | 891.4 (814.4, 971.2) | 2507.7 (2294.8, 2737.2) | 161.53 (128.93,195.85) | 267.2 (203.2, 348.8) | 1029.1 (823.6, 1258.2) | 267.53 (152.42,421.38) | 9.7 (7.4, 12.5) | 43.5 (34.7, 53.4) | 322.16 (193.50,492.65) |
| Caribbean | 257.0 (235.0, 281.1) | 617.8 (566.7, 670.6) | 134.89 (106.08,165.25) | 121.8 (87.4, 162.8) | 326.9 (228.6, 452.3) | 165.53 (63.70,312.01) | 4.4 (3.2, 5.8) | 12.2 (8.6, 16.7) | 170.46 (71.37,311.99) |
| Central Europe | 2992.4 (2727.0, 3252.0) | 4664.8 (4261.0, 5064.6) | 54.91 (36.05,74.77) | 1057.5 (779.4, 1420.5) | 2056.1 (1495.0, 2800.8) | 94.70 (22.18,198.21) | 37.8 (28.2, 50.5) | 79.3 (58.1, 106.9) | 107.71 (32.18,214.19) |
| Eastern Europe | 3183.0 (2910.7, 3467.8) | 4319.9 (3946.3, 4701.8) | 33.55 (16.96,51.00) | 800.3 (583.2, 1087.1) | 3364.3 (2403.2, 4640.5) | 323.17 (163.67,555.42) | 28.0 (20.6, 37.6) | 104.8 (76.7, 142.0) | 273.90 (135.80,471.35) |
| Western Europe | 16675.0 (15271.6, 18125.0) | 35867.3 (32988.1, 38737.8) | 109.66 (85.16,135.42) | 13163.1 (9745.3, 17244.1) | 18930.2 (14606.8, 23918.4) | 49.74 (0.31,118.07) | 525.9 (382.9, 679.7) | 849.5 (641.2, 1059.5) | 66.39 (11.64,142.03) |
| Andean Latin America | 86.7 (79.3, 95.0) | 334.5 (305.2, 365.9) | 266.70 (219.88,316.05) | 69.5 (48.1, 94.3) | 296.1 (206.4, 413.1) | 320.53 (161.97,558.07) | 2.4 (1.7, 3.2) | 11.8 (8.1, 16.3) | 397.57 (207.84,682.53) |
| Central Latin America | 179.3 (164.8, 197.0) | 661.8 (605.6, 722.4) | 252.83 (208.90,299.12) | 113.8 (82.0, 152.6) | 536.2 (400.6, 697.7) | 333.38 (180.21,553.84) | 3.7 (2.7, 4.9) | 18.7 (14.0, 24.1) | 367.33 (208.12,593.86) |
| Southern Latin America | 453.2 (414.5, 498.6) | 1247.5 (1138.5, 1362.1) | 162.96 (130.41,197.41) | 276.0 (193.6, 382.0) | 569.1 (413.6, 784.1) | 107.48 (26.21,231.02) | 9.8 (6.8, 13.4) | 22.8 (16.5, 30.6) | 134.43 (44.75,269.99) |
| Tropical Latin America | 480.6 (440.6, 532.1) | 2536.1 (2316.7, 2771.7) | 402.86 (338.50,470.85) | 99.5 (73.8, 132.5) | 599.2 (441.5, 788.9) | 468.80 (268.18,752.45) | 3.0 (2.2, 4.0) | 21.1 (15.8, 27.2) | 575.02 (343.03,903.18) |
| North Africa and Middle East | 2791.5 (2557.2, 3065.5) | 14474.1 (13340.3, 15772.3) | 379.75 (322.49,440.78) | 478.5 (335.8, 658.1) | 2434.0 (1808.8, 3295.6) | 365.67 (195.64,618.63) | 19.5 (13.2, 29.1) | 96.9 (70.6, 131.7) | 357.46 (171.95,676.66) |
| High-income North America | 14996.5 (13716.5, 16392.4) | 33633.2 (30666.0, 36672.6) | 118.31 (90.59,147.59) | 5754.3 (4358.5, 7571.2) | 16588.5 (12925.0, 21074.2) | 178.78 (87.98,303.61) | 215.3 (164.3, 276.7) | 673.9 (520.5, 849.8) | 200.21 (105.85,325.48) |
| Oceania | 6.6 (6.0, 7.3) | 19.1 (17.4, 21.1) | 176.72 (138.75,216.92) | 1.0 (0.7, 1.6) | 2.4 (1.7, 3.4) | 125.44 (25.00,322.30) | 0.0 (0.0, 0.0) | 0.1 (0.1, 0.1) | 132.60 (25.60,354.20) |
| Central Sub-Saharan Africa | 77.1 (69.7, 85.8) | 229.2 (207.7, 253.1) | 169.78 (131.18,210.73) | 26.8 (17.4, 41.0) | 64.4 (42.2, 97.0) | 116.69 (12.39,297.45) | 0.8 (0.5, 1.3) | 1.9 (1.3, 2.9) | 113.31 (5.95,302.54) |
| Eastern Sub-Saharan Africa | 225.9 (206.3, 249.4) | 778.0 (710.0, 864.9) | 204.43 (162.73,248.58) | 78.6 (59.5, 103.0) | 236.7 (179.1, 315.8) | 169.94 (75.70,299.05) | 2.7 (2.0, 3.5) | 8.0 (6.0, 10.5) | 170.49 (76.51,302.22) |
| Southern Sub-Saharan Africa | 130.3 (119.4, 143.3) | 455.7 (416.3, 499.3) | 221.60 (180.05,265.42) | 39.2 (29.1, 52.9) | 182.1 (144.2, 230.9) | 315.58 (179.89,505.69) | 1.2 (0.9, 1.8) | 6.1 (4.8, 7.6) | 343.40 (194.58,584.85) |
| Western Sub-Saharan Africa | 337.9 (309.8, 372.5) | 1171.3 (1071.1, 1294.4) | 211.12 (169.20,254.94) | 129.7 (89.0, 188.8) | 363.6 (269.0, 483.9) | 156.50 (54.85,321.24) | 4.6 (3.1, 7.0) | 12.4 (9.4, 16.4) | 147.81 (47.47,325.54) |
| **Country** |  |  |  |  |  |  |  |  |  |
| Afghanistan | 49.0 (44.6, 54.1) | 159.9 (145.1, 177.2) | 196.19 (155.41,239.48) | 14.3 (8.9, 21.9) | 20.9 (12.8, 31.5) | 32.55 (-33.82,149.14) | 0.5 (0.3, 0.9) | 0.7 (0.4, 1.0) | 20.06 (-41.29,153.17) |
| Albania | 56.0 (50.9, 61.9) | 108.6 (99.5, 117.9) | 87.38 (64.27,112.13) | 12.4 (9.0, 17.0) | 26.4 (18.0, 38.5) | 108.30 (25.57,229.31) | 0.5 (0.4, 0.7) | 1.2 (0.8, 1.9) | 134.76 (33.94,285.74) |
| Algeria | 192.0 (175.1, 211.2) | 1107.3 (1015.8, 1207.5) | 448.57 (382.33,519.30) | 13.3 (8.9, 19.9) | 94.6 (62.9, 139.8) | 555.28 (264.93,1043.04) | 0.5 (0.3, 0.8) | 4.1 (2.7, 6.2) | 633.05 (286.18,1306.59) |
| American Samoa | 0.1 (0.1, 0.1) | 0.2 (0.2, 0.2) | 125.49 (97.72,155.24) | 0.0 (0.0, 0.0) | 0.1 (0.1, 0.1) | 193.27 (82.28,361.36) | 0.0 (0.0, 0.0) | 0.0 (0.0, 0.0) | 232.70 (106.65,422.52) |
| Andorra | 2.3 (2.1, 2.6) | 7.5 (6.9, 8.2) | 198.65 (161.08,238.55) | 1.8 (1.1, 2.6) | 4.8 (3.0, 6.8) | 193.37 (67.48,403.63) | 0.1 (0.0, 0.1) | 0.2 (0.1, 0.3) | 230.41 (88.37,467.89) |
| Angola | 12.9 (11.7, 14.4) | 48.3 (43.4, 53.6) | 239.85 (189.56,293.37) | 5.8 (3.3, 10.2) | 17.4 (10.4, 28.6) | 165.57 (6.03,537.04) | 0.2 (0.1, 0.3) | 0.5 (0.3, 0.9) | 164.57 (3.52,558.56) |
| Antigua and Barbuda | 0.6 (0.5, 0.6) | 1.7 (1.5, 1.8) | 173.25 (138.83,209.50) | 0.2 (0.2, 0.3) | 0.6 (0.4, 0.8) | 200.91 (88.05,362.13) | 0.0 (0.0, 0.0) | 0.0 (0.0, 0.0) | 179.30 (74.37,330.32) |
| Argentina | 294.0 (268.3, 323.8) | 749.0 (683.9, 822.9) | 143.32 (112.56,175.86) | 138.6 (96.6, 193.5) | 253.0 (179.4, 351.1) | 90.09 (13.52,207.82) | 5.1 (3.5, 7.1) | 10.1 (7.1, 13.7) | 104.42 (23.19,228.90) |
| Armenia | 52.6 (47.9, 58.2) | 102.9 (93.9, 112.4) | 92.22 (67.72,118.43) | 11.9 (9.1, 15.5) | 34.3 (25.6, 45.7) | 186.53 (91.22,315.75) | 0.4 (0.3, 0.6) | 1.6 (1.2, 2.1) | 262.27 (136.45,436.78) |
| Australia | 735.3 (670.3, 801.8) | 2112.5 (1929.4, 2306.5) | 166.72 (133.37,201.86) | 232.6 (175.4, 306.9) | 924.3 (734.8, 1142.0) | 279.25 (157.17,445.80) | 8.4 (6.3, 10.9) | 39.1 (30.9, 48.4) | 337.92 (199.93,523.53) |
| Austria | 280.6 (254.0, 305.5) | 625.8 (568.4, 681.0) | 120.31 (92.67,149.46) | 274.5 (194.2, 373.8) | 361.7 (264.6, 472.1) | 43.05 (-9.51,120.90) | 10.4 (7.3, 13.9) | 16.2 (11.9, 20.9) | 66.08 (6.18,153.46) |
| Azerbaijan | 89.8 (81.4, 98.6) | 302.6 (276.5, 333.2) | 219.84 (176.40,266.06) | 34.0 (24.0, 45.5) | 140.6 (93.6, 197.0) | 302.59 (136.15,540.52) | 1.3 (0.9, 1.7) | 5.3 (3.5, 7.7) | 311.63 (130.09,569.49) |
| Bahamas | 2.0 (1.8, 2.2) | 6.1 (5.5, 6.6) | 191.60 (152.52,232.90) | 1.3 (0.9, 1.8) | 3.5 (2.5, 5.0) | 161.72 (53.29,317.02) | 0.0 (0.0, 0.1) | 0.1 (0.1, 0.2) | 173.65 (64.84,328.57) |
| Bahrain | 6.4 (5.8, 7.1) | 48.6 (44.5, 53.3) | 600.70 (506.42,703.20) | 0.8 (0.6, 1.1) | 5.8 (4.0, 8.5) | 473.07 (243.86,792.03) | 0.0 (0.0, 0.0) | 0.2 (0.2, 0.3) | 465.60 (239.43,801.31) |
| Bangladesh | 601.0 (545.8, 664.9) | 2878.9 (2620.7, 3167.0) | 343.06 (283.58,406.02) | 119.6 (83.0, 168.7) | 453.9 (297.8, 656.6) | 246.91 (95.66,481.14) | 3.7 (2.6, 5.3) | 16.3 (11.1, 23.5) | 293.19 (121.90,561.08) |
| Barbados | 3.4 (3.1, 3.7) | 7.5 (6.9, 8.2) | 115.91 (88.43,144.45) | 1.5 (1.1, 2.0) | 2.7 (1.8, 3.8) | 88.36 (15.46,191.05) | 0.1 (0.0, 0.1) | 0.1 (0.1, 0.2) | 89.92 (19.27,190.07) |
| Belarus | 154.7 (140.1, 170.7) | 217.0 (197.3, 237.2) | 39.06 (21.09,58.29) | 27.2 (20.4, 37.2) | 102.1 (68.6, 149.4) | 268.31 (121.32,476.81) | 1.0 (0.7, 1.3) | 3.4 (2.3, 4.8) | 233.59 (107.79,408.65) |
| Belgium | 275.5 (250.0, 300.7) | 606.7 (553.4, 657.8) | 115.90 (89.67,143.82) | 230.2 (163.7, 308.4) | 425.5 (324.1, 546.1) | 94.24 (25.91,193.09) | 9.8 (7.1, 12.9) | 18.3 (13.8, 22.9) | 96.33 (30.67,188.71) |
| Belize | 0.7 (0.7, 0.8) | 4.1 (3.7, 4.5) | 411.49 (344.05,482.71) | 0.3 (0.2, 0.4) | 2.5 (1.8, 3.3) | 772.96 (460.51,1214.81) | 0.0 (0.0, 0.0) | 0.1 (0.1, 0.1) | 661.71 (392.24,1047.92) |
| Benin | 6.2 (5.6, 6.9) | 28.1 (25.4, 31.3) | 309.66 (250.34,372.72) | 3.2 (2.0, 5.0) | 10.7 (7.4, 15.6) | 203.60 (61.56,481.61) | 0.1 (0.1, 0.2) | 0.4 (0.3, 0.5) | 187.27 (51.30,465.43) |
| Bermuda | 1.0 (0.9, 1.1) | 2.1 (1.9, 2.3) | 98.96 (74.23,125.03) | 0.5 (0.4, 0.7) | 0.7 (0.5, 1.0) | 33.86 (-12.94,98.29) | 0.0 (0.0, 0.0) | 0.0 (0.0, 0.0) | 47.32 (-5.08,118.80) |
| Bhutan | 3.1 (2.8, 3.5) | 12.8 (11.7, 14.1) | 282.96 (230.70,338.97) | 0.7 (0.4, 1.2) | 3.6 (2.4, 5.3) | 368.65 (138.28,849.10) | 0.0 (0.0, 0.0) | 0.1 (0.1, 0.2) | 474.93 (202.41,1039.70) |
| Bolivia (Plurinational State of) | 10.0 (9.1, 11.0) | 42.0 (38.5, 46.1) | 299.59 (247.74,354.40) | 10.6 (5.1, 16.8) | 48.3 (27.5, 76.6) | 330.54 (91.00,870.01) | 0.4 (0.2, 0.6) | 1.9 (1.0, 3.0) | 386.97 (114.70,1006.76) |
| Bosnia and Herzegovina | 94.1 (85.5, 103.5) | 142.1 (129.6, 155.0) | 55.34 (35.04,76.79) | 22.2 (16.5, 29.6) | 40.8 (28.3, 56.4) | 95.10 (25.42,193.65) | 0.8 (0.6, 1.1) | 1.8 (1.2, 2.5) | 134.85 (49.27,257.17) |
| Botswana | 2.2 (2.0, 2.4) | 11.6 (10.5, 12.7) | 376.73 (309.87,448.00) | 0.6 (0.4, 1.1) | 3.1 (2.0, 5.0) | 350.55 (107.26,930.27) | 0.0 (0.0, 0.0) | 0.1 (0.1, 0.2) | 360.46 (122.77,916.47) |
| Brazil | 469.1 (429.9, 519.4) | 2514.4 (2296.5, 2747.5) | 411.28 (345.82,480.44) | 97.8 (72.5, 130.2) | 596.3 (440.9, 786.0) | 475.69 (272.55,762.87) | 2.9 (2.2, 3.9) | 21.0 (15.8, 27.1) | 584.50 (348.70,918.47) |
| Brunei Darussalam | 0.4 (0.4, 0.5) | 1.9 (1.7, 2.1) | 307.25 (250.66,367.93) | 0.1 (0.1, 0.1) | 0.3 (0.2, 0.4) | 260.46 (118.81,475.46) | 0.0 (0.0, 0.0) | 0.0 (0.0, 0.0) | 259.98 (115.79,486.59) |
| Bulgaria | 252.4 (229.0, 276.6) | 265.9 (242.9, 289.2) | 8.33 (-5.55,22.99) | 107.8 (79.4, 144.6) | 131.7 (90.1, 187.1) | 22.48 (-24.59,89.08) | 4.1 (2.9, 5.5) | 5.0 (3.4, 7.1) | 22.60 (-25.90,92.64) |
| Burkina Faso | 8.7 (7.8, 9.6) | 31.4 (28.3, 34.7) | 228.49 (179.68,280.70) | 5.9 (3.3, 11.0) | 16.2 (9.5, 27.6) | 159.28 (-1.74,587.41) | 0.2 (0.1, 0.4) | 0.6 (0.3, 1.0) | 152.38 (-4.56,598.89) |
| Burundi | 7.0 (6.3, 7.8) | 19.7 (17.9, 22.1) | 147.23 (111.07,185.85) | 2.6 (1.8, 3.8) | 4.3 (3.0, 6.4) | 49.34 (-16.51,157.46) | 0.1 (0.1, 0.1) | 0.1 (0.1, 0.2) | 42.04 (-22.60,150.58) |
| Cabo Verde | 0.8 (0.7, 0.9) | 4.0 (3.6, 4.4) | 351.03 (288.04,417.83) | 0.3 (0.2, 0.5) | 1.6 (1.1, 2.3) | 390.19 (174.10,756.77) | 0.0 (0.0, 0.0) | 0.1 (0.0, 0.1) | 390.59 (159.39,859.48) |
| Cambodia | 6.7 (6.1, 7.4) | 22.1 (20.0, 24.2) | 217.73 (175.66,262.54) | 2.9 (1.7, 4.8) | 8.1 (5.3, 12.1) | 155.11 (27.57,429.37) | 0.1 (0.1, 0.2) | 0.3 (0.2, 0.4) | 173.95 (29.16,514.06) |
| Cameroon | 17.2 (15.6, 19.0) | 74.7 (67.8, 82.5) | 284.62 (229.51,343.38) | 10.0 (6.9, 13.8) | 32.8 (20.4, 48.2) | 192.92 (49.16,399.17) | 0.3 (0.2, 0.5) | 1.1 (0.6, 1.5) | 179.68 (45.49,378.87) |
| Canada | 2486.1 (2252.5, 2723.2) | 4859.8 (4430.0, 5328.8) | 88.00 (63.28,114.11) | 1130.5 (845.8, 1506.3) | 3821.2 (2924.4, 4857.8) | 227.96 (119.28,379.41) | 43.7 (32.0, 57.9) | 166.4 (127.4, 213.2) | 267.85 (143.34,443.17) |
| Central African Republic | 3.0 (2.7, 3.3) | 7.7 (6.9, 8.5) | 138.23 (104.34,174.30) | 1.5 (1.0, 2.1) | 3.2 (2.0, 4.9) | 102.52 (2.10,255.38) | 0.0 (0.0, 0.1) | 0.1 (0.1, 0.1) | 95.24 (-4.89,250.86) |
| Chad | 7.2 (6.5, 7.9) | 21.4 (19.3, 23.9) | 176.59 (137.34,218.45) | 3.2 (1.9, 5.6) | 8.1 (5.1, 13.3) | 136.68 (-0.56,455.94) | 0.1 (0.1, 0.2) | 0.3 (0.2, 0.5) | 115.10 (-13.64,434.26) |
| Chile | 117.8 (107.3, 129.7) | 435.4 (395.3, 474.4) | 252.61 (205.01,303.21) | 119.7 (83.6, 167.4) | 316.9 (231.2, 424.9) | 159.04 (58.64,313.35) | 4.0 (2.7, 5.4) | 12.7 (9.3, 17.0) | 212.81 (94.92,390.40) |
| China | 1844.1 (1685.9, 2035.4) | 8767.0 (7981.7, 9567.2) | 347.76 (290.98,407.84) | 340.8 (260.4, 427.6) | 1247.6 (954.9, 1595.8) | 257.25 (148.93,398.35) | 11.2 (8.6, 14.1) | 50.4 (38.7, 63.5) | 332.46 (201.45,502.34) |
| Colombia | 82.6 (75.2, 91.2) | 330.8 (301.1, 361.9) | 278.35 (229.57,330.08) | 14.3 (10.7, 18.6) | 70.1 (49.1, 95.5) | 337.05 (189.87,539.32) | 0.5 (0.4, 0.7) | 3.0 (2.1, 4.0) | 428.25 (246.52,681.97) |
| Comoros | 0.9 (0.9, 1.0) | 3.5 (3.1, 3.8) | 248.31 (197.83,302.16) | 0.3 (0.2, 0.4) | 1.0 (0.7, 1.5) | 234.37 (84.59,486.15) | 0.0 (0.0, 0.0) | 0.0 (0.0, 0.1) | 257.07 (90.85,530.18) |
| Congo | 3.7 (3.4, 4.1) | 15.5 (14.0, 17.1) | 290.38 (235.13,349.29) | 2.2 (1.5, 3.3) | 7.5 (4.5, 12.2) | 203.47 (32.90,472.06) | 0.1 (0.0, 0.1) | 0.2 (0.1, 0.4) | 192.07 (26.71,466.56) |
| Cook Islands | 0.0 (0.0, 0.0) | 0.1 (0.1, 0.1) | 111.68 (86.08,138.96) | 0.0 (0.0, 0.0) | 0.0 (0.0, 0.0) | 183.25 (63.18,369.38) | 0.0 (0.0, 0.0) | 0.0 (0.0, 0.0) | 217.47 (81.89,433.49) |
| Costa Rica | 4.4 (4.0, 4.8) | 17.9 (16.4, 19.5) | 282.50 (235.88,332.35) | 1.6 (1.2, 2.1) | 10.9 (8.0, 14.3) | 484.10 (285.91,756.65) | 0.1 (0.0, 0.1) | 0.4 (0.3, 0.6) | 552.87 (335.04,850.85) |
| Côte d'Ivoire | 15.3 (13.8, 17.1) | 58.0 (52.6, 64.8) | 240.47 (190.47,293.59) | 4.7 (3.2, 6.6) | 15.9 (10.7, 24.1) | 210.99 (71.37,426.67) | 0.1 (0.1, 0.2) | 0.5 (0.3, 0.7) | 212.72 (71.05,431.40) |
| Croatia | 145.0 (132.9, 160.1) | 206.0 (187.9, 225.0) | 38.50 (21.52,56.63) | 66.0 (46.5, 92.9) | 71.1 (51.5, 98.1) | 8.18 (-34.06,73.33) | 2.3 (1.7, 3.2) | 3.2 (2.3, 4.3) | 34.44 (-15.49,108.94) |
| Cuba | 123.1 (113.2, 134.8) | 263.2 (239.6, 286.6) | 111.32 (85.29,138.72) | 34.0 (25.5, 44.7) | 130.6 (90.3, 185.5) | 286.19 (141.46,483.67) | 1.4 (1.0, 1.8) | 4.8 (3.4, 6.6) | 254.71 (125.97,438.94) |
| Cyprus | 18.3 (16.6, 20.1) | 73.3 (66.8, 80.5) | 275.52 (226.71,327.06) | 8.0 (5.4, 11.2) | 18.6 (13.6, 25.6) | 134.93 (43.13,276.88) | 0.3 (0.2, 0.5) | 0.8 (0.6, 1.1) | 143.56 (47.90,292.01) |
| Czechia | 711.2 (649.1, 778.1) | 1172.5 (1076.1, 1281.8) | 63.62 (43.15,85.19) | 267.7 (200.0, 363.8) | 453.5 (322.0, 626.6) | 74.42 (7.93,169.51) | 10.1 (7.5, 13.5) | 17.7 (12.6, 24.1) | 78.15 (12.87,170.61) |
| Democratic People's Republic of Korea | 38.9 (35.3, 42.8) | 85.0 (77.5, 93.0) | 108.90 (80.07,139.42) | 8.0 (5.2, 12.6) | 13.3 (8.7, 19.0) | 58.52 (-15.31,197.00) | 0.3 (0.2, 0.4) | 0.5 (0.3, 0.7) | 74.10 (-9.33,229.91) |
| Democratic Republic of the Congo | 55.0 (49.6, 61.4) | 148.9 (134.7, 164.4) | 144.70 (108.37,183.32) | 15.8 (10.2, 24.8) | 33.8 (21.4, 52.5) | 92.15 (-5.35,265.77) | 0.5 (0.3, 0.7) | 1.0 (0.6, 1.6) | 89.57 (-9.20,265.41) |
| Denmark | 241.2 (220.8, 263.6) | 405.2 (371.1, 440.5) | 63.02 (43.77,83.33) | 142.1 (100.8, 198.0) | 212.5 (153.3, 280.8) | 55.88 (-3.48,145.98) | 4.9 (3.5, 6.5) | 9.0 (6.6, 11.7) | 86.94 (19.03,185.83) |
| Djibouti | 0.7 (0.6, 0.7) | 4.8 (4.3, 5.3) | 526.72 (435.95,623.07) | 0.1 (0.1, 0.2) | 1.2 (0.8, 1.8) | 622.16 (258.35,1265.05) | 0.0 (0.0, 0.0) | 0.0 (0.0, 0.1) | 650.70 (282.14,1289.92) |
| Dominica | 0.5 (0.5, 0.6) | 1.1 (1.0, 1.2) | 103.96 (78.02,131.30) | 0.2 (0.2, 0.3) | 0.6 (0.4, 0.8) | 139.32 (42.14,287.54) | 0.0 (0.0, 0.0) | 0.0 (0.0, 0.0) | 123.34 (36.20,256.70) |
| Dominican Republic | 31.8 (29.0, 35.2) | 108.1 (98.8, 118.4) | 222.14 (181.82,265.32) | 17.5 (12.2, 24.4) | 71.9 (40.9, 110.8) | 300.44 (91.49,605.14) | 0.6 (0.4, 0.8) | 2.7 (1.6, 4.3) | 318.75 (109.49,623.17) |
| Ecuador | 28.8 (26.2, 31.7) | 115.2 (105.5, 126.0) | 277.92 (229.19,329.58) | 21.2 (16.0, 27.6) | 90.1 (65.3, 121.1) | 314.66 (178.92,499.18) | 0.7 (0.5, 0.9) | 3.7 (2.7, 5.0) | 396.59 (235.57,616.90) |
| Egypt | 695.2 (635.8, 764.4) | 2570.7 (2369.5, 2807.8) | 243.57 (201.92,287.99) | 279.3 (196.6, 385.8) | 1110.7 (787.5, 1538.3) | 264.84 (126.11,474.49) | 12.1 (8.1, 17.3) | 43.2 (29.9, 60.6) | 229.39 (97.40,437.57) |
| El Salvador | 4.7 (4.3, 5.3) | 15.8 (14.4, 17.2) | 214.85 (172.96,259.29) | 2.6 (1.9, 3.5) | 11.0 (7.3, 15.7) | 302.17 (145.52,530.76) | 0.1 (0.1, 0.1) | 0.4 (0.3, 0.6) | 341.00 (172.89,584.77) |
| Equatorial Guinea | 0.6 (0.5, 0.6) | 3.7 (3.3, 4.1) | 484.47 (400.65,574.23) | 0.2 (0.1, 0.3) | 1.1 (0.7, 1.9) | 433.95 (120.43,1006.19) | 0.0 (0.0, 0.0) | 0.0 (0.0, 0.1) | 418.00 (109.73,996.55) |
| Eritrea | 4.3 (3.9, 4.8) | 14.3 (12.9, 15.8) | 194.60 (150.60,242.11) | 1.4 (0.9, 1.9) | 3.9 (2.6, 5.8) | 161.86 (46.21,338.21) | 0.0 (0.0, 0.1) | 0.1 (0.1, 0.2) | 176.21 (54.81,366.82) |
| Estonia | 22.9 (20.8, 25.0) | 29.6 (27.0, 32.1) | 29.01 (12.28,46.72) | 6.0 (4.3, 7.9) | 16.7 (12.0, 23.4) | 182.72 (75.66,334.71) | 0.2 (0.2, 0.3) | 0.6 (0.4, 0.8) | 199.71 (92.27,349.29) |
| Eswatini | 1.2 (1.1, 1.3) | 4.0 (3.7, 4.5) | 209.40 (165.68,255.73) | 0.6 (0.3, 0.9) | 2.9 (1.6, 4.7) | 407.62 (112.22,939.99) | 0.0 (0.0, 0.0) | 0.1 (0.0, 0.1) | 383.06 (105.95,875.71) |
| Ethiopia | 51.4 (46.7, 57.0) | 176.5 (161.1, 195.8) | 200.33 (157.73,245.74) | 16.9 (10.7, 24.9) | 34.4 (25.7, 46.0) | 78.05 (4.10,209.76) | 0.5 (0.3, 0.8) | 1.2 (0.9, 1.6) | 89.90 (10.81,232.91) |
| Fiji | 1.3 (1.1, 1.4) | 2.9 (2.6, 3.1) | 120.84 (91.88,151.87) | 0.2 (0.1, 0.2) | 0.4 (0.3, 0.6) | 169.51 (54.87,355.00) | 0.0 (0.0, 0.0) | 0.0 (0.0, 0.0) | 202.84 (73.01,414.55) |
| Finland | 198.8 (180.8, 217.5) | 448.4 (409.5, 490.6) | 118.58 (90.12,148.59) | 133.1 (97.7, 179.0) | 361.1 (263.7, 484.3) | 178.84 (74.76,328.14) | 4.9 (3.6, 6.5) | 14.9 (10.9, 19.7) | 206.03 (97.20,359.13) |
| France | 1686.5 (1535.9, 1847.3) | 3989.9 (3650.0, 4332.5) | 131.52 (102.01,162.65) | 1379.7 (1001.7, 1862.2) | 2109.5 (1582.1, 2736.2) | 58.54 (2.19,139.96) | 53.1 (38.5, 70.6) | 97.5 (72.7, 126.7) | 87.45 (21.86,180.94) |
| Gabon | 2.0 (1.8, 2.2) | 6.3 (5.7, 7.0) | 190.31 (148.68,234.70) | 1.1 (0.7, 1.6) | 3.6 (2.4, 5.6) | 212.09 (60.51,470.21) | 0.0 (0.0, 0.1) | 0.1 (0.1, 0.2) | 193.05 (52.11,433.16) |
| Gambia | 1.5 (1.4, 1.7) | 5.4 (4.9, 6.1) | 221.59 (175.03,271.29) | 0.7 (0.5, 1.1) | 3.5 (2.1, 5.1) | 331.54 (124.52,641.37) | 0.0 (0.0, 0.0) | 0.1 (0.1, 0.2) | 338.69 (119.69,657.51) |
| Georgia | 129.8 (118.7, 142.7) | 127.1 (116.2, 139.7) | -0.43 (-12.84,12.66) | 51.2 (36.8, 69.1) | 50.7 (35.4, 70.6) | 10.65 (-31.78,72.27) | 1.9 (1.3, 2.5) | 2.0 (1.4, 2.7) | 17.70 (-25.63,80.56) |
| Germany | 4649.4 (4206.6, 5057.8) | 10032.6 (9194.1, 10907.6) | 114.79 (88.74,142.45) | 5206.9 (3690.6, 7018.0) | 7927.3 (5881.4, 10240.8) | 59.80 (1.39,145.39) | 202.1 (144.9, 271.6) | 350.6 (257.9, 448.8) | 80.02 (16.02,172.50) |
| Ghana | 27.2 (24.5, 30.2) | 125.7 (114.4, 139.3) | 304.38 (246.94,365.33) | 10.5 (6.8, 15.9) | 52.1 (33.9, 74.8) | 354.82 (147.80,712.19) | 0.3 (0.2, 0.5) | 1.8 (1.2, 2.6) | 383.66 (163.79,785.47) |
| Greece | 101.3 (92.4, 111.0) | 221.7 (202.5, 240.4) | 118.63 (91.33,147.43) | 45.2 (32.6, 60.4) | 86.0 (65.1, 107.6) | 89.01 (24.48,180.98) | 2.1 (1.5, 2.8) | 4.3 (3.3, 5.5) | 101.00 (32.07,199.48) |
| Greenland | 2.2 (2.0, 2.5) | 3.8 (3.4, 4.2) | 67.00 (43.98,91.72) | 1.4 (1.0, 1.9) | 2.5 (1.8, 3.5) | 84.82 (11.11,196.98) | 0.0 (0.0, 0.1) | 0.1 (0.1, 0.1) | 115.87 (33.61,235.80) |
| Grenada | 0.4 (0.4, 0.5) | 1.3 (1.2, 1.4) | 191.33 (154.27,230.47) | 0.2 (0.1, 0.3) | 0.6 (0.5, 0.9) | 233.10 (105.31,424.57) | 0.0 (0.0, 0.0) | 0.0 (0.0, 0.0) | 211.99 (95.75,383.23) |
| Guam | 0.3 (0.3, 0.3) | 0.7 (0.7, 0.8) | 129.01 (100.26,159.75) | 0.1 (0.1, 0.1) | 0.3 (0.2, 0.4) | 239.01 (113.81,423.66) | 0.0 (0.0, 0.0) | 0.0 (0.0, 0.0) | 274.43 (139.70,469.86) |
| Guatemala | 5.5 (5.0, 6.1) | 26.5 (24.1, 29.2) | 342.00 (281.82,405.85) | 4.1 (3.0, 5.7) | 22.3 (15.8, 30.7) | 408.39 (219.89,685.77) | 0.1 (0.1, 0.2) | 0.7 (0.5, 1.0) | 459.97 (256.98,750.51) |
| Guinea | 11.4 (10.3, 12.5) | 28.8 (26.1, 31.9) | 131.05 (99.48,164.59) | 6.6 (4.9, 9.0) | 13.2 (8.6, 18.2) | 87.79 (10.63,197.50) | 0.2 (0.2, 0.3) | 0.4 (0.3, 0.6) | 78.84 (3.27,190.97) |
| Guinea-Bissau | 1.3 (1.2, 1.4) | 3.8 (3.5, 4.3) | 177.90 (138.41,219.83) | 0.9 (0.6, 1.4) | 1.9 (1.3, 2.7) | 90.86 (8.02,234.32) | 0.0 (0.0, 0.0) | 0.1 (0.0, 0.1) | 81.33 (1.15,221.88) |
| Guyana | 3.3 (3.0, 3.7) | 6.7 (6.1, 7.3) | 94.41 (67.41,123.42) | 2.7 (1.9, 3.7) | 6.8 (4.4, 10.0) | 145.48 (31.71,309.25) | 0.1 (0.1, 0.1) | 0.2 (0.1, 0.3) | 142.80 (31.04,302.91) |
| Haiti | 21.2 (19.2, 23.4) | 67.0 (60.8, 74.4) | 203.50 (160.37,249.40) | 13.4 (8.4, 19.6) | 29.5 (17.4, 45.9) | 111.31 (2.40,289.88) | 0.4 (0.3, 0.6) | 0.9 (0.5, 1.5) | 111.62 (0.03,299.83) |
| Honduras | 3.6 (3.2, 4.0) | 17.4 (15.9, 19.2) | 342.84 (282.08,407.20) | 1.9 (1.3, 2.7) | 10.3 (5.5, 16.5) | 403.61 (112.79,828.21) | 0.1 (0.0, 0.1) | 0.4 (0.2, 0.6) | 474.81 (144.91,953.29) |
| Hungary | 697.6 (637.9, 766.0) | 997.5 (911.9, 1092.7) | 38.68 (20.79,57.55) | 515.2 (365.8, 717.7) | 422.2 (284.3, 613.2) | -18.83 (-53.89,33.24) | 16.5 (11.9, 22.8) | 15.9 (10.8, 22.7) | -4.12 (-43.67,54.28) |
| Iceland | 13.6 (12.5, 14.9) | 29.1 (26.8, 31.6) | 103.31 (78.93,128.97) | 2.3 (1.7, 2.9) | 5.1 (3.9, 6.7) | 110.56 (38.81,207.62) | 0.1 (0.1, 0.1) | 0.2 (0.2, 0.3) | 141.19 (58.30,255.43) |
| India | 5659.5 (5163.1, 6247.2) | 22484.0 (20542.0, 24589.4) | 270.93 (222.40,322.22) | 1209.0 (886.5, 1651.0) | 5088.1 (3669.9, 6903.1) | 289.94 (144.81,504.09) | 36.7 (26.6, 51.3) | 177.3 (126.0, 241.5) | 346.57 (175.98,603.69) |
| Indonesia | 248.9 (227.0, 276.1) | 782.9 (712.5, 857.9) | 194.87 (155.27,236.78) | 65.5 (45.7, 96.4) | 226.9 (160.3, 313.0) | 220.15 (90.86,427.40) | 2.1 (1.4, 3.2) | 8.2 (5.6, 11.2) | 254.95 (103.98,528.16) |
| Iran (Islamic Republic of) | 465.1 (426.4, 512.7) | 2707.8 (2495.9, 2950.9) | 445.41 (379.38,515.82) | 64.0 (44.7, 89.4) | 340.8 (260.5, 442.7) | 410.05 (221.57,691.19) | 2.4 (1.6, 3.4) | 14.8 (11.0, 19.2) | 496.50 (266.88,880.42) |
| Iraq | 86.0 (78.3, 94.9) | 570.9 (523.1, 623.2) | 502.78 (427.14,583.54) | 10.9 (7.5, 15.3) | 60.1 (39.2, 85.3) | 379.05 (167.62,699.49) | 0.4 (0.3, 0.6) | 2.3 (1.5, 3.2) | 375.97 (157.98,746.24) |
| Ireland | 123.6 (113.0, 135.1) | 366.3 (336.1, 397.7) | 179.89 (144.81,216.85) | 32.6 (24.1, 42.6) | 106.0 (80.4, 135.5) | 248.62 (131.28,412.88) | 1.3 (1.0, 1.7) | 4.2 (3.1, 5.3) | 235.68 (121.85,395.80) |
| Israel | 150.7 (137.4, 165.1) | 512.6 (471.3, 559.0) | 229.06 (188.33,272.04) | 49.7 (35.9, 66.2) | 109.5 (82.7, 138.6) | 128.89 (51.00,239.62) | 2.1 (1.5, 2.9) | 5.1 (3.8, 6.4) | 143.13 (58.47,265.25) |
| Italy | 2704.8 (2473.5, 2951.9) | 4517.2 (4152.4, 4891.7) | 60.67 (41.49,80.87) | 2597.0 (1932.8, 3373.3) | 1513.7 (1133.2, 1863.6) | -41.06 (-59.89,-15.41) | 111.3 (82.9, 142.8) | 82.6 (60.8, 102.4) | -25.82 (-49.80,6.58) |
| Jamaica | 15.9 (14.5, 17.4) | 45.4 (41.5, 49.7) | 173.82 (140.01,209.47) | 2.8 (2.0, 3.7) | 8.2 (5.5, 11.7) | 184.08 (69.44,344.83) | 0.1 (0.1, 0.1) | 0.3 (0.2, 0.5) | 179.52 (73.26,332.31) |
| Japan | 953.7 (874.5, 1041.0) | 1897.8 (1738.6, 2065.6) | 94.69 (71.01,119.61) | 462.0 (368.7, 579.4) | 618.7 (467.0, 766.6) | 30.95 (-7.96,80.64) | 19.0 (15.0, 23.6) | 38.1 (27.3, 48.3) | 92.21 (32.15,169.76) |
| Jordan | 33.4 (30.4, 37.1) | 333.3 (306.3, 364.4) | 754.22 (644.40,872.65) | 2.8 (1.9, 4.0) | 20.6 (14.2, 28.8) | 494.43 (252.26,879.64) | 0.1 (0.1, 0.2) | 0.8 (0.6, 1.1) | 511.16 (250.96,938.72) |
| Kazakhstan | 203.2 (185.1, 224.0) | 378.3 (344.9, 416.6) | 73.68 (50.80,97.86) | 73.3 (55.6, 95.1) | 294.9 (207.7, 422.1) | 297.76 (152.55,492.23) | 2.6 (2.0, 3.4) | 10.4 (7.4, 14.6) | 289.06 (149.64,479.34) |
| Kenya | 30.0 (27.1, 33.1) | 139.4 (126.7, 153.9) | 312.27 (255.14,372.75) | 9.1 (5.3, 15.6) | 58.5 (39.7, 88.9) | 480.32 (159.08,1212.17) | 0.3 (0.2, 0.5) | 2.0 (1.3, 3.1) | 468.30 (154.37,1197.85) |
| Kiribati | 0.1 (0.1, 0.1) | 0.2 (0.2, 0.2) | 128.08 (96.52,161.53) | 0.0 (0.0, 0.0) | 0.1 (0.0, 0.1) | 109.17 (16.55,254.07) | 0.0 (0.0, 0.0) | 0.0 (0.0, 0.0) | 110.20 (19.56,252.05) |
| Kuwait | 38.9 (35.4, 43.5) | 227.2 (207.6, 249.9) | 414.51 (345.95,488.81) | 2.1 (1.6, 2.8) | 10.3 (7.1, 13.9) | 296.14 (159.77,479.28) | 0.1 (0.1, 0.1) | 0.4 (0.3, 0.6) | 388.25 (225.23,607.01) |
| Kyrgyzstan | 44.7 (40.6, 49.2) | 102.2 (93.2, 112.8) | 113.92 (84.96,144.54) | 17.9 (13.0, 23.9) | 43.7 (29.4, 64.3) | 132.24 (35.48,267.47) | 0.6 (0.4, 0.8) | 1.3 (0.9, 1.9) | 103.90 (20.21,223.61) |
| Lao People's Democratic Republic | 2.5 (2.3, 2.8) | 8.9 (8.1, 9.9) | 235.75 (189.98,284.22) | 0.9 (0.6, 1.3) | 2.0 (1.4, 2.8) | 113.25 (16.80,276.43) | 0.0 (0.0, 0.0) | 0.1 (0.0, 0.1) | 119.45 (17.64,298.25) |
| Latvia | 41.8 (38.2, 45.5) | 45.5 (41.8, 49.8) | 12.47 (-1.28,26.94) | 10.9 (8.0, 14.7) | 25.2 (17.6, 35.3) | 134.34 (45.52,262.46) | 0.4 (0.3, 0.5) | 0.9 (0.6, 1.2) | 137.45 (53.55,255.85) |
| Lebanon | 101.4 (93.4, 111.0) | 464.5 (429.5, 504.5) | 328.26 (279.32,380.21) | 12.7 (8.3, 18.3) | 46.3 (32.9, 62.9) | 231.74 (96.53,454.62) | 0.5 (0.4, 0.8) | 2.5 (1.7, 3.6) | 325.33 (137.53,636.25) |
| Lesotho | 2.5 (2.2, 2.7) | 5.7 (5.2, 6.3) | 119.30 (89.24,151.12) | 0.9 (0.5, 1.7) | 4.1 (2.3, 6.6) | 342.45 (69.68,1139.35) | 0.0 (0.0, 0.1) | 0.1 (0.1, 0.2) | 295.54 (48.76,1114.22) |
| Liberia | 4.0 (3.6, 4.4) | 13.7 (12.4, 15.2) | 212.99 (168.17,260.69) | 2.4 (1.5, 3.5) | 6.5 (4.5, 9.5) | 148.29 (36.41,338.21) | 0.1 (0.1, 0.1) | 0.2 (0.1, 0.3) | 122.09 (22.65,290.15) |
| Libya | 20.2 (18.3, 22.2) | 170.5 (156.7, 187.2) | 653.81 (554.85,758.47) | 2.8 (1.6, 5.4) | 31.9 (20.6, 49.5) | 916.44 (344.79,2635.11) | 0.1 (0.1, 0.2) | 1.2 (0.8, 2.0) | 876.58 (276.70,2973.26) |
| Lithuania | 59.4 (54.1, 65.4) | 77.5 (70.8, 84.4) | 29.28 (12.54,46.97) | 14.0 (10.2, 19.2) | 48.4 (34.2, 68.4) | 239.49 (109.61,431.31) | 0.5 (0.3, 0.6) | 1.7 (1.2, 2.3) | 250.64 (122.90,433.65) |
| Luxembourg | 14.4 (13.1, 15.8) | 44.9 (40.9, 49.3) | 192.11 (155.21,231.37) | 13.5 (9.7, 18.3) | 20.9 (15.1, 27.9) | 59.21 (-0.29,147.32) | 0.5 (0.4, 0.7) | 0.9 (0.6, 1.2) | 80.86 (16.00,174.73) |
| Madagascar | 16.3 (14.8, 18.0) | 55.1 (49.8, 60.9) | 195.63 (154.13,239.47) | 4.3 (3.0, 6.2) | 11.6 (7.5, 18.0) | 139.89 (18.44,327.15) | 0.1 (0.1, 0.2) | 0.4 (0.2, 0.6) | 122.84 (4.40,314.43) |
| Malawi | 12.7 (11.5, 14.0) | 35.5 (32.0, 39.3) | 149.68 (114.62,186.82) | 4.5 (3.2, 6.2) | 13.0 (9.2, 18.1) | 162.42 (56.74,319.17) | 0.1 (0.1, 0.2) | 0.4 (0.3, 0.6) | 160.16 (53.90,324.99) |
| Malaysia | 32.6 (29.6, 35.9) | 144.7 (132.6, 158.2) | 457.06 (384.72,533.56) | 3.1 (2.4, 4.1) | 23.2 (17.5, 31.0) | 826.17 (509.41,1259.94) | 0.1 (0.1, 0.1) | 0.9 (0.7, 1.2) | 898.52 (554.65,1376.48) |
| Maldives | 0.2 (0.2, 0.2) | 1.7 (1.6, 1.9) | 770.19 (644.88,904.12) | 0.0 (0.0, 0.0) | 0.1 (0.1, 0.2) | 336.50 (177.38,572.11) | 0.0 (0.0, 0.0) | 0.0 (0.0, 0.0) | 411.48 (215.51,705.78) |
| Mali | 13.8 (12.5, 15.3) | 43.9 (39.8, 48.8) | 188.83 (147.79,232.35) | 6.6 (4.8, 9.0) | 19.2 (13.6, 27.0) | 171.00 (64.05,326.77) | 0.2 (0.2, 0.3) | 0.6 (0.5, 0.9) | 168.57 (61.72,326.89) |
| Malta | 10.0 (9.1, 10.9) | 25.7 (23.4, 28.0) | 148.15 (116.31,181.73) | 3.0 (2.2, 4.1) | 6.6 (4.9, 8.8) | 110.12 (34.18,220.54) | 0.1 (0.1, 0.2) | 0.3 (0.2, 0.4) | 133.11 (48.46,256.55) |
| Marshall Islands | 0.0 (0.0, 0.0) | 0.1 (0.1, 0.1) | 200.83 (159.26,245.11) | 0.0 (0.0, 0.0) | 0.0 (0.0, 0.0) | 211.82 (50.39,478.55) | 0.0 (0.0, 0.0) | 0.0 (0.0, 0.0) | 210.33 (57.12,474.57) |
| Mauritania | 4.9 (4.5, 5.4) | 17.6 (16.1, 19.4) | 226.27 (180.72,274.51) | 3.5 (1.7, 6.7) | 8.1 (5.3, 12.1) | 105.75 (-11.55,517.83) | 0.1 (0.1, 0.2) | 0.3 (0.2, 0.4) | 110.53 (-9.04,536.99) |
| Mauritius | 1.8 (1.6, 2.0) | 5.1 (4.6, 5.5) | 168.30 (133.96,205.08) | 0.4 (0.3, 0.5) | 0.7 (0.5, 0.9) | 79.82 (12.39,170.51) | 0.0 (0.0, 0.0) | 0.0 (0.0, 0.0) | 85.97 (16.96,179.49) |
| Mexico | 34.4 (31.6, 37.7) | 116.2 (106.6, 127.1) | 222.03 (181.88,264.29) | 30.3 (21.8, 41.2) | 135.2 (99.8, 179.0) | 305.06 (161.05,515.17) | 1.0 (0.7, 1.3) | 4.6 (3.4, 6.0) | 333.55 (184.22,547.43) |
| Micronesia (Federated States of) | 0.1 (0.1, 0.1) | 0.2 (0.2, 0.2) | 103.63 (75.45,133.33) | 0.0 (0.0, 0.0) | 0.1 (0.0, 0.1) | 97.73 (5.05,248.12) | 0.0 (0.0, 0.0) | 0.0 (0.0, 0.0) | 99.85 (6.58,245.68) |
| Monaco | 2.1 (1.9, 2.2) | 3.8 (3.4, 4.1) | 79.25 (56.83,102.88) | 1.7 (1.2, 2.4) | 3.3 (2.4, 4.4) | 97.65 (20.08,221.02) | 0.1 (0.1, 0.1) | 0.2 (0.1, 0.2) | 102.94 (26.06,220.12) |
| Mongolia | 12.2 (11.1, 13.5) | 40.4 (36.5, 44.8) | 225.34 (181.36,271.83) | 12.0 (8.8, 16.4) | 46.1 (33.9, 62.2) | 272.50 (136.66,471.35) | 0.4 (0.3, 0.6) | 1.7 (1.2, 2.3) | 284.43 (139.66,499.42) |
| Montenegro | 13.8 (12.6, 15.1) | 25.3 (23.1, 27.6) | 79.23 (56.91,102.83) | 3.1 (2.3, 4.3) | 7.2 (5.1, 9.9) | 138.51 (48.74,267.75) | 0.1 (0.1, 0.2) | 0.3 (0.2, 0.4) | 167.88 (62.98,323.17) |
| Morocco | 191.9 (175.6, 211.6) | 846.1 (775.0, 922.8) | 313.88 (261.46,369.24) | 13.9 (8.7, 21.3) | 85.1 (53.8, 131.1) | 461.30 (163.82,979.14) | 0.6 (0.3, 0.9) | 3.7 (2.3, 5.5) | 491.25 (184.94,1080.00) |
| Mozambique | 15.5 (14.1, 17.2) | 39.0 (35.2, 43.4) | 128.20 (95.27,163.35) | 6.8 (4.7, 9.7) | 18.4 (10.9, 29.8) | 156.35 (22.29,356.05) | 0.3 (0.2, 0.4) | 0.7 (0.4, 1.2) | 146.72 (13.78,346.03) |
| Myanmar | 42.9 (39.0, 47.3) | 110.8 (101.1, 121.7) | 151.40 (117.98,186.74) | 7.4 (4.5, 12.2) | 17.5 (10.1, 26.7) | 123.56 (8.23,368.07) | 0.2 (0.1, 0.4) | 0.5 (0.3, 0.8) | 140.53 (19.73,396.23) |
| Namibia | 2.6 (2.4, 2.9) | 8.6 (7.9, 9.6) | 200.59 (158.12,246.14) | 0.7 (0.4, 1.2) | 2.4 (1.5, 3.9) | 227.45 (40.52,699.91) | 0.0 (0.0, 0.0) | 0.1 (0.1, 0.1) | 228.61 (50.85,673.36) |
| Nauru | 0.0 (0.0, 0.0) | 0.0 (0.0, 0.0) | 38.27 (18.87,58.90) | 0.0 (0.0, 0.0) | 0.0 (0.0, 0.0) | 17.43 (-49.98,133.50) | 0.0 (0.0, 0.0) | 0.0 (0.0, 0.0) | 17.72 (-47.85,128.10) |
| Nepal | 104.0 (94.5, 115.1) | 358.4 (328.9, 396.0) | 225.92 (182.06,272.35) | 19.1 (11.7, 32.2) | 96.9 (64.4, 139.1) | 375.39 (144.14,895.43) | 0.6 (0.3, 1.0) | 3.3 (2.2, 4.8) | 434.90 (163.74,1075.33) |
| Netherlands | 1035.7 (951.4, 1129.1) | 2172.2 (1987.7, 2356.8) | 106.92 (81.59,133.51) | 285.3 (210.1, 381.2) | 626.0 (467.7, 802.1) | 125.93 (47.19,237.98) | 11.3 (8.3, 15.0) | 29.4 (21.8, 37.6) | 162.08 (71.84,289.34) |
| New Zealand | 156.2 (142.7, 171.5) | 394.9 (361.1, 429.3) | 136.89 (106.94,168.47) | 34.3 (27.1, 43.5) | 109.1 (90.5, 127.9) | 201.33 (124.58,302.33) | 1.3 (1.0, 1.6) | 4.6 (3.8, 5.4) | 234.38 (151.30,341.91) |
| Nicaragua | 2.7 (2.4, 2.9) | 13.1 (12.0, 14.4) | 357.31 (297.09,421.00) | 1.0 (0.7, 1.3) | 6.9 (4.8, 9.9) | 627.75 (353.78,1023.12) | 0.0 (0.0, 0.0) | 0.2 (0.2, 0.3) | 642.47 (366.82,1049.19) |
| Niger | 9.7 (8.8, 10.8) | 33.2 (30.0, 36.7) | 212.77 (166.49,262.20) | 4.0 (2.3, 6.8) | 9.4 (5.7, 16.1) | 116.11 (-15.12,400.10) | 0.1 (0.1, 0.2) | 0.3 (0.2, 0.5) | 123.79 (-12.22,398.70) |
| Nigeria | 186.7 (171.1, 206.0) | 604.5 (551.6, 668.8) | 189.83 (150.46,231.40) | 56.3 (32.5, 91.9) | 138.0 (89.6, 205.9) | 122.93 (11.64,365.89) | 2.1 (1.2, 3.4) | 4.8 (3.3, 7.0) | 111.76 (9.58,330.32) |
| Niue | 0.0 (0.0, 0.0) | 0.0 (0.0, 0.0) | 28.98 (12.48,46.40) | 0.0 (0.0, 0.0) | 0.0 (0.0, 0.0) | 47.54 (-22.66,151.65) | 0.0 (0.0, 0.0) | 0.0 (0.0, 0.0) | 48.47 (-20.33,152.59) |
| North Macedonia | 39.7 (36.1, 43.8) | 89.5 (81.4, 97.7) | 116.54 (87.87,146.83) | 10.6 (7.8, 14.0) | 24.6 (17.0, 35.0) | 130.72 (47.44,248.10) | 0.4 (0.3, 0.5) | 1.0 (0.7, 1.5) | 153.55 (57.60,295.92) |
| Northern Mariana Islands | 0.1 (0.1, 0.1) | 0.2 (0.2, 0.3) | 83.30 (58.36,110.46) | 0.1 (0.0, 0.1) | 0.1 (0.1, 0.2) | 113.03 (17.20,280.21) | 0.0 (0.0, 0.0) | 0.0 (0.0, 0.0) | 165.82 (52.03,353.55) |
| Norway | 253.3 (231.4, 277.1) | 568.8 (522.4, 620.0) | 112.82 (86.15,140.93) | 64.2 (48.8, 83.4) | 139.0 (110.9, 171.6) | 115.34 (48.29,204.66) | 2.6 (2.0, 3.3) | 6.3 (5.0, 7.7) | 137.40 (65.25,232.85) |
| Oman | 14.9 (13.5, 16.6) | 128.7 (116.7, 143.2) | 640.67 (531.79,756.93) | 1.2 (0.7, 1.9) | 9.9 (6.8, 14.8) | 705.16 (314.71,1553.07) | 0.0 (0.0, 0.1) | 0.4 (0.2, 0.5) | 673.53 (305.18,1501.82) |
| Pakistan | 604.8 (552.6, 667.6) | 2087.1 (1900.7, 2307.2) | 217.21 (173.63,263.13) | 124.7 (69.9, 232.0) | 397.4 (277.2, 550.8) | 198.61 (47.02,666.02) | 4.1 (2.3, 7.9) | 12.4 (8.3, 17.2) | 183.27 (36.01,675.77) |
| Palau | 0.0 (0.0, 0.0) | 0.1 (0.1, 0.1) | 167.07 (131.67,204.46) | 0.0 (0.0, 0.0) | 0.0 (0.0, 0.1) | 237.43 (84.55,491.95) | 0.0 (0.0, 0.0) | 0.0 (0.0, 0.0) | 238.78 (84.63,485.64) |
| Palestine | 11.5 (10.5, 12.6) | 70.8 (64.8, 77.7) | 460.07 (387.04,537.12) | 1.6 (1.0, 2.8) | 5.8 (4.4, 7.9) | 211.87 (75.76,562.56) | 0.1 (0.0, 0.1) | 0.2 (0.2, 0.3) | 194.43 (61.57,539.39) |
| Panama | 4.8 (4.4, 5.3) | 18.3 (16.7, 20.1) | 257.70 (211.93,306.68) | 1.2 (0.9, 1.6) | 5.5 (3.8, 7.8) | 320.94 (174.80,524.22) | 0.0 (0.0, 0.1) | 0.2 (0.1, 0.3) | 337.95 (184.14,554.96) |
| Papua New Guinea | 3.5 (3.2, 3.9) | 12.2 (11.1, 13.5) | 230.08 (182.67,280.40) | 0.4 (0.2, 0.7) | 0.9 (0.5, 1.6) | 127.80 (-17.43,567.01) | 0.0 (0.0, 0.0) | 0.0 (0.0, 0.0) | 126.27 (-22.02,594.24) |
| Paraguay | 10.9 (9.9, 12.1) | 32.0 (29.2, 35.0) | 165.22 (130.44,201.99) | 1.5 (1.1, 2.0) | 6.3 (4.1, 9.0) | 311.13 (141.48,553.33) | 0.0 (0.0, 0.1) | 0.2 (0.1, 0.3) | 328.05 (157.54,565.89) |
| Peru | 47.9 (43.7, 52.6) | 178.5 (162.6, 195.0) | 255.40 (208.91,304.47) | 37.2 (26.3, 51.2) | 156.7 (99.6, 224.6) | 326.07 (154.84,584.46) | 1.3 (0.9, 1.7) | 6.2 (4.0, 9.0) | 408.72 (204.48,719.00) |
| Philippines | 49.3 (45.0, 54.4) | 141.6 (129.3, 156.0) | 169.14 (133.37,206.95) | 8.2 (5.8, 11.7) | 25.0 (19.1, 31.6) | 184.74 (92.93,338.28) | 0.3 (0.2, 0.4) | 0.9 (0.7, 1.1) | 213.52 (108.54,401.08) |
| Poland | 371.0 (339.4, 404.6) | 632.7 (577.4, 686.9) | 69.64 (49.75,90.58) | 72.0 (52.4, 96.8) | 257.2 (188.0, 347.3) | 258.57 (125.18,450.93) | 2.8 (2.0, 3.8) | 9.1 (6.8, 12.1) | 219.75 (104.51,386.95) |
| Portugal | 217.7 (197.9, 238.6) | 505.4 (462.2, 554.1) | 125.71 (95.93,157.15) | 215.0 (150.8, 293.5) | 235.5 (174.4, 305.3) | 13.89 (-28.09,76.28) | 8.0 (5.5, 10.8) | 10.4 (7.7, 13.3) | 33.58 (-14.96,105.32) |
| Puerto Rico | 46.2 (42.3, 50.6) | 96.3 (89.0, 104.3) | 105.47 (79.99,132.32) | 39.1 (28.5, 51.8) | 65.2 (46.7, 85.9) | 63.11 (9.36,137.77) | 1.4 (1.1, 1.9) | 2.9 (2.1, 3.8) | 92.47 (29.51,179.10) |
| Qatar | 8.2 (7.3, 9.2) | 139.8 (126.0, 156.5) | 1452.75 (1213.82,1707.36) | 1.0 (0.7, 1.4) | 15.2 (10.1, 23.3) | 1242.08 (656.94,2119.91) | 0.0 (0.0, 0.0) | 0.5 (0.3, 0.8) | 1173.08 (616.48,2013.59) |
| Republic of Korea | 364.2 (330.7, 403.6) | 1616.0 (1472.0, 1765.2) | 319.01 (264.99,376.92) | 232.5 (169.2, 309.7) | 557.0 (393.9, 783.3) | 112.65 (30.65,228.56) | 8.5 (6.2, 11.7) | 27.1 (19.1, 39.1) | 178.74 (67.21,342.48) |
| Republic of Moldova | 43.8 (39.9, 48.3) | 71.4 (64.7, 78.1) | 60.47 (38.90,83.27) | 63.2 (42.3, 92.2) | 108.5 (74.7, 154.6) | 69.26 (-4.69,190.86) | 2.1 (1.4, 3.0) | 3.8 (2.6, 5.2) | 74.59 (1.35,192.54) |
| Romania | 127.6 (115.7, 138.9) | 174.6 (158.6, 191.2) | 37.98 (20.33,56.59) | 55.8 (38.5, 79.4) | 136.3 (95.5, 193.7) | 142.43 (43.58,299.14) | 2.0 (1.4, 2.8) | 5.4 (3.7, 7.5) | 172.11 (61.59,344.55) |
| Russian Federation | 2099.8 (1916.5, 2293.7) | 3078.5 (2810.6, 3346.7) | 43.90 (26.10,62.65) | 439.5 (322.2, 586.0) | 2510.8 (1821.3, 3381.0) | 471.83 (261.39,770.94) | 15.6 (11.5, 20.7) | 78.7 (57.8, 106.5) | 400.23 (222.97,651.03) |
| Rwanda | 7.9 (7.1, 8.7) | 26.0 (23.5, 28.9) | 189.23 (148.33,232.58) | 4.2 (2.9, 5.7) | 9.8 (6.5, 15.0) | 102.49 (4.95,240.23) | 0.1 (0.1, 0.2) | 0.3 (0.2, 0.5) | 111.36 (10.63,257.42) |
| Saint Kitts and Nevis | 0.3 (0.3, 0.3) | 1.1 (1.0, 1.2) | 240.95 (196.42,287.99) | 0.2 (0.2, 0.3) | 0.6 (0.4, 0.9) | 173.21 (63.88,326.43) | 0.0 (0.0, 0.0) | 0.0 (0.0, 0.0) | 145.51 (49.49,283.83) |
| Saint Lucia | 0.7 (0.6, 0.7) | 3.1 (2.8, 3.3) | 342.27 (287.52,400.71) | 0.4 (0.3, 0.5) | 1.7 (1.2, 2.4) | 298.91 (138.71,529.26) | 0.0 (0.0, 0.0) | 0.1 (0.0, 0.1) | 301.88 (144.55,532.04) |
| Saint Vincent and the Grenadines | 0.5 (0.5, 0.6) | 1.6 (1.4, 1.7) | 177.68 (141.01,216.44) | 0.2 (0.1, 0.2) | 0.8 (0.5, 1.0) | 319.17 (160.24,546.06) | 0.0 (0.0, 0.0) | 0.0 (0.0, 0.0) | 296.95 (148.43,512.09) |
| Samoa | 0.2 (0.2, 0.2) | 0.4 (0.4, 0.4) | 85.63 (61.52,111.10) | 0.1 (0.0, 0.1) | 0.1 (0.1, 0.1) | 79.51 (-1.36,215.94) | 0.0 (0.0, 0.0) | 0.0 (0.0, 0.0) | 84.50 (2.82,221.89) |
| San Marino | 1.9 (1.8, 2.1) | 4.8 (4.4, 5.2) | 144.44 (114.58,175.91) | 1.6 (1.1, 2.2) | 2.7 (1.6, 4.1) | 137.63 (40.14,286.43) | 0.1 (0.1, 0.1) | 0.1 (0.1, 0.2) | 160.63 (54.43,324.05) |
| Sao Tome and Principe | 0.3 (0.2, 0.3) | 0.9 (0.8, 1.0) | 213.28 (170.51,258.91) | 0.1 (0.1, 0.2) | 0.4 (0.2, 0.6) | 174.67 (28.13,412.31) | 0.0 (0.0, 0.0) | 0.0 (0.0, 0.0) | 146.51 (12.61,369.78) |
| Saudi Arabia | 100.8 (91.8, 111.5) | 993.4 (906.5, 1101.7) | 783.34 (664.19,909.37) | 20.8 (11.3, 39.4) | 201.5 (132.2, 289.9) | 749.40 (307.01,2200.40) | 0.8 (0.4, 1.6) | 6.8 (4.6, 9.5) | 631.38 (243.33,1995.79) |
| Senegal | 13.7 (12.5, 15.1) | 52.3 (47.5, 58.0) | 245.17 (196.82,296.45) | 4.8 (3.5, 6.5) | 15.2 (10.0, 21.7) | 191.37 (69.85,367.17) | 0.2 (0.1, 0.2) | 0.5 (0.3, 0.8) | 199.11 (72.81,376.00) |
| Serbia | 293.8 (267.1, 322.8) | 437.2 (400.5, 474.8) | 45.92 (27.72,65.36) | 63.7 (45.3, 86.4) | 95.2 (69.4, 133.0) | 46.06 (-9.74,129.18) | 2.3 (1.6, 3.1) | 4.0 (2.9, 5.6) | 70.38 (6.29,165.53) |
| Seychelles | 0.1 (0.1, 0.1) | 0.4 (0.3, 0.4) | 175.99 (141.34,211.94) | 0.0 (0.0, 0.1) | 0.1 (0.1, 0.1) | 159.16 (63.99,287.96) | 0.0 (0.0, 0.0) | 0.0 (0.0, 0.0) | 149.19 (58.13,269.67) |
| Sierra Leone | 6.5 (5.9, 7.2) | 19.0 (17.2, 21.1) | 159.77 (121.45,200.71) | 3.0 (1.6, 5.2) | 5.9 (4.0, 8.6) | 81.54 (-10.71,322.43) | 0.1 (0.1, 0.2) | 0.2 (0.1, 0.3) | 66.78 (-18.96,296.01) |
| Singapore | 7.5 (6.8, 8.3) | 30.0 (27.4, 32.8) | 281.95 (232.52,334.81) | 1.0 (0.8, 1.4) | 2.8 (2.1, 3.7) | 153.56 (64.69,278.24) | 0.0 (0.0, 0.1) | 0.1 (0.1, 0.2) | 213.78 (98.44,378.44) |
| Slovakia | 103.8 (95.0, 113.3) | 195.7 (179.2, 213.9) | 87.34 (63.47,112.18) | 53.9 (38.7, 73.8) | 89.4 (61.7, 127.6) | 71.94 (2.41,173.75) | 1.9 (1.4, 2.5) | 3.3 (2.3, 4.7) | 82.25 (12.76,181.38) |
| Slovenia | 66.2 (60.6, 72.5) | 121.4 (110.2, 132.4) | 80.00 (57.01,104.30) | 36.2 (25.8, 50.6) | 45.0 (32.2, 60.7) | 28.76 (-20.22,103.16) | 1.3 (0.9, 1.7) | 2.1 (1.5, 2.9) | 71.38 (8.32,164.46) |
| Solomon Islands | 0.2 (0.2, 0.3) | 0.8 (0.7, 0.9) | 216.82 (172.43,264.10) | 0.1 (0.0, 0.1) | 0.2 (0.1, 0.3) | 254.95 (58.18,810.58) | 0.0 (0.0, 0.0) | 0.0 (0.0, 0.0) | 247.82 (60.13,814.97) |
| Somalia | 9.7 (8.8, 10.7) | 27.9 (25.1, 30.9) | 163.77 (125.21,205.26) | 3.9 (2.5, 5.7) | 9.0 (5.8, 13.3) | 117.34 (12.08,295.53) | 0.1 (0.1, 0.2) | 0.3 (0.2, 0.4) | 121.44 (12.53,309.77) |
| South Africa | 111.0 (101.8, 122.2) | 407.2 (372.4, 445.9) | 237.33 (193.81,283.25) | 32.3 (24.1, 43.8) | 156.6 (124.5, 198.2) | 329.21 (189.36,526.15) | 1.0 (0.7, 1.4) | 5.3 (4.2, 6.6) | 380.79 (222.13,642.08) |
| South Sudan | 9.1 (8.2, 10.1) | 18.3 (16.5, 20.2) | 82.71 (56.66,110.33) | 3.0 (2.0, 4.5) | 7.1 (4.8, 10.5) | 114.54 (11.98,285.41) | 0.1 (0.1, 0.2) | 0.2 (0.2, 0.3) | 99.48 (6.98,256.20) |
| Spain | 1299.7 (1183.7, 1416.9) | 3070.1 (2806.9, 3338.4) | 128.34 (99.89,158.32) | 1056.2 (759.2, 1407.6) | 1186.3 (879.0, 1529.8) | 14.73 (-25.49,72.23) | 43.1 (30.2, 58.3) | 56.4 (41.3, 71.3) | 32.71 (-14.35,101.21) |
| Sri Lanka | 39.2 (35.9, 43.3) | 99.9 (91.2, 108.5) | 137.97 (107.30,170.38) | 5.2 (3.6, 7.7) | 8.9 (5.1, 13.8) | 58.33 (-12.85,174.89) | 0.2 (0.1, 0.3) | 0.4 (0.2, 0.6) | 90.07 (4.79,231.47) |
| Sudan | 78.0 (70.7, 86.1) | 355.0 (322.5, 391.7) | 305.46 (248.20,366.41) | 11.5 (6.6, 21.8) | 46.8 (27.5, 85.1) | 268.55 (16.46,915.88) | 0.4 (0.2, 0.9) | 1.8 (1.0, 3.3) | 266.10 (0.45,1083.84) |
| Suriname | 2.3 (2.1, 2.6) | 7.4 (6.7, 8.0) | 208.20 (167.38,251.35) | 1.5 (1.0, 2.0) | 4.6 (2.9, 6.7) | 235.36 (100.76,439.76) | 0.0 (0.0, 0.1) | 0.2 (0.1, 0.2) | 249.19 (114.75,446.40) |
| Sweden | 633.0 (580.7, 689.0) | 1060.5 (976.5, 1147.6) | 64.66 (45.27,85.06) | 176.8 (131.9, 229.8) | 251.6 (175.5, 345.9) | 52.49 (-5.41,130.48) | 7.5 (5.6, 9.6) | 12.1 (8.3, 16.7) | 68.89 (4.31,156.78) |
| Switzerland | 293.0 (267.2, 320.4) | 588.4 (534.3, 639.1) | 92.86 (68.98,118.04) | 135.9 (100.9, 179.2) | 183.1 (137.4, 235.1) | 38.19 (-9.69,105.10) | 5.1 (3.8, 6.7) | 8.5 (6.4, 11.0) | 68.87 (11.72,147.25) |
| Syrian Arab Republic | 73.4 (67.0, 81.1) | 355.9 (326.9, 390.2) | 353.51 (295.64,415.24) | 9.8 (6.8, 14.4) | 54.8 (36.2, 79.1) | 415.19 (179.13,793.75) | 0.4 (0.3, 0.6) | 2.3 (1.5, 3.3) | 449.29 (193.66,889.83) |
| Taiwan (Province of China) | 44.9 (40.8, 49.3) | 132.0 (120.1, 144.5) | 181.83 (145.66,220.02) | 8.9 (6.9, 11.4) | 32.1 (24.2, 41.7) | 244.00 (136.21,386.18) | 0.3 (0.2, 0.4) | 1.4 (1.0, 1.8) | 330.04 (186.39,526.31) |
| Tajikistan | 34.5 (31.4, 37.9) | 114.6 (104.1, 127.0) | 205.56 (163.74,249.94) | 8.9 (6.6, 12.0) | 22.9 (15.7, 33.5) | 139.06 (35.40,281.72) | 0.3 (0.2, 0.4) | 0.7 (0.5, 1.0) | 121.41 (27.48,259.22) |
| Thailand | 76.5 (69.4, 84.0) | 262.3 (238.8, 285.3) | 229.20 (185.89,274.95) | 25.4 (18.2, 34.5) | 122.5 (84.6, 172.2) | 350.92 (183.19,598.20) | 0.9 (0.6, 1.2) | 4.8 (3.3, 6.8) | 406.73 (213.75,698.86) |
| Timor-Leste | 0.6 (0.6, 0.7) | 1.6 (1.5, 1.8) | 145.43 (112.93,180.67) | 0.1 (0.0, 0.1) | 0.2 (0.1, 0.3) | 154.46 (20.21,418.08) | 0.0 (0.0, 0.0) | 0.0 (0.0, 0.0) | 206.68 (42.77,542.47) |
| Togo | 4.8 (4.3, 5.3) | 22.5 (20.4, 25.0) | 321.77 (260.78,386.54) | 1.4 (1.0, 2.0) | 7.1 (4.7, 10.3) | 335.14 (136.59,616.78) | 0.0 (0.0, 0.1) | 0.2 (0.2, 0.3) | 336.56 (137.72,614.69) |
| Tokelau | 0.0 (0.0, 0.0) | 0.0 (0.0, 0.0) | 64.94 (44.43,86.57) | 0.0 (0.0, 0.0) | 0.0 (0.0, 0.0) | 49.38 (-18.80,182.66) | 0.0 (0.0, 0.0) | 0.0 (0.0, 0.0) | 54.61 (-18.44,208.42) |
| Tonga | 0.1 (0.1, 0.1) | 0.2 (0.2, 0.3) | 70.70 (48.99,93.59) | 0.1 (0.1, 0.1) | 0.2 (0.1, 0.2) | 78.95 (0.19,206.84) | 0.0 (0.0, 0.0) | 0.0 (0.0, 0.0) | 91.14 (7.90,228.26) |
| Trinidad and Tobago | 8.6 (7.9, 9.5) | 25.8 (23.5, 28.1) | 192.36 (154.18,232.67) | 4.0 (2.8, 5.3) | 11.5 (7.7, 16.7) | 180.78 (73.96,336.27) | 0.1 (0.1, 0.2) | 0.4 (0.3, 0.6) | 177.77 (74.28,326.28) |
| Tunisia | 79.2 (72.2, 87.1) | 398.7 (367.4, 433.1) | 376.48 (318.20,438.65) | 7.8 (4.9, 12.3) | 52.9 (31.0, 81.4) | 530.88 (205.53,1126.24) | 0.3 (0.2, 0.5) | 2.4 (1.5, 3.8) | 604.88 (236.11,1313.25) |
| Turkmenistan | 27.6 (25.2, 30.5) | 83.7 (76.4, 91.3) | 192.48 (155.06,232.23) | 11.3 (8.3, 15.6) | 95.2 (64.9, 137.6) | 710.74 (364.95,1206.96) | 0.4 (0.3, 0.5) | 2.7 (1.8, 3.9) | 603.85 (304.85,1040.87) |
| Tuvalu | 0.0 (0.0, 0.0) | 0.0 (0.0, 0.0) | 77.89 (53.85,103.35) | 0.0 (0.0, 0.0) | 0.0 (0.0, 0.0) | 58.95 (-9.28,175.88) | 0.0 (0.0, 0.0) | 0.0 (0.0, 0.0) | 66.83 (-6.03,200.85) |
| Uganda | 15.3 (13.8, 17.0) | 55.2 (49.7, 61.4) | 216.25 (170.28,265.32) | 5.3 (3.7, 7.3) | 18.3 (12.0, 25.5) | 203.40 (75.99,392.57) | 0.2 (0.1, 0.3) | 0.6 (0.4, 0.9) | 184.07 (57.68,377.63) |
| Ukraine | 754.9 (685.3, 824.9) | 790.9 (717.7, 865.0) | 3.50 (-9.77,17.51) | 229.5 (165.7, 319.4) | 538.5 (342.9, 843.8) | 143.64 (39.45,297.35) | 7.9 (5.8, 10.9) | 15.6 (9.9, 23.5) | 105.45 (19.59,229.56) |
| United Arab Emirate | 28.2 (25.5, 31.8) | 536.5 (483.2, 602.3) | 1645.83 (1368.20,1943.74) | 2.5 (1.8, 3.5) | 57.3 (39.5, 81.5) | 1917.86 (1034.93,3247.15) | 0.1 (0.1, 0.1) | 1.8 (1.2, 2.6) | 1833.42 (977.71,3172.65) |
| United Kingdom | 2208.6 (2032.7, 2408.8) | 4658.5 (4295.3, 5032.4) | 102.23 (79.12,126.54) | 977.6 (746.5, 1239.8) | 3643.5 (2899.4, 4399.2) | 296.28 (180.39,447.42) | 36.1 (28.0, 45.4) | 138.5 (112.9, 166.1) | 308.93 (196.31,454.25) |
| United Republic of Tanzania | 36.7 (33.4, 40.5) | 135.6 (122.7, 151.1) | 226.21 (180.74,274.85) | 11.8 (8.6, 16.1) | 39.3 (26.5, 56.3) | 197.53 (71.95,377.61) | 0.4 (0.3, 0.6) | 1.3 (0.9, 1.9) | 197.85 (69.64,390.15) |
| United States of America | 12404.4 (11356.3, 13563.8) | 28489.1 (26004.0, 31074.2) | 123.76 (95.46,153.59) | 4672.5 (3550.6, 6129.0) | 13232.9 (10283.7, 16997.9) | 173.84 (83.73,297.11) | 174.1 (132.6, 224.7) | 531.2 (410.2, 671.2) | 192.36 (99.90,316.16) |
| United States Virgin Islands | 1.1 (1.0, 1.2) | 2.3 (2.1, 2.5) | 122.00 (93.93,152.20) | 0.8 (0.6, 1.2) | 1.7 (1.2, 2.3) | 132.89 (41.65,272.82) | 0.0 (0.0, 0.0) | 0.1 (0.0, 0.1) | 165.29 (63.41,317.90) |
| Uruguay | 42.7 (39.0, 47.0) | 70.7 (64.7, 77.0) | 60.22 (39.63,81.95) | 15.5 (10.9, 21.6) | 21.4 (15.8, 28.2) | 32.11 (-18.09,108.33) | 0.6 (0.4, 0.8) | 0.9 (0.7, 1.2) | 42.34 (-10.75,121.96) |
| Uzbekistan | 166.9 (152.5, 184.1) | 576.8 (524.6, 637.9) | 224.38 (180.55,270.93) | 57.5 (41.2, 78.3) | 271.8 (186.6, 379.4) | 346.67 (173.47,601.58) | 1.9 (1.3, 2.5) | 8.1 (5.6, 11.4) | 307.46 (151.67,540.17) |
| Vanuatu | 0.1 (0.1, 0.2) | 0.5 (0.5, 0.6) | 220.64 (175.77,268.18) | 0.0 (0.0, 0.1) | 0.1 (0.1, 0.2) | 205.34 (16.47,750.44) | 0.0 (0.0, 0.0) | 0.0 (0.0, 0.0) | 212.28 (19.03,763.14) |
| Venezuela (Bolivarian Republic of) | 36.2 (33.1, 39.9) | 112.7 (102.8, 124.0) | 210.39 (170.61,252.40) | 15.3 (11.4, 20.1) | 53.7 (34.3, 81.7) | 222.33 (81.83,413.08) | 0.5 (0.4, 0.7) | 1.9 (1.3, 2.9) | 236.08 (93.60,429.51) |
| Viet Nam | 72.6 (65.9, 80.2) | 252.7 (228.9, 277.0) | 236.50 (190.91,284.73) | 19.9 (13.4, 29.3) | 70.7 (48.1, 100.7) | 226.84 (87.13,457.08) | 0.8 (0.5, 1.1) | 2.7 (1.8, 3.8) | 226.71 (85.74,471.99) |
| Yemen | 44.3 (40.3, 49.0) | 264.5 (241.2, 292.4) | 439.21 (365.41,517.37) | 4.4 (2.3, 9.0) | 15.1 (9.3, 25.1) | 208.70 (11.57,901.39) | 0.2 (0.1, 0.3) | 0.6 (0.3, 1.0) | 216.65 (1.77,1086.13) |
| Zambia | 9.1 (8.2, 10.0) | 37.8 (34.3, 42.2) | 258.01 (206.58,312.80) | 4.6 (3.3, 6.5) | 12.8 (8.1, 19.1) | 143.34 (30.49,305.89) | 0.1 (0.1, 0.2) | 0.4 (0.3, 0.6) | 141.45 (31.05,317.97) |
| Zimbabwe | 13.9 (12.6, 15.4) | 36.4 (32.8, 40.4) | 139.53 (105.12,176.25) | 4.7 (3.3, 6.4) | 17.0 (9.8, 25.8) | 231.32 (66.39,464.04) | 0.2 (0.1, 0.2) | 0.6 (0.3, 0.8) | 202.88 (51.52,416.55) |

Numbers in parenthesis represent 95% uncertainty intervals (UIs). Numbers in parenthesis represent 95% confidence intervals (CIs). DALYs=disability-adjusted life year. IBD=inflammatory bowel disease. MASLD=metabolic dysfunction-associated steatotic liver disease. SDI=socio-demographic index.

**Table S6.** Age-standardized mortality rates (per 10,000,000 individuals) due to MASLD–IBD comorbidity in 1990 and 2021 and the EAPC from 1990 to 2021, stratified by SDI and region

| **Location** | **Age-standardized mortality rate** | | |
| --- | --- | --- | --- |
|  | **1990 ‱ (95% UI)** | **2021 ‱ (95% UI)** | **EAPC % (95% CI)** |
| **Global** | 1.9 (1.4, 2.4) | 1.9 (1.5, 2.3) | 0.07 (-0.09, 0.23) |
| **SDI** |  |  |  |
| High SDI | 4.7 (3.6, 6.1) | 5.0 (3.9, 6.2) | 0.21 (-0.03, 0.45) |
| High-middle SDI | 1.3 (1.0, 1.7) | 1.1 (0.9, 1.4) | -0.29 (-0.57, -0.02) |
| Middle SDI | 0.6 (0.4, 0.8) | 0.9 (0.7, 1.1) | 1.71 (1.55, 1.88) |
| Low-middle SDI | 1.2 (0.9, 1.7) | 1.5 (1.1, 2.0) | 1.03 (0.94, 1.12) |
| Low SDI | 1.0 (0.7, 1.4) | 1.0 (0.8, 1.3) | 0.09 (0.02, 0.15) |
| **Regions** |  |  |  |
| High-income Asia Pacific | 1.2 (0.9, 1.5) | 0.8 (0.6, 1.0) | -1.21 (-1.79, -0.62) |
| Central Asia | 2.6 (1.9, 3.4) | 4.0 (2.9, 5.3) | 1.70 (1.47, 1.93) |
| East Asia | 0.1 (0.1, 0.2) | 0.2 (0.2, 0.2) | 1.94 (1.31, 2.56) |
| South Asia | 1.1 (0.8, 1.6) | 1.5 (1.1, 2.0) | 1.24 (1.13, 1.34) |
| Southeast Asia | 0.2 (0.2, 0.3) | 0.3 (0.2, 0.3) | 0.63 (0.55, 0.71) |
| Australasia | 3.8 (2.9, 4.9) | 6.6 (5.3, 8.0) | 2.34 (2.04, 2.65) |
| Caribbean | 2.0 (1.4, 2.6) | 2.1 (1.5, 2.9) | 0.13 (-0.16, 0.43) |
| Central Europe | 2.3 (1.7, 3.0) | 2.9 (2.1, 3.9) | 0.56 (0.34, 0.78) |
| Eastern Europe | 0.9 (0.6, 1.1) | 2.4 (1.7, 3.3) | 3.55 (2.98, 4.12) |
| Western Europe | 7.9 (5.8, 10.2) | 7.0 (5.3, 8.8) | -0.57 (-0.89, -0.24) |
| Andean Latin America | 1.6 (1.1, 2.2) | 2.0 (1.4, 2.8) | 0.80 (0.64, 0.96) |
| Central Latin America | 0.6 (0.4, 0.8) | 0.7 (0.5, 0.9) | 0.65 (0.48, 0.81) |
| Southern Latin America | 2.2 (1.5, 3.0) | 2.3 (1.6, 3.1) | 0.50 (0.37, 0.63) |
| Tropical Latin America | 0.4 (0.3, 0.5) | 0.7 (0.5, 0.9) | 1.91 (1.41, 2.41) |
| North Africa and Middle East | 2.0 (1.3, 3.0) | 2.5 (1.8, 3.3) | 1.10 (0.91, 1.29) |
| High-income North America | 5.6 (4.2, 7.3) | 7.9 (6.1, 10.0) | 1.42 (1.28, 1.56) |
| Oceania | 0.1 (0.1, 0.2) | 0.1 (0.1, 0.2) | -0.71 (-0.85, -0.57) |
| Central Sub-Saharan Africa | 0.6 (0.4, 1.0) | 0.5 (0.3, 0.8) | -0.74 (-1.00, -0.48) |
| Eastern Sub-Saharan Africa | 0.7 (0.5, 0.9) | 0.8 (0.6, 1.0) | 0.29 (0.18, 0.41) |
| Southern Sub-Saharan Africa | 0.7 (0.5, 1.0) | 1.2 (1.0, 1.5) | 1.60 (1.08, 2.12) |
| Western Sub-Saharan Africa | 0.9 (0.6, 1.4) | 1.1 (0.9, 1.5) | 0.71 (0.66, 0.76) |

Numbers in parenthesis represent 95% uncertainty intervals (UIs). Numbers in parenthesis represent 95% confidence intervals (CIs). IBD=inflammatory bowel disease. EAPC=estimated annual percentage change. MASLD=metabolic dysfunction-associated steatotic liver disease. SDI=socio-demographic index.

**Table S7.** Age-standardized prevalence, DALY, and mortality rate (per 10,000,000 individuals) due to MASLD–IBD comorbidity in 1990 and 2021 among men and the EAPC from 1990 to 2021, stratified by SDI and region

| **Location** | **Age-standardized prevalence rate among men** | | | **Age-standardized DALY rate among men** | | | **Age-standardized mortality rate** | | |
| --- | --- | --- | --- | --- | --- | --- | --- | --- | --- |
|  | **1990 No. (95% UI)** | **2021 No. (95% UI)** | **EAPC % (95% CI)** | **1990 No. (95% UI)** | **2021 No. (95% UI)** | **EAPC % (95% CI)** | **1990 % (95% UI)** | **2021 % (95% UI)** | **EAPC % (95% CI)** |
| **Global** | 152.6 (139.9, 166.8) | 176.0 (161.0, 192.0) | 0.64 (0.54, 0.75) | 0.5 (0.4, 0.6) | 0.5 (0.4, 0.6) | 0.18 (-0.01, 0.38) | 1.8 (1.4, 2.3) | 1.9 (1.5, 2.3) | 0.22 (0.04, 0.40) |
| **SDI** |  |  |  |  |  |  |  |  |  |
| High SDI | 333.5 (305.8, 363.9) | 447.2 (409.9, 486.8) | 1.07 (0.89, 1.25) | 1.3 (1.0, 1.7) | 1.2 (1.0, 1.6) | -0.09 (-0.38, 0.20) | 4.6 (3.5, 6.0) | 4.7 (3.7, 6.0) | 0.09 (-0.17, 0.36) |
| High-middle SDI | 114.8 (105.1, 125.5) | 125.3 (114.6, 136.5) | 0.64 (0.42, 0.86) | 0.4 (0.3, 0.5) | 0.3 (0.3, 0.4) | -0.20 (-0.51, 0.12) | 1.4 (1.1, 1.8) | 1.2 (0.9, 1.5) | -0.38 (-0.63, -0.12) |
| Middle SDI | 48.6 (44.4, 53.2) | 80.2 (73.4, 87.4) | 1.97 (1.82, 2.12) | 0.2 (0.1, 0.2) | 0.3 (0.2, 0.3) | 1.85 (1.69, 2.01) | 0.6 (0.4, 0.7) | 0.9 (0.7, 1.2) | 1.94 (1.76, 2.13) |
| Low-middle SDI | 99.6 (90.9, 108.9) | 134.9 (123.6, 146.8) | 1.15 (1.09, 1.22) | 0.3 (0.2, 0.4) | 0.4 (0.3, 0.5) | 1.43 (1.30, 1.56) | 0.9 (0.7, 1.3) | 1.4 (1.1, 1.9) | 1.49 (1.38, 1.60) |
| Low SDI | 69.3 (63.4, 75.9) | 85.5 (78.1, 93.3) | 0.75 (0.69, 0.80) | 0.2 (0.2, 0.3) | 0.2 (0.2, 0.3) | -0.12 (-0.19, -0.04) | 0.8 (0.6, 1.1) | 0.8 (0.6, 1.1) | -0.04 (-0.11, 0.03) |
| **Regions** |  |  |  |  |  |  |  |  |  |
| High-income Asia Pacific | 81.8 (74.8, 89.2) | 120.5 (109.9, 131.5) | 1.57 (1.08, 2.07) | 0.3 (0.3, 0.4) | 0.2 (0.2, 0.3) | -1.66 (-2.18, -1.14) | 1.3 (1.1, 1.6) | 0.9 (0.7, 1.1) | -1.19 (-1.70, -0.67) |
| Central Asia | 167.6 (153.5, 183.4) | 187.9 (172.7, 205.8) | 0.46 (0.36, 0.56) | 0.6 (0.5, 0.9) | 1.0 (0.8, 1.4) | 1.81 (1.57, 2.05) | 2.4 (1.7, 3.2) | 3.7 (2.7, 4.9) | 1.74 (1.51, 1.97) |
| East Asia | 17.3 (15.8, 19.0) | 34.2 (31.2, 37.4) | 3.37 (2.79, 3.96) | 0.0 (0.0, 0.1) | 0.1 (0.0, 0.1) | 1.90 (1.29, 2.51) | 0.1 (0.1, 0.2) | 0.2 (0.2, 0.3) | 2.31 (1.65, 2.98) |
| South Asia | 125.1 (114.1, 137.1) | 173.7 (158.8, 189.5) | 1.19 (1.10, 1.28) | 0.3 (0.2, 0.4) | 0.4 (0.3, 0.5) | 1.09 (0.96, 1.22) | 0.9 (0.7, 1.4) | 1.4 (1.0, 1.9) | 1.44 (1.33, 1.55) |
| Southeast Asia | 18.1 (16.6, 19.9) | 20.9 (19.0, 22.7) | 0.59 (0.54, 0.64) | 0.1 (0.0, 0.1) | 0.1 (0.0, 0.1) | 0.86 (0.75, 0.97) | 0.2 (0.1, 0.3) | 0.2 (0.2, 0.3) | 0.99 (0.88, 1.11) |
| Australasia | 444.0 (404.9, 483.2) | 595.4 (543.4, 652.1) | 1.36 (1.06, 1.66) | 1.0 (0.7, 1.3) | 1.7 (1.3, 2.1) | 2.34 (1.99, 2.69) | 3.6 (2.7, 4.7) | 6.4 (5.1, 8.2) | 2.50 (2.18, 2.83) |
| Caribbean | 93.6 (85.7, 102.0) | 101.9 (93.4, 110.6) | 0.38 (0.33, 0.42) | 0.5 (0.3, 0.7) | 0.6 (0.4, 0.9) | 0.89 (0.56, 1.21) | 1.8 (1.3, 2.5) | 2.3 (1.6, 3.2) | 0.85 (0.55, 1.16) |
| Central Europe | 198.3 (181.0, 216.5) | 220.0 (201.3, 239.6) | 0.48 (0.31, 0.64) | 0.6 (0.5, 0.9) | 0.8 (0.6, 1.1) | 0.38 (0.10, 0.65) | 2.2 (1.6, 3.0) | 2.8 (2.0, 3.8) | 0.48 (0.24, 0.72) |
| Eastern Europe | 114.0 (104.7, 124.3) | 116.8 (107.3, 127.0) | 0.18 (-0.02, 0.39) | 0.3 (0.2, 0.4) | 0.9 (0.6, 1.2) | 3.58 (2.87, 4.29) | 1.0 (0.8, 1.4) | 2.5 (1.8, 3.5) | 3.03 (2.49, 3.58) |
| Western Europe | 355.2 (325.9, 387.9) | 507.8 (464.5, 552.0) | 1.13 (0.92, 1.35) | 2.1 (1.5, 2.8) | 1.8 (1.3, 2.4) | -0.59 (-0.92, -0.26) | 7.7 (5.6, 10.2) | 6.8 (5.1, 9.0) | -0.45 (-0.74, -0.16) |
| Andean Latin America | 44.1 (40.4, 48.1) | 53.3 (48.8, 58.1) | 0.65 (0.57, 0.74) | 0.4 (0.3, 0.6) | 0.5 (0.3, 0.7) | 0.70 (0.52, 0.89) | 1.4 (1.0, 2.0) | 1.9 (1.3, 2.8) | 1.02 (0.86, 1.18) |
| Central Latin America | 22.3 (20.4, 24.4) | 25.0 (22.9, 27.1) | 0.51 (0.39, 0.63) | 0.2 (0.1, 0.3) | 0.3 (0.2, 0.4) | 0.89 (0.70, 1.09) | 0.7 (0.5, 0.9) | 0.9 (0.6, 1.2) | 0.87 (0.67, 1.06) |
| Southern Latin America | 117.9 (107.2, 130.0) | 158.9 (145.2, 172.4) | 1.01 (0.96, 1.07) | 0.7 (0.5, 1.0) | 0.7 (0.5, 1.0) | 0.56 (0.37, 0.75) | 2.4 (1.6, 3.3) | 2.5 (1.8, 3.5) | 0.74 (0.56, 0.92) |
| Tropical Latin America | 47.1 (43.0, 51.6) | 81.5 (74.5, 89.1) | 1.43 (0.92, 1.95) | 0.1 (0.1, 0.2) | 0.2 (0.2, 0.3) | 1.81 (1.23, 2.40) | 0.4 (0.3, 0.5) | 0.8 (0.6, 1.0) | 2.13 (1.56, 2.70) |
| North Africa and Middle East | 167.0 (153.4, 181.4) | 254.9 (236.2, 275.1) | 1.81 (1.53, 2.08) | 0.3 (0.2, 0.5) | 0.5 (0.4, 0.7) | 1.84 (1.58, 2.11) | 1.5 (1.0, 2.2) | 2.2 (1.6, 3.0) | 1.68 (1.45, 1.92) |
| High-income North America | 482.5 (440.9, 527.0) | 591.1 (541.4, 644.3) | 0.82 (0.63, 1.00) | 1.5 (1.1, 2.0) | 1.9 (1.5, 2.5) | 1.04 (0.87, 1.22) | 5.3 (4.0, 6.9) | 7.3 (5.8, 9.0) | 1.21 (1.06, 1.37) |
| Oceania | 19.6 (17.8, 21.4) | 21.0 (19.3, 23.0) | 0.19 (0.14, 0.23) | 0.0 (0.0, 0.1) | 0.0 (0.0, 0.0) | -0.66 (-0.81, -0.51) | 0.1 (0.1, 0.2) | 0.1 (0.1, 0.2) | -0.62 (-0.77, -0.46) |
| Central Sub-Saharan Africa | 37.6 (34.0, 41.4) | 38.8 (35.5, 42.5) | -0.02 (-0.21, 0.18) | 0.2 (0.1, 0.3) | 0.1 (0.1, 0.2) | -0.84 (-1.13, -0.55) | 0.6 (0.4, 0.9) | 0.5 (0.3, 0.8) | -0.81 (-1.10, -0.52) |
| Eastern Sub-Saharan Africa | 34.6 (31.6, 37.9) | 43.5 (39.7, 47.5) | 0.58 (0.50, 0.67) | 0.1 (0.1, 0.2) | 0.2 (0.1, 0.2) | 0.19 (0.03, 0.35) | 0.5 (0.4, 0.7) | 0.6 (0.4, 0.8) | 0.18 (0.02, 0.35) |
| Southern Sub-Saharan Africa | 54.2 (49.7, 58.9) | 77.8 (71.3, 84.8) | 1.04 (0.99, 1.10) | 0.2 (0.1, 0.3) | 0.4 (0.3, 0.6) | 1.79 (1.12, 2.46) | 0.8 (0.5, 1.2) | 1.7 (1.3, 2.2) | 1.97 (1.33, 2.61) |
| Western Sub-Saharan Africa | 45.3 (41.4, 49.7) | 60.8 (55.7, 66.6) | 1.07 (1.00, 1.13) | 0.2 (0.1, 0.3) | 0.2 (0.2, 0.3) | 0.49 (0.43, 0.55) | 0.7 (0.5, 1.0) | 0.8 (0.6, 1.1) | 0.48 (0.42, 0.54) |

Numbers in parenthesis represent 95% uncertainty intervals (UIs). Numbers in parenthesis represent 95% confidence intervals (CIs). DALY=disability-adjusted life year. IBD=inflammatory bowel disease. EAPC=estimated annual percentage change. MASLD=metabolic dysfunction-associated steatotic liver disease. SDI=socio-demographic index.

**Table S8.** Age-standardized prevalence, DALY, and mortality rate (per 10,000,000 individuals) due to MASLD–IBD comorbidity in 1990 and 2021 among women and the EAPC from 1990 to 2021, stratified by SDI and region

| **Location** | **Age-standardized prevalence rate among women** | | | **Age-standardized DALY rate among women** | | | **Age-standardized mortality rate among women** | | |
| --- | --- | --- | --- | --- | --- | --- | --- | --- | --- |
|  | **1990 No. (95% UI)** | **2021 No. (95% UI)** | **EAPC % (95% CI)** | **1990 No. (95% UI)** | **2021 No. (95% UI)** | **EAPC % (95% CI)** | **1990 % (95% UI)** | **2021 % (95% UI)** | **EAPC % (95% CI)** |
| **Global** | 142.9 (130.5, 156.0) | 167.4 (153.4, 182.1) | 0.56 (0.45, 0.68) | 0.5 (0.4, 0.6) | 0.5 (0.4, 0.6) | -0.09 (-0.24, 0.06) | 1.9 (1.5, 2.5) | 1.8 (1.4, 2.3) | -0.04 (-0.19, 0.11) |
| **SDI** |  |  |  |  |  |  |  |  |  |
| High SDI | 229.8 (210.5, 249.7) | 327.4 (299.9, 356.1) | 1.23 (1.06, 1.41) | 1.3 (1.0, 1.7) | 1.4 (1.1, 1.7) | 0.30 (0.08, 0.51) | 4.8 (3.7, 6.2) | 5.2 (4.1, 6.5) | 0.34 (0.13, 0.56) |
| High-middle SDI | 105.4 (95.8, 115.0) | 123.7 (113.5, 134.3) | 0.61 (0.42, 0.79) | 0.3 (0.2, 0.4) | 0.3 (0.2, 0.4) | -0.06 (-0.41, 0.29) | 1.3 (1.0, 1.6) | 1.1 (0.9, 1.4) | -0.23 (-0.52, 0.07) |
| Middle SDI | 50.3 (46.0, 54.9) | 85.3 (77.9, 92.9) | 1.94 (1.79, 2.09) | 0.1 (0.1, 0.2) | 0.2 (0.2, 0.3) | 1.32 (1.18, 1.46) | 0.6 (0.4, 0.8) | 0.8 (0.6, 1.0) | 1.50 (1.33, 1.68) |
| Low-middle SDI | 94.0 (85.6, 102.6) | 125.5 (114.7, 136.6) | 1.14 (1.04, 1.23) | 0.3 (0.2, 0.5) | 0.4 (0.3, 0.5) | 0.84 (0.74, 0.93) | 1.5 (1.0, 2.2) | 1.7 (1.2, 2.2) | 0.67 (0.58, 0.76) |
| Low SDI | 61.5 (56.0, 67.2) | 76.2 (69.6, 83.4) | 0.78 (0.71, 0.86) | 0.3 (0.2, 0.4) | 0.3 (0.2, 0.4) | 0.05 (-0.01, 0.10) | 1.2 (0.8, 1.7) | 1.2 (0.9, 1.5) | 0.15 (0.08, 0.22) |
| **Regions** |  |  |  |  |  |  |  |  |  |
| High-income Asia Pacific | 37.5 (34.3, 41.0) | 51.1 (46.6, 55.9) | 1.33 (0.91, 1.75) | 0.2 (0.2, 0.3) | 0.1 (0.1, 0.2) | -1.66 (-2.30, -1.02) | 1.1 (0.8, 1.3) | 0.7 (0.5, 0.9) | -1.28 (-1.92, -0.64) |
| Central Asia | 165.8 (151.9, 182.0) | 187.1 (170.6, 205.0) | 0.50 (0.40, 0.61) | 0.7 (0.5, 0.9) | 1.1 (0.8, 1.5) | 1.47 (1.24, 1.70) | 2.7 (2.0, 3.5) | 4.1 (3.0, 5.6) | 1.67 (1.44, 1.89) |
| East Asia | 19.4 (17.6, 21.2) | 37.9 (34.6, 41.2) | 2.96 (2.36, 3.58) | 0.0 (0.0, 0.1) | 0.0 (0.0, 0.1) | 1.17 (0.59, 1.76) | 0.2 (0.1, 0.2) | 0.2 (0.1, 0.2) | 1.63 (1.01, 2.26) |
| South Asia | 119.4 (109.0, 131.0) | 158.8 (144.9, 173.5) | 1.12 (0.99, 1.25) | 0.3 (0.2, 0.5) | 0.4 (0.3, 0.6) | 0.91 (0.82, 1.01) | 1.3 (0.8, 1.9) | 1.6 (1.1, 2.3) | 1.02 (0.90, 1.14) |
| Southeast Asia | 21.5 (19.6, 23.4) | 24.5 (22.3, 26.8) | 0.54 (0.49, 0.58) | 0.1 (0.0, 0.1) | 0.1 (0.0, 0.1) | 0.07 (0.02, 0.12) | 0.3 (0.2, 0.4) | 0.3 (0.2, 0.4) | 0.34 (0.28, 0.40) |
| Australasia | 267.9 (242.6, 292.1) | 373.4 (339.5, 408.9) | 1.46 (1.16, 1.77) | 1.1 (0.9, 1.5) | 1.8 (1.4, 2.1) | 2.07 (1.76, 2.38) | 4.0 (3.1, 5.2) | 6.7 (5.3, 8.0) | 2.20 (1.90, 2.50) |
| Caribbean | 106.0 (96.9, 116.0) | 116.9 (107.1, 127.4) | 0.40 (0.35, 0.45) | 0.5 (0.4, 0.7) | 0.5 (0.3, 0.6) | -0.50 (-0.79, -0.21) | 2.2 (1.6, 2.9) | 1.8 (1.3, 2.5) | -0.63 (-0.91, -0.35) |
| Central Europe | 182.9 (166.8, 199.3) | 214.4 (195.5, 232.9) | 0.71 (0.52, 0.89) | 0.6 (0.5, 0.8) | 0.8 (0.6, 1.1) | 0.71 (0.46, 0.95) | 2.3 (1.7, 3.0) | 2.9 (2.2, 3.8) | 0.66 (0.45, 0.88) |
| Eastern Europe | 95.0 (86.2, 104.2) | 103.9 (94.5, 113.3) | 0.38 (0.21, 0.55) | 0.2 (0.2, 0.3) | 0.8 (0.6, 1.1) | 4.50 (3.75, 5.26) | 0.8 (0.6, 1.0) | 2.3 (1.7, 3.1) | 3.94 (3.35, 4.53) |
| Western Europe | 238.0 (217.5, 258.6) | 353.5 (324.1, 386.2) | 1.19 (0.94, 1.45) | 2.1 (1.6, 2.8) | 1.8 (1.4, 2.2) | -0.83 (-1.19, -0.47) | 8.0 (6.0, 10.3) | 7.1 (5.5, 8.6) | -0.68 (-1.02, -0.33) |
| Andean Latin America | 42.8 (39.0, 46.6) | 50.9 (46.5, 55.5) | 0.57 (0.46, 0.69) | 0.4 (0.3, 0.6) | 0.5 (0.3, 0.7) | 0.20 (-0.01, 0.41) | 1.8 (1.3, 2.3) | 2.2 (1.5, 2.9) | 0.61 (0.44, 0.79) |
| Central Latin America | 20.2 (18.5, 22.0) | 23.2 (21.2, 25.4) | 0.55 (0.46, 0.65) | 0.1 (0.1, 0.2) | 0.1 (0.1, 0.2) | 0.30 (0.15, 0.46) | 0.5 (0.4, 0.7) | 0.5 (0.4, 0.7) | 0.41 (0.26, 0.57) |
| Southern Latin America | 81.6 (74.0, 90.3) | 114.9 (103.7, 126.9) | 1.16 (1.11, 1.22) | 0.6 (0.4, 0.8) | 0.5 (0.4, 0.7) | 0.02 (-0.13, 0.16) | 2.1 (1.5, 2.8) | 2.0 (1.5, 2.7) | 0.24 (0.12, 0.37) |
| Tropical Latin America | 50.7 (46.4, 55.4) | 91.3 (83.8, 99.8) | 1.61 (1.15, 2.08) | 0.1 (0.1, 0.1) | 0.2 (0.1, 0.2) | 1.52 (1.08, 1.96) | 0.4 (0.3, 0.5) | 0.7 (0.5, 0.8) | 1.70 (1.26, 2.15) |
| North Africa and Middle East | 157.7 (144.4, 171.7) | 247.4 (228.6, 268.3) | 1.85 (1.57, 2.13) | 0.5 (0.3, 0.7) | 0.6 (0.4, 0.8) | 0.97 (0.77, 1.17) | 2.5 (1.6, 3.9) | 2.7 (1.9, 3.8) | 0.69 (0.51, 0.88) |
| High-income North America | 347.5 (315.1, 379.4) | 424.6 (387.4, 462.2) | 0.86 (0.67, 1.05) | 1.6 (1.2, 2.1) | 2.3 (1.8, 3.0) | 1.61 (1.42, 1.79) | 5.8 (4.3, 7.5) | 8.5 (6.6, 10.8) | 1.62 (1.46, 1.78) |
| Oceania | 21.0 (19.1, 23.0) | 22.1 (20.1, 24.2) | 0.16 (0.09, 0.23) | 0.0 (0.0, 0.1) | 0.0 (0.0, 0.0) | -0.91 (-1.05, -0.78) | 0.1 (0.1, 0.2) | 0.1 (0.1, 0.2) | -0.80 (-0.92, -0.68) |
| Central Sub-Saharan Africa | 35.6 (32.2, 39.4) | 36.8 (33.5, 40.5) | -0.06 (-0.28, 0.17) | 0.1 (0.1, 0.3) | 0.1 (0.1, 0.2) | -0.71 (-0.94, -0.48) | 0.6 (0.3, 1.0) | 0.5 (0.3, 0.8) | -0.70 (-0.93, -0.47) |
| Eastern Sub-Saharan Africa | 33.2 (30.3, 36.2) | 42.8 (39.1, 46.9) | 0.63 (0.54, 0.71) | 0.2 (0.1, 0.3) | 0.2 (0.2, 0.3) | 0.14 (0.03, 0.25) | 0.8 (0.6, 1.1) | 0.9 (0.7, 1.2) | 0.28 (0.18, 0.39) |
| Southern Sub-Saharan Africa | 48.3 (43.9, 53.0) | 63.2 (57.6, 68.9) | 0.69 (0.63, 0.75) | 0.2 (0.1, 0.2) | 0.2 (0.2, 0.3) | 1.26 (0.79, 1.73) | 0.6 (0.4, 0.9) | 0.9 (0.7, 1.1) | 1.29 (0.87, 1.72) |
| Western Sub-Saharan Africa | 41.3 (37.6, 45.1) | 59.5 (54.3, 65.3) | 1.17 (1.10, 1.24) | 0.3 (0.2, 0.4) | 0.3 (0.2, 0.4) | 0.74 (0.68, 0.80) | 1.1 (0.7, 1.7) | 1.4 (1.1, 1.8) | 0.86 (0.80, 0.93) |

Numbers in parenthesis represent 95% uncertainty intervals (UIs). Numbers in parenthesis represent 95% confidence intervals (CIs). DALY=disability-adjusted life year. IBD=inflammatory bowel disease. EAPC=estimated annual percentage change. MASLD=metabolic dysfunction-associated steatotic liver disease. SDI=socio-demographic index.

**Table S9.** Age-standardized prevalence, DALY, and mortality rate (per 10,000,000 individuals) due to MASLD–IBD comorbidity in 1990 and 2021 among adults aged 60 years and over and the EAPC from 1990 to 2021, stratified by SDI and region

| **Location** | **Prevalence rate among adults aged 60 and over** | | | **DALY rate among adults aged 60 and over** | | | **Age-standardized mortality rate** | | |
| --- | --- | --- | --- | --- | --- | --- | --- | --- | --- |
|  | **1990 No. (95% UI)** | **2021 No. (95% UI)** | **EAPC % (95% CI)** | **1990 No. (95% UI)** | **2021 No. (95% UI)** | **EAPC % (95% CI)** | **1990 % (95% UI)** | **2021 % (95% UI)** | **EAPC % (95% CI)** |
| **Global** | 857.2 (703.0, 1031.3) | 1032.3 (856.2, 1226.9) | 0.77 (0.62, 0.92) | 5.0 (3.5, 7.1) | 5.0 (3.7, 6.8) | 0.17 (0.06, 0.28) | 22.0 (15.4, 31.5) | 22.4 (16.4, 30.1) | 0.17 (0.06, 0.28) |
| **SDI** |  |  |  |  |  |  |  |  |  |
| High SDI | 1401.1 (1153.9, 1689.9) | 2012.3 (1667.3, 2398.7) | 1.33 (1.22, 1.44) | 10.5 (7.5, 14.7) | 12.1 (9.0, 15.9) | 0.44 (0.32, 0.56) | 46.5 (33.2, 64.7) | 54.0 (40.3, 71.4) | 0.43 (0.30, 0.56) |
| High-middle SDI | 597.3 (492.3, 716.5) | 623.2 (517.9, 742.2) | 0.25 (0.03, 0.48) | 3.3 (2.3, 4.6) | 2.3 (1.7, 3.1) | -1.11 (-1.31, -0.91) | 14.6 (10.3, 20.4) | 10.1 (7.5, 13.8) | -1.11 (-1.30, -0.92) |
| Middle SDI | 241.7 (198.3, 290.0) | 402.8 (334.8, 478.4) | 1.76 (1.68, 1.85) | 1.2 (0.8, 1.6) | 1.9 (1.4, 2.5) | 1.69 (1.53, 1.86) | 5.1 (3.6, 7.2) | 8.2 (6.1, 11.0) | 1.70 (1.52, 1.87) |
| Low-middle SDI | 515.2 (421.8, 622.0) | 710.4 (587.3, 846.1) | 1.16 (1.11, 1.22) | 2.8 (1.8, 4.3) | 4.1 (2.8, 5.7) | 1.30 (1.26, 1.34) | 12.5 (8.2, 19.5) | 18.1 (12.6, 25.6) | 1.29 (1.25, 1.33) |
| Low SDI | 355.5 (290.7, 428.9) | 472.1 (388.0, 565.7) | 1.01 (0.93, 1.08) | 2.6 (1.7, 4.0) | 2.8 (2.0, 4.0) | 0.22 (0.19, 0.25) | 11.7 (7.7, 17.6) | 12.5 (8.9, 17.7) | 0.23 (0.20, 0.26) |
| **Regions** |  |  |  |  |  |  |  |  |  |
| High-income Asia Pacific | 305.0 (248.7, 370.4) | 441.7 (367.8, 532.1) | 1.08 (0.84, 1.32) | 3.0 (2.2, 3.9) | 1.7 (1.3, 2.3) | -2.33 (-2.91, -1.74) | 13.2 (9.9, 17.3) | 7.8 (5.8, 10.3) | -2.28 (-2.88, -1.67) |
| Central Asia | 865.6 (715.4, 1040.9) | 909.5 (753.3, 1086.4) | 0.28 (0.14, 0.42) | 6.2 (4.2, 9.0) | 8.2 (5.6, 12.3) | 1.18 (0.97, 1.40) | 27.2 (18.5, 39.8) | 35.9 (24.5, 53.5) | 1.22 (0.97, 1.46) |
| East Asia | 81.5 (67.0, 98.3) | 168.3 (137.5, 202.5) | 3.05 (2.70, 3.39) | 0.3 (0.2, 0.4) | 0.4 (0.3, 0.5) | 1.85 (1.50, 2.19) | 1.3 (0.9, 1.7) | 1.7 (1.3, 2.3) | 1.83 (1.48, 2.18) |
| South Asia | 703.3 (572.3, 851.9) | 963.1 (789.6, 1160.4) | 1.04 (0.95, 1.13) | 2.8 (1.8, 4.4) | 3.9 (2.7, 5.8) | 1.05 (0.95, 1.16) | 12.3 (7.9, 19.5) | 17.5 (12.2, 25.6) | 1.08 (0.98, 1.18) |
| Southeast Asia | 85.8 (70.2, 103.2) | 99.8 (82.8, 119.2) | 0.57 (0.49, 0.64) | 0.4 (0.3, 0.7) | 0.5 (0.3, 0.7) | 0.45 (0.38, 0.52) | 1.9 (1.2, 3.0) | 2.2 (1.5, 3.0) | 0.47 (0.39, 0.55) |
| Australasia | 1791.9 (1469.9, 2192.3) | 2530.7 (2084.2, 3032.5) | 0.91 (0.74, 1.08) | 8.9 (6.3, 12.5) | 14.4 (11.0, 19.0) | 1.30 (1.05, 1.56) | 39.5 (28.0, 55.1) | 64.6 (49.1, 84.8) | 1.29 (1.03, 1.55) |
| Caribbean | 485.5 (400.6, 578.6) | 524.6 (438.9, 621.0) | 0.43 (0.29, 0.57) | 3.8 (2.5, 5.7) | 4.1 (2.6, 6.0) | 0.24 (-0.10, 0.59) | 17.0 (11.2, 25.7) | 18.1 (11.7, 26.6) | 0.22 (-0.12, 0.57) |
| Central Europe | 809.5 (667.6, 968.1) | 865.6 (722.3, 1024.0) | 0.47 (0.29, 0.65) | 4.1 (2.8, 6.0) | 4.9 (3.3, 7.1) | 0.60 (0.39, 0.81) | 17.9 (12.2, 26.1) | 21.5 (14.5, 31.3) | 0.60 (0.40, 0.80) |
| Eastern Europe | 663.4 (547.7, 796.7) | 646.1 (538.3, 760.4) | 0.08 (-0.21, 0.38) | 2.3 (1.6, 3.4) | 3.8 (2.6, 5.4) | 2.07 (1.62, 2.53) | 10.1 (7.0, 14.8) | 16.4 (11.4, 23.9) | 2.07 (1.64, 2.51) |
| Western Europe | 1195.1 (978.4, 1435.9) | 2060.9 (1718.0, 2455.3) | 1.77 (1.59, 1.94) | 14.3 (9.7, 20.3) | 14.9 (10.7, 20.1) | -0.13 (-0.34, 0.08) | 63.3 (42.8, 89.8) | 66.6 (47.8, 89.3) | -0.13 (-0.36, 0.10) |
| Andean Latin America | 231.2 (190.2, 277.3) | 281.3 (236.4, 334.3) | 0.67 (0.55, 0.78) | 3.5 (2.3, 5.5) | 5.2 (3.3, 7.8) | 1.27 (1.13, 1.40) | 15.7 (10.0, 24.4) | 23.2 (14.6, 34.8) | 1.28 (1.15, 1.41) |
| Central Latin America | 110.3 (92.1, 131.3) | 129.8 (109.1, 154.3) | 0.59 (0.53, 0.64) | 1.2 (0.8, 1.8) | 1.7 (1.2, 2.3) | 1.05 (0.90, 1.20) | 5.4 (3.6, 7.7) | 7.5 (5.3, 10.3) | 1.05 (0.89, 1.21) |
| Southern Latin America | 516.6 (416.4, 623.5) | 736.1 (604.3, 881.1) | 1.24 (1.18, 1.31) | 4.8 (3.0, 7.4) | 6.1 (4.0, 8.9) | 1.13 (0.97, 1.29) | 21.5 (13.5, 33.0) | 27.0 (17.9, 39.5) | 1.12 (0.96, 1.29) |
| Tropical Latin America | 248.5 (204.9, 297.1) | 507.8 (423.9, 603.7) | 1.96 (1.64, 2.27) | 0.7 (0.5, 1.0) | 1.8 (1.3, 2.4) | 3.01 (2.68, 3.34) | 3.0 (2.1, 4.4) | 7.9 (5.6, 10.8) | 3.02 (2.69, 3.34) |
| North Africa and Middle East | 631.3 (528.6, 748.3) | 1075.4 (920.5, 1242.0) | 2.19 (1.82, 2.56) | 3.4 (2.1, 5.4) | 5.5 (3.7, 7.9) | 2.03 (1.73, 2.34) | 15.4 (9.5, 24.5) | 24.4 (16.4, 35.5) | 1.99 (1.68, 2.30) |
| High-income North America | 2291.5 (1885.4, 2828.8) | 3202.2 (2627.7, 3869.4) | 1.39 (1.28, 1.50) | 13.5 (9.6, 19.2) | 21.6 (16.2, 28.7) | 1.65 (1.45, 1.86) | 59.9 (42.8, 84.4) | 95.9 (72.0, 127.5) | 1.62 (1.42, 1.81) |
| Oceania | 91.1 (74.4, 110.5) | 103.7 (85.4, 123.6) | 0.40 (0.33, 0.46) | 0.2 (0.1, 0.4) | 0.2 (0.1, 0.3) | -0.45 (-0.64, -0.26) | 1.0 (0.6, 1.8) | 0.9 (0.6, 1.5) | -0.46 (-0.66, -0.27) |
| Central Sub-Saharan Africa | 196.6 (158.9, 241.8) | 189.1 (154.7, 230.2) | -0.28 (-0.55, -0.01) | 1.5 (0.9, 2.6) | 1.2 (0.7, 2.1) | -0.99 (-1.29, -0.69) | 6.6 (3.8, 11.3) | 5.3 (3.1, 9.0) | -1.00 (-1.28, -0.71) |
| Eastern Sub-Saharan Africa | 191.8 (155.7, 230.8) | 241.4 (199.2, 288.9) | 0.56 (0.47, 0.65) | 1.9 (1.2, 2.7) | 2.2 (1.5, 3.2) | 0.18 (0.05, 0.32) | 8.3 (5.5, 12.1) | 9.7 (6.7, 14.0) | 0.19 (0.05, 0.32) |
| Southern Sub-Saharan Africa | 333.8 (272.8, 400.7) | 469.7 (388.6, 560.3) | 0.89 (0.80, 0.98) | 1.7 (1.0, 2.7) | 3.4 (2.5, 4.6) | 1.92 (1.38, 2.47) | 7.5 (4.6, 11.9) | 15.1 (11.2, 20.2) | 1.89 (1.34, 2.45) |
| Western Sub-Saharan Africa | 239.2 (194.8, 288.5) | 351.8 (289.4, 421.6) | 1.40 (1.27, 1.53) | 2.5 (1.5, 4.1) | 3.2 (2.3, 4.6) | 0.95 (0.83, 1.07) | 11.3 (6.9, 18.4) | 14.4 (10.3, 20.5) | 0.94 (0.82, 1.06) |

Numbers in parenthesis represent 95% uncertainty intervals (UIs). Numbers in parenthesis represent 95% confidence intervals (CIs). DALY=disability-adjusted life year. IBD=inflammatory bowel disease. EAPC=estimated annual percentage change. MASLD=metabolic dysfunction-associated steatotic liver disease. SDI=socio-demographic index.

**Table S10.** Age-standardized prevalence and DALY rates (per 10,000,000 individuals) due to MASLD–IBD comorbidity and Monte Carlo simulation in 2021, stratified by SDI and region

| **Location** | **Age-standardized prevalence rate** | | **Age-standardized DALY rate** | |
| --- | --- | --- | --- | --- |
|  | **2021 No. (95% UI)** | **2021 Monte Carlo simulation No. (95% UI)** | **2021 No. (95% UI)** | **2021 Monte Carlo simulation No. (95% UI)** |
| **Global** | 171.7 (157.3, 187.1) | 97.2 (26.6, 170.8) | 0.5 (0.4, 0.6) | 0.3 (0.3-0.5) |
| **SDI** |  |  |  |  |
| High SDI | 80.8 (73.8, 88.2) | 75.0 (20.6, 130.8) | 0.2 (0.2, 0.3) | 0.3 (0.3-0.5) |
| High-middle SDI | 130.2 (119.2, 141.6) | 100.3 (27.7, 175.5) | 0.4 (0.3, 0.5) | 0.2 (0.2-0.5) |
| Middle SDI | 82.8 (75.7, 90.2) | 107.5 (29.5, 188.5) | 0.2 (0.2, 0.3) | 0.3 (0.3-0.5) |
| Low-middle SDI | 124.7 (114.1, 136.0) | 102.1 (27.9, 178.4) | 0.3 (0.2, 0.4) | 0.3 (0.3-0.6) |
| Low SDI | 390.4 (358.0, 423.7) | 89.9 (24.8, 157.4) | 1.3 (1.0, 1.7) | 0.3 (0.3-0.5) |
| **Regions** |  |  |  |  |
| High-income Asia Pacific | 84.4 (77.4, 91.9) | 57.5 (15.8, 100.5) | 0.2 (0.1, 0.2) | 0.1 (0.1-0.2) |
| Central Asia | 187.2 (171.1, 204.5) | 104.3 (28.7, 181.5) | 1.1 (0.8, 1.5) | 0.6 (0.6-1.1) |
| East Asia | 36.1 (33.0, 39.3) | 100.9 (27.9, 176.1) | 0.0 (0.0, 0.1) | 0.1 (0.1-0.2) |
| South Asia | 166.6 (152.3, 181.7) | 91.8 (25.2, 161.3) | 0.4 (0.3, 0.5) | 0.2 (0.2-0.4) |
| Southeast Asia | 22.7 (20.7, 24.8) | 101.4 (27.9, 177.2) | 0.1 (0.0, 0.1) | 0.3 (0.3-0.5) |
| Australasia | 490.1 (448.6, 535.8) | 61.3 (17.0, 107.8) | 1.7 (1.4, 2.1) | 0.2 (0.2-0.4) |
| Caribbean | 109.7 (100.5, 119.0) | 101.2 (28.0, 176.7) | 0.6 (0.4, 0.8) | 0.5 (0.5-1.0) |
| Central Europe | 217.6 (198.6, 236.7) | 82.5 (22.8, 144.4) | 0.8 (0.6, 1.1) | 0.3 (0.3-0.6) |
| Eastern Europe | 110.8 (101.4, 120.4) | 79.4 (21.8, 139.4) | 0.8 (0.6, 1.1) | 0.6 (0.6-1.1) |
| Western Europe | 432.2 (396.2, 470.5) | 70.1 (19.3, 122.9) | 1.8 (1.4, 2.3) | 0.3 (0.3-0.5) |
| Andean Latin America | 52.1 (47.7, 56.9) | 96.9 (26.6, 169.5) | 0.5 (0.3, 0.7) | 0.9 (0.9-1.8) |
| Central Latin America | 24.1 (22.0, 26.2) | 110.0 (30.4, 191.9) | 0.2 (0.1, 0.3) | 0.9 (0.9-1.7) |
| Southern Latin America | 137.4 (125.4, 150.4) | 66.5 (18.4, 116.1) | 0.6 (0.4, 0.8) | 0.3 (0.3-0.5) |
| Tropical Latin America | 86.5 (79.1, 94.5) | 107.7 (29.7, 188.4) | 0.2 (0.1, 0.3) | 0.2 (0.2-0.5) |
| North Africa and Middle East | 251.6 (232.5, 271.8) | 179.9 (49.1, 314.1) | 0.5 (0.4, 0.7) | 0.4 (0.4-0.7) |
| High-income North America | 508.2 (464.3, 552.1) | 65.2 (17.9, 113.6) | 2.1 (1.7, 2.7) | 0.3 (0.3-0.5) |
| Oceania | 21.5 (19.8, 23.5) | 98.1 (27.0, 172.0) | 0.0 (0.0, 0.0) | 0.1 (0.1-0.3) |
| Central Sub-Saharan Africa | 37.9 (34.6, 41.3) | 77.0 (21.1, 134.1) | 0.1 (0.1, 0.2) | 0.3 (0.3-0.6) |
| Eastern Sub-Saharan Africa | 43.2 (39.5, 47.2) | 85.3 (23.3, 149.3) | 0.2 (0.1, 0.3) | 0.4 (0.4-0.7) |
| Southern Sub-Saharan Africa | 70.1 (64.1, 76.5) | 103.3 (28.4, 180.7) | 0.3 (0.3, 0.4) | 0.5 (0.5-0.9) |
| Western Sub-Saharan Africa | 60.3 (55.1, 66.0) | 96.9 (26.6, 169.0) | 0.3 (0.2, 0.3) | 0.4 (0.4-0.8) |

Numbers in parenthesis represent 95% uncertainty intervals (UIs). DALY=disability-adjusted life year. IBD=inflammatory bowel disease. MASLD=metabolic dysfunction-associated steatotic liver disease. SDI=socio-demographic index.

**Table S11.** Goodness-of-fit assessment and cross-validation of BAPC for prevalence

| **Age Group** | **Goodness-of-Fit Evaluation (1990-2020)** | | | **Cross-Validation (2021)** | | |
| --- | --- | --- | --- | --- | --- | --- |
|  | **RMSE (per 10,000,000 individuals)** | ***R*²** | ***p*-value** | **Actual 2021 (per 10,000,000 individuals)** | **Predicted 2021 (per 10,000,000 individuals)** | **Residual** |
| 15-19 years | 4.01 | 0.995 | 1.71e-35 | 1,078.3 | 1,077.4 | 1.0 |
| 20-24 years | 17.60 | 0.978 | 1.87e-26 | 2,137.0 | 2,137.0 | 0.0 |
| 25-29 years | 30.90 | 0.975 | 1.63e-25 | 3,198.4 | 3,224.3 | -25.9 |
| 30-34 years | 59.50 | 0.945 | 1.82e-20 | 3,887.0 | 3,886.9 | 0.1 |
| 35-39 years | 84.40 | 0.916 | 1.05e-17 | 4,297.5 | 4,239.1 | 58.4 |
| 40-44 years | 95.30 | 0.915 | 1.32e-17 | 4,822.8 | 4,736.9 | 86.0 |
| 45-49 years | 105.00 | 0.915 | 1.25e-17 | 5,313.5 | 5,207.3 | 106.2 |
| 50-54 years | 109.00 | 0.913 | 1.94e-17 | 5,544.7 | 5,429.2 | 115.6 |
| 55-59 years | 119.00 | 0.896 | 2.70e-16 | 5,860.1 | 5,693.6 | 166.5 |
| 60-64 years | 130.00 | 0.883 | 1.65e-15 | 6,226.9 | 6,068.0 | 158.9 |
| 65-69 years | 112.00 | 0.921 | 4.44e-18 | 6,820.7 | 6,628.9 | 191.9 |
| 70-74 years | 80.70 | 0.961 | 1.17e-22 | 7,282.2 | 7,215.9 | 66.3 |
| 75-79 years | 68.40 | 0.975 | 1.66e-25 | 7,717.3 | 7,663.4 | 53.9 |
| 80-84 years | 50.80 | 0.984 | 1.62e-28 | 7,366.4 | 7,324.4 | 42.0 |
| 85-89 years | 36.80 | 0.987 | 4.82e-30 | 6,161.5 | 6,093.2 | 68.3 |
| 90-94 years | 17.20 | 0.996 | 1.14e-37 | 5,285.5 | 5,254.7 | 30.8 |
| 95+ years | 16.40 | 0.996 | 8.02e-38 | 5,051.3 | 5,108.3 | -57.0 |

Model performance metrics (RMSE and *R*²) were derived from fitting procedures using 1990-2020 data. Cross-validation was conducted by comparing 2021 model predictions against actual GBD 2021 observations. Due to the absence of reliable estimates for the 0-14 years age group in the GBD database, our analysis was confined to the 15-95+ years cohort. Age cohorts were represented by their median values (e.g., 15-19 years as 17.5 years), with the 95+ years category processed as 95-99 years to maintain epidemiological consistency. All prevalence rates are expressed as cases per 100,000 population. The accompanying *p*-values denote the statistical significance of the linear regression parameter estimates.

**References**

[1] Rinella ME, Sookoian S (2024) From NAFLD to MASLD: updated naming and diagnosis criteria for fatty liver disease. J Lipid Res 65(1): 100485.

[2] Kaylan KB, Paul S (2024) NAFLD No More: A Review of Current Guidelines in the Diagnosis and Evaluation of Metabolic Dysfunction-Associated Steatotic Liver Disease (MASLD). Curr Diab Rep 25(1): 5.

[3] Fernández-Barrena MG, Avila MA (2023) Frontiers in fatty liver: recent advances in pathogenic mechanisms, assessment of patients' prognosis and pharmacotherapy : MASLD: new pathogenic mechanisms, risk assessment tools and drug therapies. Journal of physiology and biochemistry 79(4): 811-813.

[4] Bajo FR, Shipman KE (2023) Reclassification of nonalcoholic fatty liver disease: a multi-society Delphi consensus statement. Clinical and experimental dermatology 48(12): 1418-1421.

[5] Abdelnabi MN, Hassan GS, Shoukry NH (2024) Role of the type 3 cytokines IL-17 and IL-22 in modulating metabolic dysfunction-associated steatotic liver disease. Front Immunol 15: 1437046.

[6] Boldys A, Buldak L (2024) Metabolic dysfunction-associated steatotic liver disease: Navigating terminological evolution, diagnostic frontiers and therapeutic horizon-an editorial exploration. World J Gastroenterol 30(18): 2387-2390.

[7] Torres J, Mehandru S, Colombel JF, Peyrin-Biroulet L (2017) Crohn's disease. Lancet 389(10080): 1741-1755.

[8] Ordás I, Eckmann L, Talamini M, Baumgart DC, Sandborn WJ (2012) Ulcerative colitis. Lancet 380(9853): 1606-1619.

[9] Ananthakrishnan AN, Bernstein CN, Iliopoulos D, Macpherson A, Neurath MF, et al. (2018) Environmental triggers in IBD: a review of progress and evidence. Nat Rev Gastroenterol Hepatol 15(1): 39-49.

[10] de Lange KM, Moutsianas L, Lee JC, Lamb CA, Luo Y, et al. (2017) Genome-wide association study implicates immune activation of multiple integrin genes in inflammatory bowel disease. Nat Genet 49(2): 256-261.

[11] Hagström H, Adams LA, Allen AM, Byrne CD, Chang Y, et al. (2024) The future of International Classification of Diseases coding in steatotic liver disease: An expert panel Delphi consensus statement. Hepatology communications 8(2).

[12] Hayward KL, Johnson AL, Horsfall LU, Moser C, Valery PC, et al. (2021) Detecting non-alcoholic fatty liver disease and risk factors in health databases: accuracy and limitations of the ICD-10-AM. BMJ open gastroenterology 8(1).

[13] Hutfless S, Jasper RA, Tilak A, Ghosh T, Kedia S, et al. (2023) A Systematic Review of Crohn's Disease Case Definitions in Administrative or Claims Databases. Inflammatory bowel diseases 29(5): 705-715.

[14] Kumric M, Ticinovic Kurir T, Martinovic D, Zivkovic PM, Bozic J (2021) Impact of the COVID-19 pandemic on inflammatory bowel disease patients: A review of the current evidence. World journal of gastroenterology 27(25): 3748-3761.

[15] Islami F, Goding Sauer A, Miller KD, Siegel RL, Fedewa SA, et al. (2018) Proportion and number of cancer cases and deaths attributable to potentially modifiable risk factors in the United States. CA: a cancer journal for clinicians 68(1): 31-54.

[16] (2024) Burden of disease scenarios for 204 countries and territories, 2022-2050: a forecasting analysis for the Global Burden of Disease Study 2021. Lancet (London, England) 403(10440): 2204-2256.

[17] Zhang Q, Liu S, Wu J, Zhu S, Wu Y, et al. (2024) Non-alcoholic fatty liver degree and long-term risk of incident inflammatory bowel disease: A large-scale prospective cohort study. Chin Med J (Engl) 137(14): 1705-1714.

[18] Lee S, Moon S, Kim K, Sung S, Hong Y, et al. (2024) A Comparison of Green, Delta, and Monte Carlo Methods to Select an Optimal Approach for Calculating the 95% Confidence Interval of the Population-attributable Fraction: Guidance for Epidemiological Research. Journal of preventive medicine and public health = Yebang Uihakhoe chi 57(5): 499-507.

[19] Fong SJ, Li G, Dey N, Crespo RG, Herrera-Viedma E (2020) Composite Monte Carlo decision making under high uncertainty of novel coronavirus epidemic using hybridized deep learning and fuzzy rule induction. Applied soft computing 93: 106282.

[20] (2022) Global, regional, and national burden of colorectal cancer and its risk factors, 1990-2019: a systematic analysis for the Global Burden of Disease Study 2019. The lancet Gastroenterology & hepatology 7(7): 627-647.

[21] Zhang W, Cao G, Wu F, Wang Y, Liu Z, et al. (2023) Global Burden of Prostate Cancer and Association with Socioeconomic Status, 1990-2019: A Systematic Analysis from the Global Burden of Disease Study. Journal of epidemiology and global health 13(3): 407-421.

[22] Chicco D, Warrens MJ, Jurman G (2021) The coefficient of determination R-squared is more informative than SMAPE, MAE, MAPE, MSE and RMSE in regression analysis evaluation. PeerJ Computer science 7: e623.

[23] Cheng H, Garrick DJ, Fernando RL (2017) Efficient strategies for leave-one-out cross validation for genomic best linear unbiased prediction. Journal of animal science and biotechnology 8: 38.

[24] Geroldinger A, Lusa L, Nold M, Heinze G (2023) Leave-one-out cross-validation, penalization, and differential bias of some prediction model performance measures-a simulation study. Diagnostic and prognostic research 7(1): 9.

[25] Mullah MAS, Hanley JA, Benedetti A (2019) Modeling perinatal mortality in twins via generalized additive mixed models: a comparison of estimation approaches. BMC medical research methodology 19(1): 209.

[26] Gauthier J, Wu QV, Gooley TA (2020) Cubic splines to model relationships between continuous variables and outcomes: a guide for clinicians. Bone marrow transplantation 55(4): 675-680.
